# The intrinsic substrate specificity of the human tyrosine kinome

**DOI:** 10.1038/s41586-024-07407-y

**Published:** 2024-05-08

**Authors:** Tomer M. Yaron-Barir, Brian A. Joughin, Emily M. Huntsman, Alexander Kerelsky, Daniel M. Cizin, Benjamin M. Cohen, Amit Regev, Junho Song, Neil Vasan, Ting-Yu Lin, Jose M. Orozco, Christina Schoenherr, Cari Sagum, Mark T. Bedford, R. Max Wynn, Shih-Chia Tso, David T. Chuang, Lei Li, Shawn S.-C. Li, Pau Creixell, Konstantin Krismer, Mina Takegami, Harin Lee, Bin Zhang, Jingyi Lu, Ian Cossentino, Sean D. Landry, Mohamed Uduman, John Blenis, Olivier Elemento, Margaret C. Frame, Peter V. Hornbeck, Lewis C. Cantley, Benjamin E. Turk, Michael B. Yaffe, Jared L. Johnson

**Affiliations:** 1 Meyer Cancer Center, Weill Cornell Medicine, New York, NY USA; 2 Englander Institute for Precision Medicine, Institute for Computational Biomedicine, Weill Cornell Medicine, New York, NY USA; 3 Columbia University Vagelos College of Physicians and Surgeons, New York, NY USA; 4 David H. Koch Institute for Integrative Cancer Research, Massachusetts Institute of Technology, Cambridge, MA USA; 5 Center for Precision Cancer Medicine, Massachusetts Institute of Technology, Cambridge, MA USA; 6 Department of Biological Engineering, Massachusetts Institute of Technology, Cambridge, MA USA; 7 Department of Biology, Massachusetts Institute of Technology, Cambridge, MA USA; 8 Department of Medicine, Division of Hematology/Oncology, Columbia University Irving Medical Center, New York, NY USA; 9 Department of Discovery Technologies, Calico Life Sciences, South San Francisco, CA USA; 10 Department of Cell Biology, Harvard Medical School, Boston, MA USA; 11 Dana-Farber Cancer Institute, Harvard Medical School, Boston, MA USA; 12 Cancer Research United Kingdom Scotland Centre, Institute of Genetics and Molecular Medicine, University of Edinburgh, Edinburgh, UK; 13 Department of Epigenetics and Molecular Carcinogenesis, The University of Texas MD Anderson Cancer Center, Houston, TX USA; 14 Department of Biochemistry, University of Texas Southwestern Medical Center, Dallas, TX USA; 15 Department of Internal Medicine, University of Texas Southwestern Medical Center, Dallas, TX USA; 16 School of Health and Life Sciences, University of Health and Rehabilitation Sciences, Qingdao, China; 17 Department of Biochemistry, Schulich School of Medicine and Dentistry, Western University, London, Canada; 18 Cancer Research UK Cambridge Institute, University of Cambridge Li Ka Shing Centre, Cambridge, UK; 19 Computer Science and Artificial Intelligence Laboratory, Massachusetts Institute of Technology, Cambridge, MA USA; 20 Department Of Bioinformatics, Cell Signaling Technology, Danvers, MA USA; 21 Department of Pharmacology, Weill Cornell Medicine, New York, NY USA; 22 Department of Biochemistry, Weill Cornell Medicine, New York, NY USA; 23 Department of Pharmacology, Yale School of Medicine, New Haven, CT USA; 24 Division of Acute Care Surgery, Trauma, and Surgical Critical Care, and Division of Surgical Oncology, Department of Surgery, Beth Israel Deaconess Medical Center, Harvard Medical School, Boston, MA USA

**Keywords:** Kinases, Cellular signalling networks, Bioinformatics, Phosphorylation

## Abstract

Phosphorylation of proteins on tyrosine (Tyr) residues evolved in metazoan organisms as a mechanism of coordinating tissue growth^1^. Multicellular eukaryotes typically have more than 50 distinct protein Tyr kinases that catalyse the phosphorylation of thousands of Tyr residues throughout the proteome^1–3^. How a given Tyr kinase can phosphorylate a specific subset of proteins at unique Tyr sites is only partially understood^4–7^. Here we used combinatorial peptide arrays to profile the substrate sequence specificity of all human Tyr kinases. Globally, the Tyr kinases demonstrate considerable diversity in optimal patterns of residues surrounding the site of phosphorylation, revealing the functional organization of the human Tyr kinome by substrate motif preference. Using this information, Tyr kinases that are most compatible with phosphorylating any Tyr site can be identified. Analysis of mass spectrometry phosphoproteomic datasets using this compendium of kinase specificities accurately identifies specific Tyr kinases that are dysregulated in cells after stimulation with growth factors, treatment with anti-cancer drugs or expression of oncogenic variants. Furthermore, the topology of known Tyr signalling networks naturally emerged from a comparison of the sequence specificities of the Tyr kinases and the SH2 phosphotyrosine (pTyr)-binding domains. Finally we show that the intrinsic substrate specificity of Tyr kinases has remained fundamentally unchanged from worms to humans, suggesting that the fidelity between Tyr kinases and their protein substrate sequences has been maintained across hundreds of millions of years of evolution.

## Abstract (web summary)

An atlas of the substrate specificities for the human tyrosine kinome reveals diversity of motif specificities and enables identification of kinase–substrate relationships and kinase regulation in phosphoproteomics experiments.

## Main

Protein Tyr kinase signalling is an integral part of cellular communication in metazoan organisms^1^. The human protein Tyr kinome comprises a functionally diverse family of signalling proteins that orchestrate a wide variety of biological processes, including cell migration, cell survival, cell proliferation, nutrient uptake, response to pathogens and almost all stages of embryonic development. Aberrant Tyr kinase signalling is associated with human disease and is a frequent driver of cancer^8–10^. Indeed, the first oncogene identified (SRC) was also the first Tyr kinase to be discovered^11,12^, and over 50 Tyr kinase inhibitors—including Gleevec, one of the earliest successful molecular medicines—are now FDA-approved cancer therapies^13,14^.

Classical phosphotyrosine signalling cascades are initiated at the cell membrane through receptor Tyr kinases (RTKs)^4,15^ or transmembrane proteins with associated non-receptor Tyr kinases (nRTKs)^5^ that phosphorylate nearby Tyr residues and create binding sites for protein interaction modules, most prominently including SRC homology 2 (SH2) domains^16–18^, that further propagate the signal. Well-characterized signalling cascades involve only a small fraction of the more than 40,000 unique Tyr phosphorylation sites reported to date^2,3,19^. Accordingly, our knowledge of Tyr kinase signalling just scratches the surface of a vastly more complex set of phosphorylation networks. Our ability to define these networks is hampered by our limited understanding of the rules that govern their organization, motivating an examination of the phosphorylation site specificities of all Tyr kinases.

## Motif specificity of Tyr kinases

**Fig. 1:**
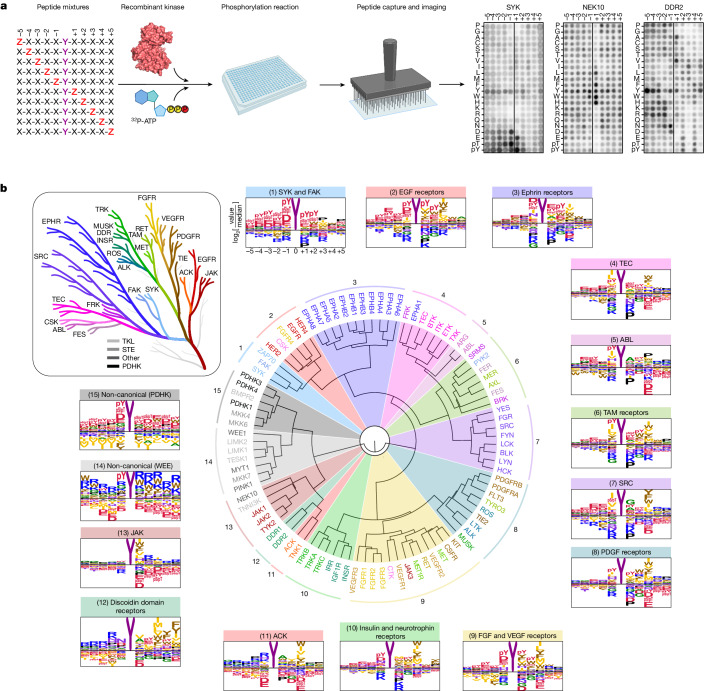
Profiling optimal phosphorylation motifs reveals sequence specificity of the human Tyr kinome. **a**, Experimental workflow for the PSPA analysis and representative results. Z denotes fixed positions containing one of the 20 natural amino acids, phosphorylated Thr (pT) or phosphorylated Tyr (pY). X denotes unfixed positions containing randomized mixtures of all natural amino acids except for Tyr and Cys. Autoradiograms (right) indicate kinase preferences for specific amino acids at each position; darker spots indicate preferred residues. **b**, Hierarchical clustering of 93 Tyr kinases on the basis of their amino acid motif selectivity determined from the quantified PSPA data. Kinase names are colour coded according to catalytic domain sequence phylogeny (inset)^21^. The diagram in **a** was created using BioRender.

**Extended Data Fig. 1:**
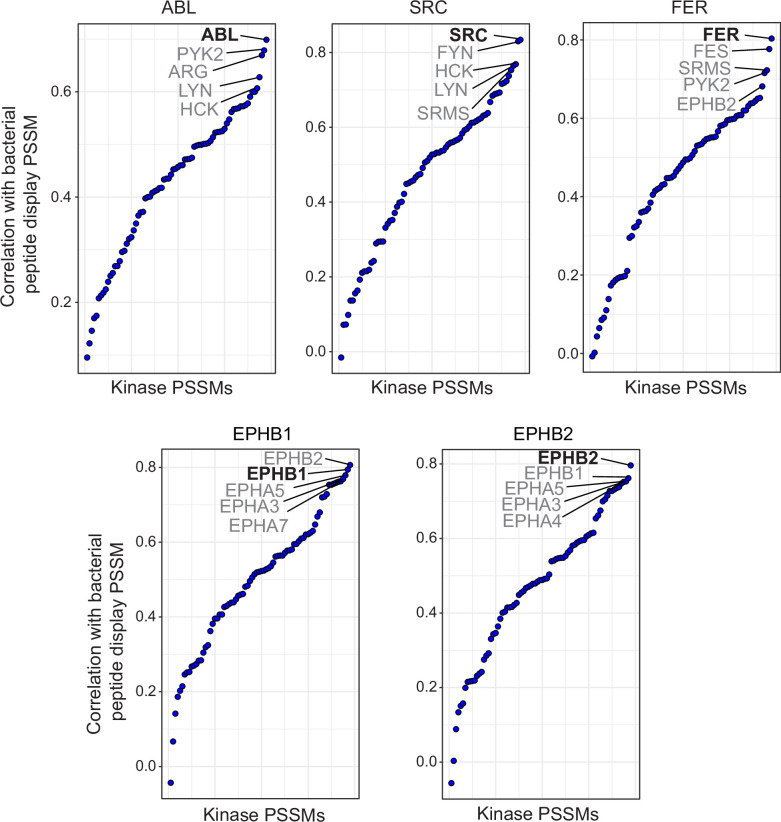
Correlation between Tyr kinase PSSMs derived from PSPA assays and bacterial peptide display. Pearson correlation coefficients for position specific scoring matrices (PSSMs) obtained previously for five kinases screened by bacterial display^7^ were calculated against the PSSMs of the 78 conventional RTKs and nRTKs obtained in this study. Correlation coefficients are sorted from lowest to highest with each of the 5 kinases screened by bacterial display with the 5 best-matching kinase selectivities in our study explicitly labelled.

To better understand how Tyr kinases connect to their downstream effectors, we profiled the substrate specificity of the entire collection of human Tyr kinases. Positional scanning peptide arrays (PSPA) were used to profile the phosphorylation site motifs of the human Tyr kinome using a combinatorial peptide library method that we previously applied to the human serine/threonine (Ser/Thr) kinome^20^ (Fig. 1a). Using recombinant kinase preparations, we successfully obtained phosphorylation site sequence motifs for all 78 catalytically active conventional Tyr kinases^21^ (Supplementary Fig. 1 and Supplementary Tables 1 and 2). These motifs were strongly concordant with those obtained previously for a handful of kinases using different experimental approaches^7^ (Extended Data Fig. 1). Moreover, we defined Tyr phosphorylation motifs for 15 Ser/Thr kinases that displayed convergent Tyr phosphorylation activity, including known dual-specificity kinases in the WEE, LIMK and NEK families^22–25^, as well as new Ser/Thr kinases that we identified could also phosphorylate Tyr, including the mitophagy kinase PINK1, the cardiac kinase TNNI3K and the mitochondrial pyruvate dehydrogenase kinases (PDHKs)^20^.

**Extended Data Fig. 2:**
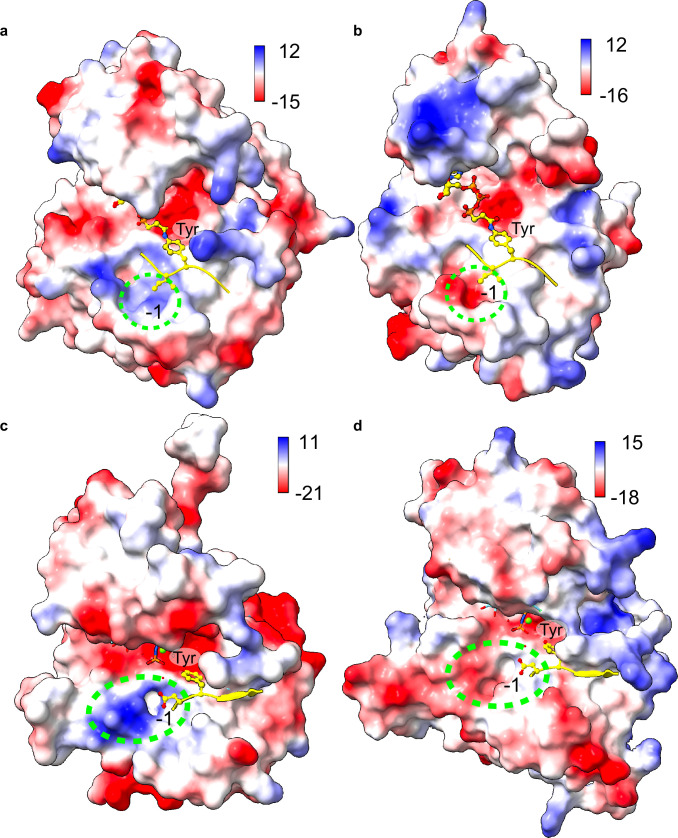
Structural models of kinase-substrate complexes. **a**, EGFR (PDB: 2GS6) in complex with synthetic peptide. Dotted green circle shows positive surface potential in the vicinity of the −1 residue. **b**, Synthetic peptide from its complex with EGFR (PDB: 2GS6) modelled onto ACK (PDB: 1U46). Dotted green circle shows negative surface potential in the vicinity of the −1 residue. **c**, INSR (PDB: 1IR3) in complex with synthetic peptide. Dotted green circle shows positive surface potential in the vicinity of the substrate N-terminal residues. **d**, Synthetic peptide from its complex with INSR (PDB: 1IR3) modelled onto DDR2 (PDB: AF-Q16832-K1A)^80^. Dotted green circle shows negative surface potential in the vicinity of the substrate N-terminal residues. Surface electrostatics are represented with Coulombic potential values were computed in ChimeraX and represented by scale bars (kcal/mol·e). In all panels, “Tyr” represents the site of phosphorylation and “−1” indicates the residue directly to the N-terminal side of the site of phosphorylation.

Contrary to general belief^26^, the Tyr kinases show a high degree of selectivity for the amino acids near the phosphorylated Tyr residues (Supplementary Fig. 1). To compare substrate specificities across the human Tyr kinome, we performed hierarchical clustering using quantified PSPA data across all positions within the peptide sequence (Fig. 1b). On the basis of this analysis, we categorized the kinome into 15 distinct clusters. These specificity groups spanned a continuum from acidophilic kinases selecting negatively charged residues surrounding their Tyr sites (including FAK (encoded by *PTK2*; cluster 1) and EGF receptor (EGFR; cluster 2)) to basophilic Tyr kinases that select for positively charged amino acids—a phenomenon not generally observed in Tyr kinases. Basophilic kinases included ACK (cluster 11) and discoidin domain receptor (cluster 12), both of which had substrate-complementary negatively charged regions within their catalytic domains (Extended Data Fig. 2). Between these two extremes, the clusters included kinases recognizing various position-specific combinations of hydrophobic, acidic, polar and small side-chain residues. Clustering by substrate specificity did not strictly recapitulate kinase domain sequence phylogeny^21,27^. In several cases, closely related Tyr kinases unexpectedly diverged in specificity and phosphorylated distinct sequence motifs (Fig. 1b). For example, nearest-neighbour paralogues FAK and PYK2 recognized acidic and hydrophobic motifs, respectively (Supplementary Fig. 1). This observation is consistent with their largely distinct sets of reported substrates and rationalizes the inability of PYK2 expression to rescue the phenotypes of FAK-null cells, although their distinct non-catalytic domains may also contribute to these differences^28,29^. Similarly, the motif for JAK3 clustered far apart in specificity space from its phylogenetic paralogues JAK1, JAK2 and TYK2, consistent with its divergent biological roles^30^.

**Extended Data Fig. 3:**
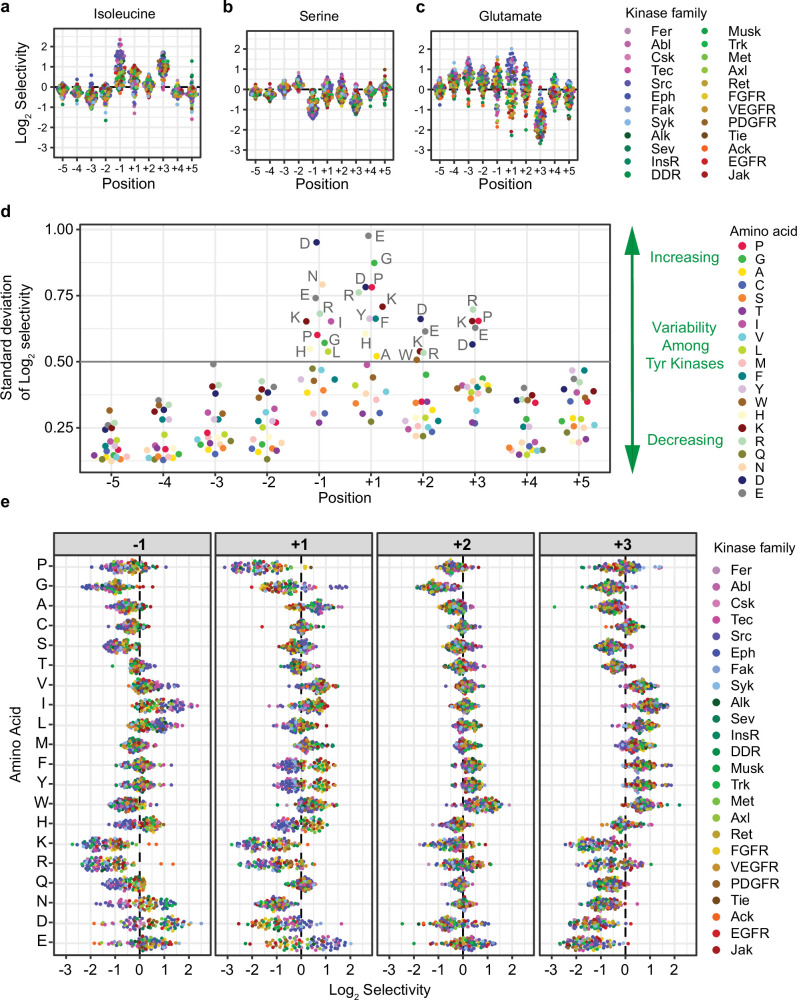
Human Tyr kinases display strong selectivities and diverse preferences for the amino acids near their Tyr phosphorylation sites. **a-c**, Log-selectivity of the 78 conventional RTKs and nRTKs on PSPA substrate peptides containing Tyr sites flanked by isoleucine (**a**), serine (**b**), or glutamate (**c**) residues relative to the 18 natural amino acids excluding cysteine and tyrosine. **d**, Kinome-wide variability in log-selectivity for specific amino acid residues at each position surrounding the substrate Tyr phosphorylation site. Horizontal line indicates a value of 0.5 logs, identifying positions −1 to +3 as the most variably selective positions. **e**, Experimental kinase selectivity for each Tyr kinase on all amino acids across the highly selective substrate positions −1 to +3.

We found a greater diversity in the phosphorylation-site specificity within the complete Tyr kinome than expected. Selectivity was predominantly observed in positions −1 to +3 relative to the phosphoacceptor Tyr (Extended Data Fig. 3). Some preferences were common to essentially all conventional Tyr kinases. For example, Tyr kinases generally selected aliphatic hydrophobic residues such as isoleucine in the −1 and +3 positions (Extended Data Fig. 3a) while disfavouring serine at the −1 (Extended Data Fig. 3b) and glutamate at the +3 (Extended Data Fig. 3c) positions. However, at each position, there were specific residues that distinguished the various clusters from one another (Extended Data Fig. 3d). Notably, a glutamate residue at position +1 broadly divided the kinome into two large groups, with most nRTKs favouring and most RTKs disfavouring it (Extended Data Fig. 3c). At other positions, specific residue preferences uniquely identified a small number of individual kinases. For example, only four kinases, including both ABL isoforms, strongly selected proline in the +3 position. Similarly, the ACK kinases uniquely favour basic residues at the −1 position (Extended Data Fig. 3e).

**Extended Data Fig. 4:**
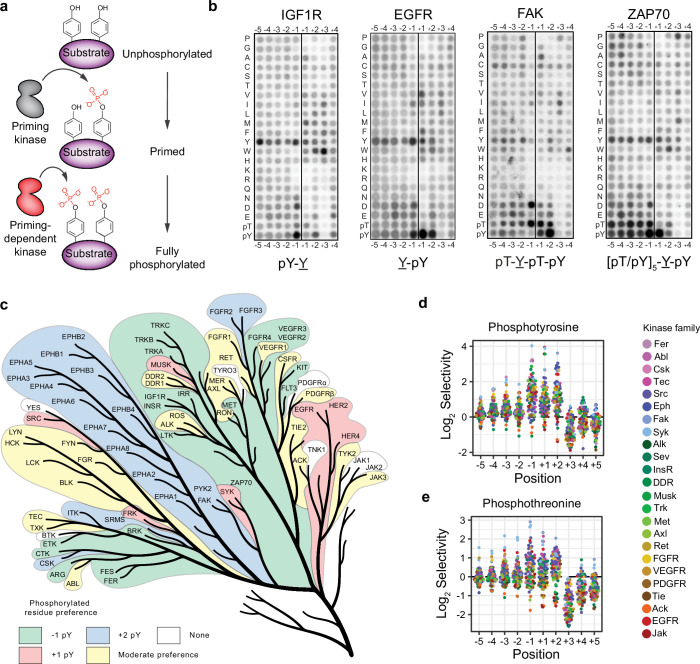
Phosphopriming favorability is a general feature of the human Tyr kinome. **a**, Schematic of Tyr substrate phosphopriming. **b**, PSPA data and simplified sequence logos highlighting various phosphopriming preferences. The Tyr phosphoacceptors in the logos are represented as Y. **c**, Phosphopriming favorability of the Tyr kinome. The colour scheme illustrates kinases that select phosphorylated Tyr as their top preferred residue at substrate position −1 (green), +1 (pink), or +2 (blue). Kinases with moderate phosphopriming preferences (where phosphorylated residues are the top preferred residues at certain substrate positions, but not overall favorites) are highlighted in yellow. The kinase TXK, as a notable exception, selects phosphorylated Thr (at position +1) as its overall preferred residue. **d-e**, Log-selectivity of the 78 conventional RTKs and nRTKs on PSPA substrate peptides containing Tyr sites flanked by phosphotyrosine (**d**) or phosphothreonine (**e**).

**Extended Data Fig. 5:**
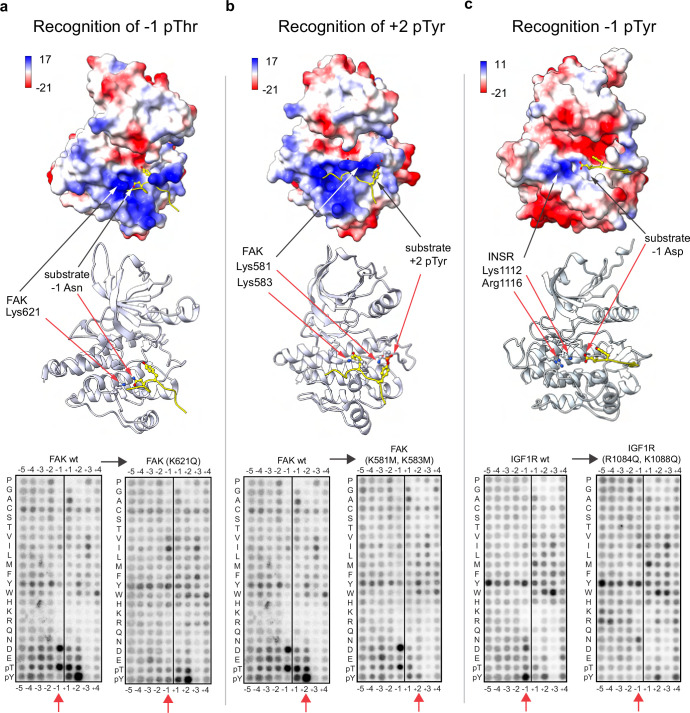
Substrate phosphopriming preferences by Tyr kinases are mediated by complementary basic residues in their catalytic domains. **a**, Top, structural modelling of the interaction between FAK and substrate peptides. Spatial alignment of FAK’s kinase domain structure (PDB: 6TY4) with the EPHB2-substrate peptide complex (PDB: 3FXX) where FAK and the substrate peptide are specifically shown to illustrate the role of Lys621 in the recognition of pThr at the −1 substrate position by FAK and, bottom, the corresponding experimental validation in PSPA assays. **b**, Top, spatial alignment of FAK’s kinase domain structure (PDB: 6TY4) with the EPHB2-substrate peptide complex (PDB: 3FXX), as performed in **a**, now illustrating the close proximity between pTyr at the +2 substrate position and Lys581 and Lys583 of FAK and, bottom, the corresponding experimental validation. **c**, Top, structural modelling of the INSR-peptide substrate complex (PDB: 1IR3), highlighting the residues in its catalytic domain that recognize pTyr at the −1 position on its substrates and, bottom, the corresponding experimental validation with its paralog IGF1R. INSR residues Lys1112 and Arg1116 are equivalent to the IGF1R residues Arg1084 and Lys1088, respectively, in their homologous alignments ^68^. Surface electrostatics are represented with Coulombic potential values were computed in ChimeraX and represented by scale bars (kcal/mol·e). Amino acid sidechains of Tyr phosphoacceptors, residues at substrate priming positions and indicated complementary residues in kinase domain are shown in ball-and-stick representation.

**Extended Data Fig. 6:**
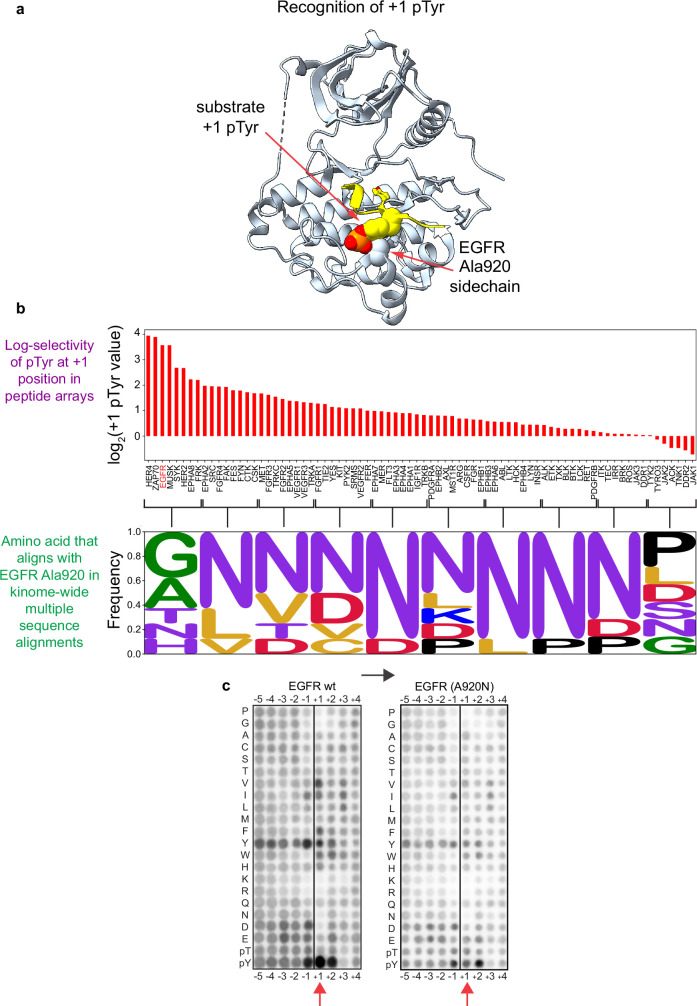
Steric accommodation of a +1 pTyr residue by EGFR. **a**, Structural modelling of EGFR’s recognition of +1 pTyr (PDB: 5CZH). Side chains of Ala920 on EGFR and +1 pTyr on the peptide substrate as shown in spacefill representation. Sidechain of Tyr phosphoacceptor is shown in ball-and-stick representation. **b**, Top, log-selectivity of the 78 conventional kinases for +1 pTyr, arranged in order of decreasing favorability. Bottom, corresponding amino acid residues that align with Ala920 of EGFR (bin size: 8 kinases in the left 8 bins; 7 kinases in the right two bins)^68^. **c**, Experimental validation of the importance of Ala920 in facilitating phosphorylation of +1 pTyr substrate by EGFR.

Phosphopriming emerged as a prominent element of biochemical specificity for many human Tyr kinases. This phenomenon, whereby a kinase recognizes an already phosphorylated residue in the substrate, can serve as a mechanism for signal integration, amplification and cross-talk. While a few Ser/Thr and Tyr kinases have been established to phosphorylate primed substrates^31,32^, we found that more than half of the conventional Tyr kinases (47 out of 78) selected a phosphorylated amino acid as their single most preferred residue across the entire peptide array (Extended Data Fig. 4) and, for over 90% of them (72 of the 78), a phosphorylated amino acid was the most favoured in at least one position. The specific patterns of phosphopriming selection were largely unique from those previously reported for Tyr kinases. For example, SYK and ZAP70 strongly preferred phosphorylated residues at several positions N-terminal to their target sites. These kinases function sequentially with other kinases in immunoreceptor signalling cascades^6,33^, and phosphopriming could help to enforce the proper order of phosphorylation for specific substrates. Position-specific selectivity for phosphorylated residues for several kinases could be rationalized based on reported kinase domain crystal structures and could be ablated by targeted mutagenesis (Extended Data Figs. 5 and 6). The biological relevance of this phosphopriming selection remains to be explored but is consistent with the abundance of multiply phosphorylated peptides observed by mass spectrometry (MS) in phosphoproteomics datasets.

## Scoring substrates for Tyr kinases

**Fig. 2:**
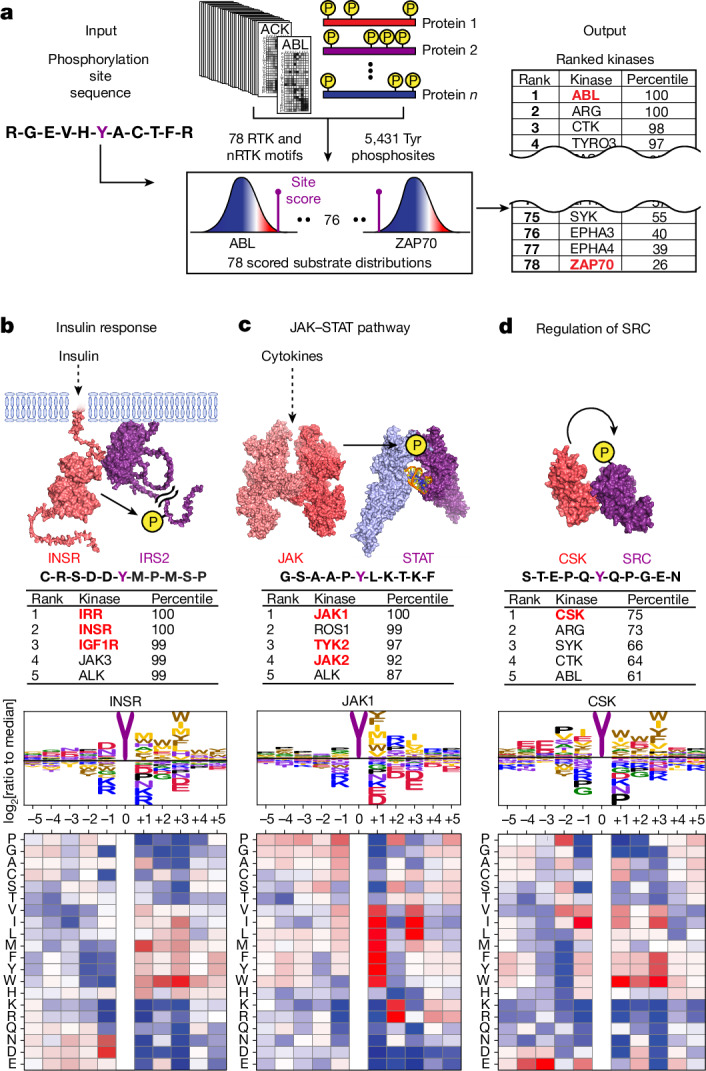
The phosphorylation motifs for the human Tyr kinome enable comparison of all kinases for Tyr phosphorylation sites. **a**, Schematic of the substrate-scoring process. **b**–**d**, Scoring results and the substrate motif logos for Tyr675 on IRS2 and the insulin receptor kinase (**b**), Tyr705 on STAT3 and JAK1 (**c**) and Tyr530 on SRC and C-terminal SRC kinase (CSK) (**d**). Red text in **b**–**d** indicates known upstream kinases.

**Extended Data Fig. 7:**
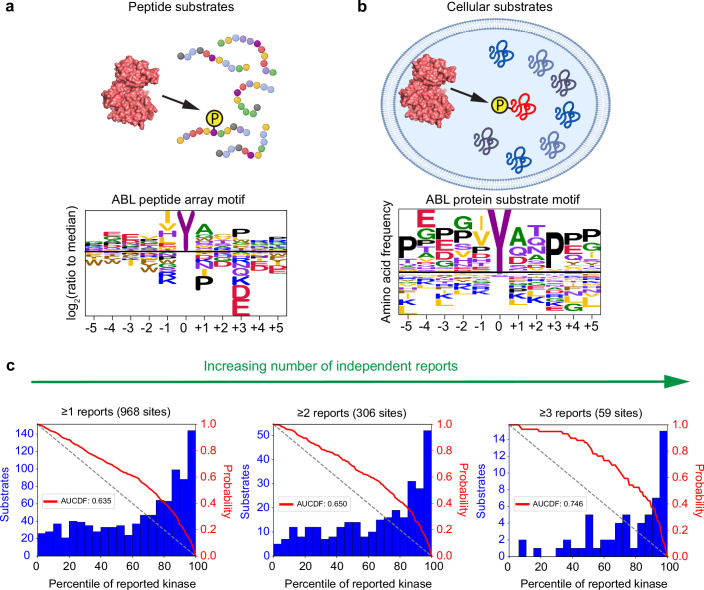
Correspondence between Tyr kinase motif-based predictions and their literature-annotated substrates. **a,b**, Schema and phosphorylation site motif logos for ABL derived from PSPA experiments (**a**) and literature-annotated cellular substrates^2^ (**b**). **c**, Percentile-score distributions of substrates for their literature-annotated kinases^2^. Higher number of reports correlates with more favourable percentile-scores between the reported kinase and its substrate (AUCDF = area under the cumulative distribution function). The diagrams in **a** and **b** were created using BioRender.

For the well-studied Tyr kinase ABL, we compared its motif specificity as identified in our peptide arrays with the amino acid sequences surrounding the mapped sites of phosphorylation on its cellular substrates^2^. The ABL PSPA (Extended Data Fig. 7a) showed a preference for aliphatic residues at −1, alanine at +1 and proline at the +3 positions, all of which were recapitulated in established ABL substrates (Extended Data Fig. 7b). We then broadened our analysis to the entire human Tyr kinome. Using a previously described bioinformatic approach^20,34,35^, position-specific scoring matrices (PSSMs) of normalized PSPA data for all conventional Tyr kinases were used to score a curated set of 5,431 sites in the human Tyr phosphoproteome^3^ plus an additional set of 1,884 Tyr phosphorylation sites identified using only low-throughput approaches^2^. Subsequently, the scores were percentile-ranked for each kinase, thereby nominating kinases best able to phosphorylate each substrate (Fig. 2a and Supplementary Table 3). When we compared our predictions to kinase–substrate pairs annotated from the literature^2^, we observed that reported substrates were enriched among highly ranking sites for their corresponding kinase. This enrichment increased among kinase–substrate relationships that were independently verified in multiple studies (Extended Data Fig. 7c). Notably, this motif-based scoring approach correctly recapitulated the upstream kinases for several of the earliest and best-established kinase–substrate relationships, including those of the insulin, the JAK–STAT and SRC signalling pathways (Fig. 2b–d).

**Extended Data Fig. 8:**
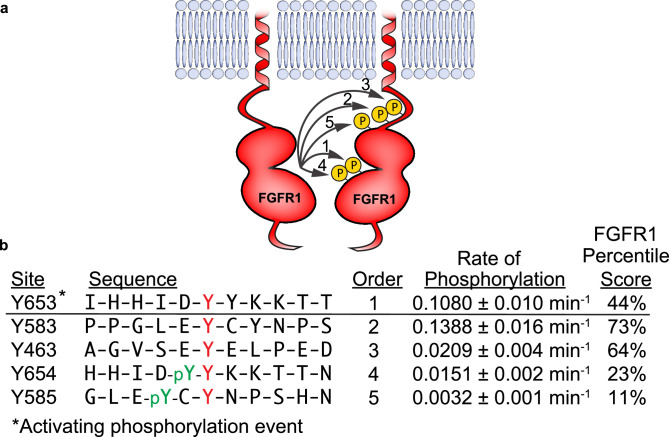
Correspondence between the order of RTK auto-trans-phosphorylation events and motif-based scores. **a**, Illustration of FGFR1 autophosphorylation. **b**, Reported rates and sequential order of auto-trans-phosphorylation of five Tyr sites on FGFR1^36^ alongside their corresponding percentile scores for FGFR1’s PSSM. Noncentral Tyr residues were treated as phosphopriming events (that is, scored as pTyr) if they preceded the central Tyr in their reported order of phosphorylation^36^. Sites of phosphorylation are indicated in red. Priming phosphorylations are indicated in green.

By contrast, autophosphorylation sites on Tyr kinases displayed a range of favourable and unfavourable motif scores as substrates of their own kinase domains, probably due to the prevalence of induced proximity. However, in such cases, these scores appeared to reflect their observed kinetics of phosphoregulation. For example, the motif scores correctly recapitulated the previously reported sequential order of FGFR autophosphorylation sites^36^ (Extended Data Fig. 8).

**Extended Data Fig. 9:**
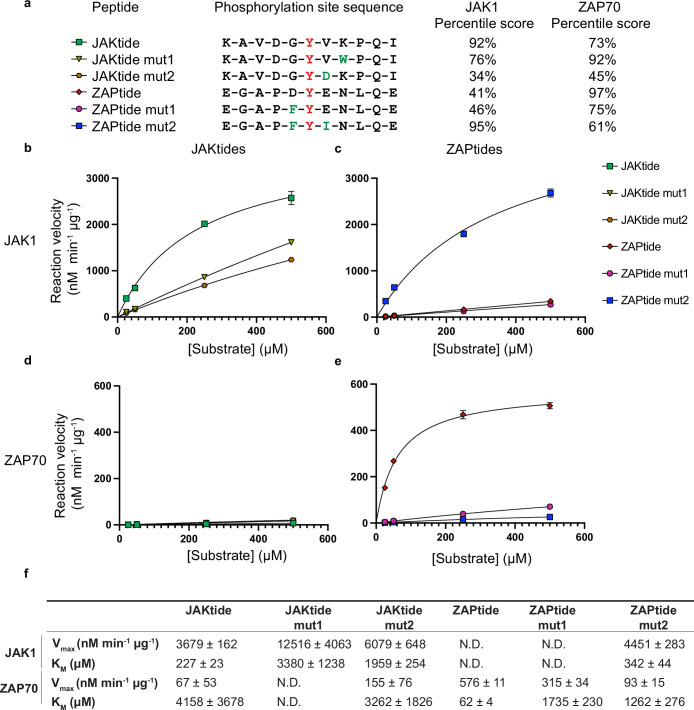
Kinetics of peptide phosphorylation by JAK1 and ZAP70. **a**, Sequences of Tyr substrate peptides. The peptides are modelled after JAK’s physiological substrate STAT5A Tyr694 (JAK-tide) and ZAP70’s physiological substrate LAT1 Tyr255 (ZAP-tide), with amino acid substitutions introduced at the indicated positions in green. Right, the corresponding percentile scores for each peptide based on the PSSMs of JAK1 and ZAP70. **b**-**c**, Kinetics of peptide phosphorylation by JAK1 on JAKtide substrates (**b**) and ZAPtide substrates (**c**). Best-fit lines illustrate fitting of the data points to Michaelis-Menten kinetics function using GraphPad Prism 10.1. Data shows mean values with error bars indicating the standard deviations of the data (n = 3 independent reactions). **d-e**, Kinetics of peptide phosphorylation by ZAP70 on JAKtide substrates (**d**) and ZAPtide substrates (**e**). Best-fit lines illustrate fitting of the data points to Michaelis-Menten kinetics function using GraphPad Prism 10.1. Data shows mean values with error bars indicating the standard deviations of the data (n = 3 independent reactions). **f**, Kinetic parameters for phosphorylation of the indicated peptides by JAK1 and ZAP70 in **b**-**e**. The standard errors of the linear fits are indicated (±). The corresponding experimental data for all these plots are presented in Supplementary Fig. 2.

Finally, to demonstrate that the effects of specific amino acid substitutions on the suitability of kinase substrates could be predicted by our PSSMs, motif-directed amino acid substitutions were made to biologically derived substrate peptides of JAK1 and ZAP70. These substitutions were capable of altering the specificity of individual substrates for their cognate kinases in predictable ways, an effect that was driven largely, but not completely, by alteration of the *K*_M_ values (Extended Data Fig. 9 and Supplementary Fig. 2).

## Tyr kinase analysis of phosphoproteomics

**Fig. 3:**
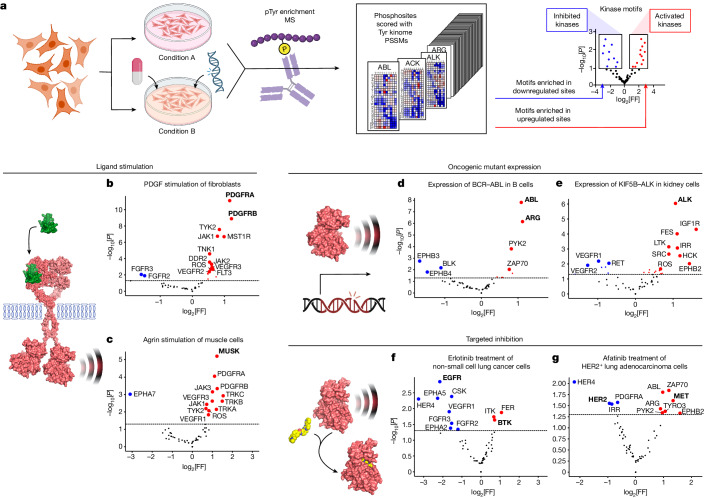
Kinome-wide motif analysis of phosphoproteomic data identifies condition-dependent patterns of kinase regulation and dysregulation. **a**, Schematic of the motif enrichment analysis of Tyr phosphoproteomics data. FF, frequency factor. **b**–**g**, Results from published datasets in cells after ligand stimulation (**b**,**c**), oncogenic mutation (**d**,**e**) or targeted inhibition (**f**,**g**) of Tyr kinases. **b**, NIH3T3 fibroblasts after 15 min treatment with 100 ng ml^−1^ PDGF-ββ^37^. **c**, Cultured myotubes after treatment for 2 h with 10 nM agrin^38^. **d**, Ba/F3 cells after expression of BCR–ABL fusion protein^41^. **e**, HEK293 cells after expression of KIF5B–ALK fusion protein^42^. **f**, PC-9 cells after treatment for 3 h with 1 μM erlotinib^44^. **g**, H1781 cells after treatment for 3 h with 1 μM afatinib^44^. Kinases indicated in bold in **b**–**g** are discussed in the main text. The enrichments in **b**–**g** were determined using one-sided exact Fisher’s tests. Fully annotated versions of these plots are shown in Supplementary Fig. 3. The diagrams in **a** and **d** were created using BioRender. Source data

This comprehensive motif collection for the Tyr kinome enables examination of phosphoproteomic MS datasets for changes in the activity level of every Tyr kinase in response to various perturbations. Using an approach similar to that previously reported for determining enrichment of Ser/Thr kinase motifs in phosphoproteomic data^20^, amino acid sequences of each phosphorylation site were scored and percentile-ranked for every human Tyr kinase (Fig. 3a). Sets of sites upregulated or downregulated in response to a given treatment were then used to infer which kinases were activated or suppressed under those conditions.

**Extended Data Fig. 10:**
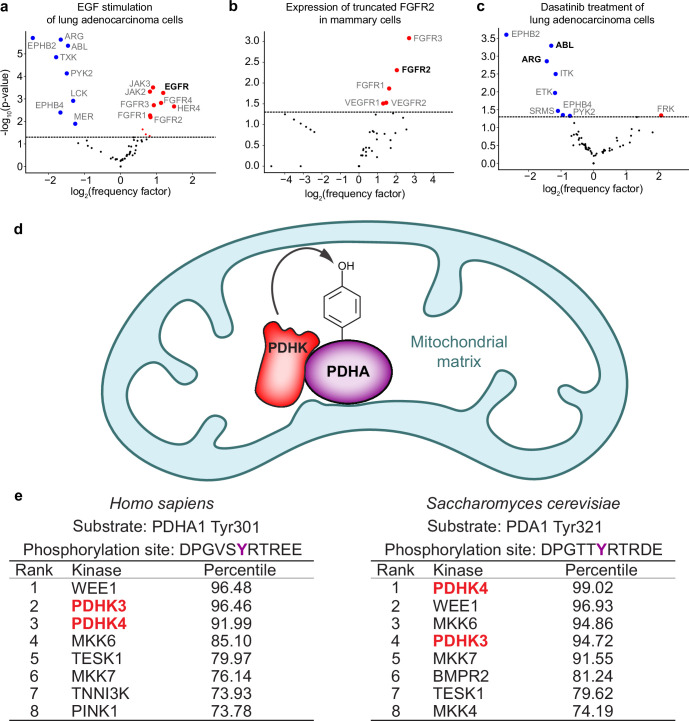
Motif-enrichment analysis of phosphoproteomics data and motif-scoring results for a suboptimal Tyr phosphorylation site on the pyruvate dehydrogenase complex. **a**, Motif-enrichment results from published datasets in cells after ligand stimulation (**a**), oncogenic mutation (**b**), or targeted inhibition (**c**) of Tyr kinases. **a**, A549 cells after 5 min treatment with 100 ng/mL EGF^40^. **b**, NMuMG cells after expression of FGFR2Δ18 mutant^43^. **c**, H2286 cells after treatment for 3 h with 1 μM dasatinib^44^. Kinases indicated in bold in **a**-**c** are discussed in the main text. The enrichments in **a**-**c** were determined using one-sided exact Fisher’s tests. **d**, Illustration of the mitochondrial-localized regulation of the pyruvate dehydrogenase complex by the PDHKs. **e**, Scoring results for human pyruvate dehydrogenase E1 component subunit alpha (PDHA1) Tyr301 and homologous site on the yeast ortholog PDA1, highlighting PDHK family members. Source data

Analysis of several published datasets using this pipeline identified specific kinases that are activated by various perturbations. For example, after acute treatment of NIH3T3 cells with PDGF^37^, the most upregulated Tyr phosphorylation motifs corresponded to those of the PDGF receptor isoforms (Fig. 3b); by contrast, in cultured myotubes stimulated with the proteoglycan agrin^38^, the most upregulated motif corresponded to its effector RTK, MuSK^39^ (Fig. 3c). Similarly, when A549 cells were stimulated with EGF^40^, the EGFR recognition motif was among the most upregulated (Extended Data Fig. 10a). In each case, the substrates driving the identification of the regulated kinase motif included both known kinase substrates (for example, PDGFRβ Tyr857 autophosphorylation, MuSK phosphorylation of acetylcholine receptor subunit β Tyr390 and EGFR phosphorylation of SHC Tyr349) and new putative substrates that conform to the same motif but were not previously described (Supplementary Table 4). These newly identified substrates both match the kinase motif and are regulated when the kinase is perturbed, lending confidence that they are likely to be directly phosphorylated by the kinase of interest. When we used this approach to analyse the phosphoproteome of cells expressing the oncogenic mutant kinases BCR–ABL^41^ or KIF5B–ALK^42^ fusion proteins or the FGFR2 variant (FGFR2(Δ18))^43^, we saw clear enrichment for the kinase motifs of each of these oncoproteins (Fig. 3d,e and Extended Data Fig. 10b). These observations suggest that motif-based analysis can identify the Tyr kinases that are most likely to be driving oncogenic events in cancer cell lines.

Finally, the atlas of Tyr kinase motifs was used to analyse recently published phosphoproteomics data on lung cancer cell lines treated with targeted inhibitors^44^. This approach identified the target kinases as well as adaptive signalling responses reported to be induced after drug treatment. For example, the ABL/SRC inhibitor dasatinib^45^ caused downregulation of the ABL phosphorylation site motif (Extended Data Fig. 10c). Treatment of a different cell line with the EGFR inhibitor erlotinib resulted in the downregulation of sites matching the EGFR motif, as well as upregulation of sites preferred by BTK, a kinase that has a role in resistance against EGFR inhibitors in that cell line^46^ (Fig. 3f). Similarly, treatment of HER2^+^ lung adenocarcinoma cells with the selective inhibitor afatinib resulted in the downregulation of the HER2 motif and upregulation of the motif of MET (Fig. 3g), a Tyr kinase that has been implicated in afatinib resistance^47,48^. These results show that the comprehensive collection of phosphorylation site motifs is sufficient to identify kinases of which the activities are either directly or indirectly targeted by a specific drug.

## Three classes of Tyr phosphosites

**Fig. 4:**
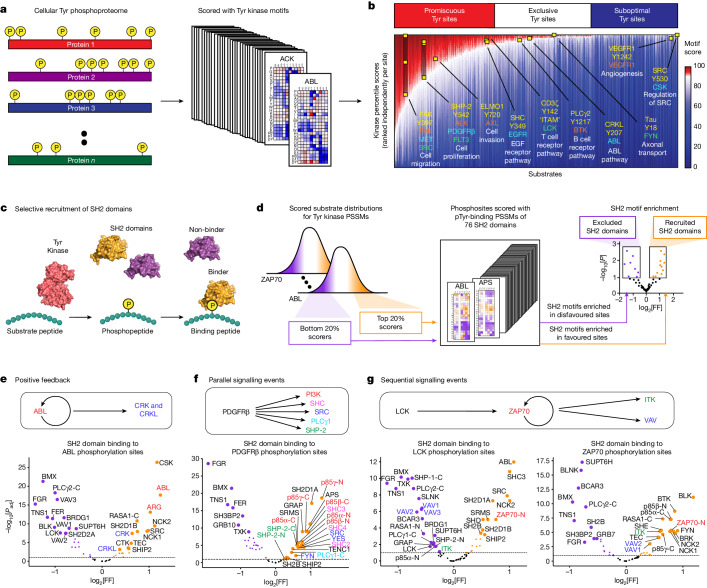
Phosphorylation motifs for the human Tyr kinome enable broad categorization of phosphosites and reveal functional correspondence with the SH2-ome. **a**, Comprehensive scoring of the Tyr phosphoproteome by all Tyr kinase motifs. **b**, Annotation of the human Tyr phosphoproteome by percentile scores with all RTK and nRTK motifs. 7,315 known human phosphorylation sites^2,3^ were sorted along the *x* axis according to the number of kinases that score the site in the 90th or higher percentile of substrates for that kinase. Independently in each column of the heat map, kinases were ranked by score for that substrate. Examples of experimentally studied kinase–substrate relationships are highlighted (yellow squares). ITAM, immunoreceptor tyrosine-based activation motif. **c**, The overlap between phosphorylation motifs of kinases and pTyr recognition motifs of SH2 domains. **d**, Schematic of the calculation of enrichment of kinase phosphorylation and SH2-domain-binding motifs. **e–g**, Signalling schematics (top) and motif enrichment plots (bottom) of SH2-binding PSSMs^53^ for Tyr phosphorylation sites scored according to the kinase PSSMs of ABL (**e**), PDGFRβ (**f**), LCK (**g**) and ZAP70 (**g**). In the schematics, the arrows represent recruitment of the indicated protein’s SH2 domain by the indicated kinases. The enrichments in **e**–**g** were determined using one-sided exact Fisher’s tests and corrected for multiple hypotheses using the Benjamini–Hochberg method. Fully annotated versions of these plots are presented in Supplementary Fig. 4. The diagrams in **c** and **d** were created using BioRender.

Annotation of the known human Tyr phosphoproteome^2,3^, based on percentile scores for the human Tyr kinome, revealed three general categories of substrates (Fig. 4a,b and Supplementary Table 3). One category, encompassing about one-third (36%) of all phosphorylation sites, scored in the 90th percentile or better for six or more conventional Tyr kinases, indicating predicted favourability to a broad spectrum of kinases. These include phosphorylation events previously known to be generated by a number of different upstream kinases and on proteins recognized by a number of SH2 domains, constituting points of convergence in signalling networks. A second category, comprising about another third (34%) of reported phosphorylation sites, instead closely matched the optimal motifs of only one to five conventional Tyr kinases, indicating substantial exclusivity in kinase–substrate relationships. Examples of phosphorylation sites in this exclusive category included carefully orchestrated regulatory events in immune cells as well as canonical kinase-specific phosphorylations. Finally, nearly one-third (31%) of all mapped Tyr phosphorylation sites poorly matched the optimal motifs of every conventional Tyr kinase. This is in sharp contrast to the Ser/Thr phosphoproteome, in which 99% of sites are well matched to at least one Ser/Thr kinase^20^. Among this class of substrates are the C-terminal phosphorylation sites of SRC-family kinases. Phosphorylation at these sites involves a docking surface with the upstream kinase CSK, which presumably overrides the requirement for an optimal phosphorylation site sequence^49^. Nonetheless, the sequence around the phosphorylation site is a better match for the CSK phosphorylation motif than that of any other conventional Tyr kinase (Fig. 2d). Notably, a subset of the suboptimal sites were in the 90th percentile of favourability for one or more of the 15 non-canonical Tyr kinases^20^ (clusters 14 and 15 in Fig. 1b and Supplementary Table 3). For example, the known regulatory site Tyr301 on the mitochondrial pyruvate dehydrogenase complex E1 alpha subunit PDHA has been repeatedly observed to be phosphorylated in cells, but its cognate kinase has not been identified^2,50^. This substrate is predicted to be a suitable match for isoforms of PDHK (Extended Data Fig. 10d,e), which are canonically believed to be Ser/Thr kinases, but for which our data demonstrate Tyr kinase activity (Fig. 1b and Supplementary Fig. 1). Notably, this Tyr site on PDHA, along with the presence of the kinase PDHK, is conserved in *Saccharomyces cerevisiae*, an organism that predates the evolutionary emergence of Tyr-exclusive kinases.

## Motif overlap with pTyr-binding proteins

Tyr kinase signalling networks frequently involve the recruitment of multiprotein complexes through modular domains that recognize and bind to the amino acid sequence surrounding a central pTyr residue^17,18^. Overlap between kinase phosphorylation site motifs and phosphotyrosine-binding adaptor proteins can provide insights into the organization of these signalling pathways^51^. As an example, SH2 domains comprise a large group of adaptor proteins that are selective for amino acids C-terminal to their pTyr sites and display great diversity in their binding motifs^7,52^. We systematically examined the relationships between our compendium of Tyr kinase motifs and a previously published collection of SH2-domain motif specificities^53^ (Fig. 4c,d and Supplementary Table 5). The overlaps between Tyr kinase and SH2 specificities identify known downstream effectors, explain positive-feedback loops and rationalize the sequential information flow of phosphorylation cascades^54,55^ (Fig. 4e–g).

## Evolution conserves kinase specificity

**Fig. 5:**
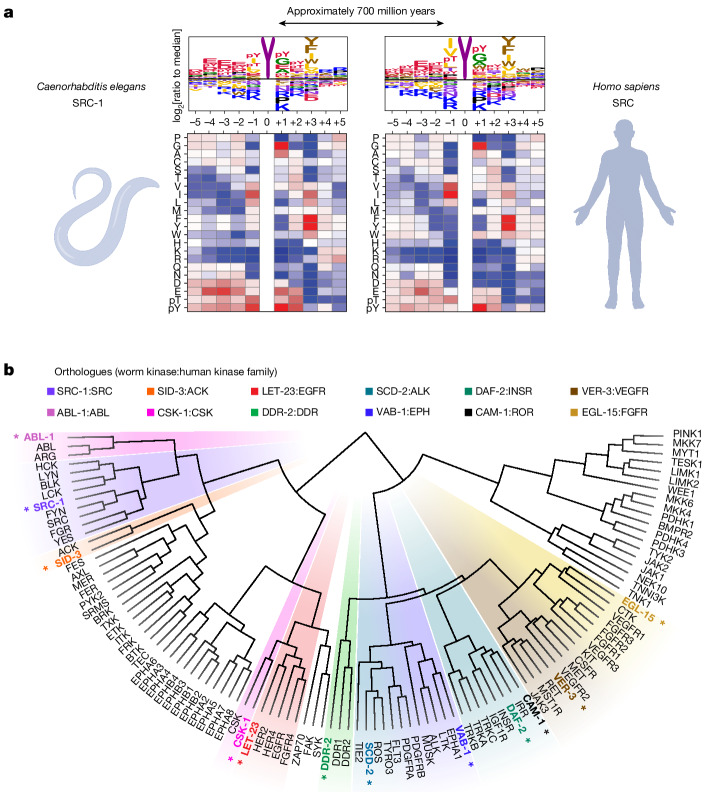
The diversity of intrinsic Tyr kinase substrate specificity is evolutionarily conserved. **a**, Comparison of sequence selectivity between the human and *C. elegans* orthologues of SRC kinase. **b**, Hierarchical clustering of the substrate motifs (PSSMs) of the human and nematode Tyr kinases. Worm kinase names are denoted with asterisks and colour coded according to their phylogenetic relationships with human Tyr kinase families (inset). Clusters containing distinct orthologous groups are highlighted. The diagram in **a** was created using BioRender.

The biological functions of several Tyr kinases are reportedly conserved throughout the animal kingdom^56^, suggesting maintenance of at least a subset of their downstream signalling pathways. Twelve kinases from the worm species *Caenorhabditis elegans*, selected as orthologues of disparate major phylogenetic branches of the human Tyr kinome, were profiled with PSPAs and their target motifs compared to those of the corresponding human kinases. In nearly all cases, the biochemical specificity of the nematode kinases appeared similar to that of their human counterparts (Fig. 5a, Supplementary Fig. 1 and Supplementary Table 6), despite hundreds of millions of years of evolutionary divergence. Hierarchical clustering of the human and nematode Tyr kinase substrate motifs reorganized the kinome into orthologous groups in which most of the human and nematode orthologues were closest neighbours (Fig. 5b), reflecting evolutionary conservation of the features that distinguish the phosphorylation-site specificities of the Tyr kinase subgroups. This strong conservation of kinase specificity across the animal kingdom probably reflects the necessity of preserving specific roles for kinases and substrate sequences that cannot be independently evolved while maintaining organismal fitness^57^.

## Discussion

Here we describe the amino acid sequence specificity for the complete set of human Tyr kinases. The various catalytic domains in the human Tyr kinome exhibit distinct substrate specificities, albeit to a lesser degree than that seen among Ser/Thr kinases. This difference is probably a consequence of the more recent divergence of Tyr kinases from the ancestral Ser/Thr kinases^58^, which have existed since before the separation of bacteria, archaea and eukaryotes. In addition to the 78 canonical human RTK and nRTKs^21^, previous research by us and others revealed 15 atypical kinases that are phylogenetically classified among the Ser/Thr kinases but that also have Tyr phosphorylation activity^20,22–25^. Here we show that these atypical kinases have motif specificities that cluster separately from those of the canonical Tyr kinases, reflecting their divergent evolutionary origin as Ser/Thr kinases.

The comprehensive nature of this collection of motif specificities enables any Tyr site to be assessed for its suitability as a substrate of each Tyr kinase, facilitating predictions as to which kinase or kinases might directly phosphorylate it. These predictions correctly identify known substrates of kinases, nominate new putative substrates and identify the kinases perturbed in a variety of phosphoproteomic MS experiments. However, we caution against overinterpreting the single top-scored or top-ranked kinases generated in these analyses. Motif-based predictions such as these are most reliable for identifying subsets of compatible kinases (frequently, phylogenetically related kinases). Other contributing factors such as tissue specificity and subcellular localization may determine which specific kinase is directly responsible for phosphorylating a given site^59^. Nonetheless, these predictions are effective at identifying individual kinases when applied in aggregate to large datasets, presumably by the accumulated evidence of many putative sites. As with Ser/Thr-kinase-motif-based predictions, our computational approaches do not consider the contributions of interpositional contacts within the substrate peptides^20^, and incorporating such information is likely to further improve predictions^59^.

Notably, over 30% of the mapped human Tyr phosphoproteome comprises sites that are poorly matched by the optimal motif specificity of canonical Tyr kinases. These sites cannot be uniformly explained by high protein abundance, reduced site stoichiometry, low evolutionary conservation, disease association, autophosphorylation, suitability as a noncanonical kinase substrate, or the presence of Ser, Thr, Tyr or Lys residues that might drive a phospho- or acetyl-mediated priming relationship. Phosphorylation of such suboptimal substrate sequences may require induced proximity, such as RTK dimerization or SH2-domain–pTyr interactions^59^.

Our characterization of phosphopriming selection by Tyr kinases and previously by Ser/Thr kinases^20^ provides insights into the order of phosphorylation events in which adjacent phosphoresidues are observed. We found that the majority of Tyr kinases select a phosphorylated residue, most often pTyr at the −1, +1 or +2 substrate positions, as their most preferred residue in the peptide array. Conversely, positioning a phosphoresidue three positions C-terminal (+3 position) to a Tyr residue hinders phosphorylation by most kinases as a ‘phospho-obstruction’ mechanism (Extended Data Fig. 4). Finally, several Tyr kinases select pThr and presumably pSer at positions in which Thr and Ser are disfavoured, indicating that Ser/Thr kinases have the ability to prime otherwise unfavourable Tyr sites for phosphorylation.

Relative to Tyr phosphorylation, far less is understood about the rules governing the dephosphorylation of pTyr sites in cells^60^. Determining substrate correspondence (that is, shared target sites) between specific protein Tyr phosphatases and kinases and understanding how their counter-regulatory activities collectively shape the Tyr phosphoproteome are important questions for future studies.

The complete collections of Tyr kinase motifs reported here and Ser/Thr kinase motifs reported previously^20^ enable one to infer kinases of which the activity changes in comparative phosphoproteomics datasets. Given the increasing abundance of such datasets, including those of individual human samples, this compendium of kinase specificities should facilitate the development of personalized therapies in the clinic.

## Methods

### Plasmids

For expression and purification from bacteria, DNA sequences for the human Tyr kinases His_6_–PKMYT1 (full length), BMPR2–His_6_ (amino acids 172–504)^20^, His_6_-TESK1 (amino acids 1–345) and the *C. elegans* Tyr kinase His_6_–ABL1 (amino acids 297–584) were codon-optimized for *Escherichia* *coli* expression using the GeneSmart prediction software (Genscript). Optimized coding sequences were synthesized as gBlocks (Integrated DNA Technologies) carrying 16 bp overhangs at the 5′ and 3′ ends to facilitate in-fusion cloning (Clontech) into pET expression vectors (EMD Millipore).

Coding sequences for 12 *C. elegans* kinases were PCR-amplified out of a cDNA library (provided as a gift from B. Emerling and M. Hansen). PCR products for *src-1* (full length), *csk-1* (full length) and *sid-3* (amino acids 93–498) were subcloned into the pcDNA 3.4 mammalian expression vector for expression in Expi293 cells. PCR products for *daf-2* (amino acids 1234–end), *let-23* (amino acids 848–end), *egl-15* (amino acids 550–end), *cam-1* (amino acids 493–end), *ddr-2* (amino acids 407–end), *ver-3* (amino acids 788–end), *scd-2* (amino acids 930–end) and *vab-1* (amino acids 582–end) were subcloned into the pFastBac Dual baculoviral expression vector for expression in Sf9 cells.

The coding sequence for CSF1R (amino acids 539–end) was PCR-amplified out of a pTag mammalian expression vector construct (a gift from M. E. Ross, C. Wang, V. Aguiar-Pulido and S. Kholmanskikh) and subcloned into pFastbacDual.

Coding sequences for EGFR (amino acids 668–end), IGF1R (amino acids 960–end) and FAK (full length) were PCR-amplified out of constructs obtained from Addgene (82906, 98344 and 23902, respectively), and subcloned into pcDNA 3.4. Amino acid substitutions in the kinase domains were generated using the QuikChange II Site-Directed Mutagenesis kit (Agilent).

### Expression and purification from bacteria

Transformations were performed with BL21 Star cells (Thermo Fisher Scientific) unless specified otherwise. Antibiotic concentrations used were as follows: carbenicillin (100 mg l^−1^), kanamycin (50 mg l^−1^), spectinomycin (25 mg l^−1^) and chloramphenicol (25 mg l^−1^ in ethanol, prepared fresh). Transformed cells were grown in 1 l Terrific broth by shaking at 190 rpm at 37 °C until the optical density (*λ* = 600 nm) reached 0.7–0.8, at which point 1 mM IPTG was added to induce expression. The cells were then transferred to a refrigerated shaker and shaken at 220 rpm at 18 °C for 16–20 h. Cells were then centrifuged at 6,000*g*, and the pellets were snap-frozen in liquid nitrogen and stored at −80 °C.

All of the steps in the protein purification were performed at 4 °C. Cell pellets were solubilized in lysis buffer (the contents of which are described below), using a spatula to disperse, and lysed by probe sonication. The lysates were centrifuged at 20,000*g* for 1 h, and the supernatants were combined with affinity purification resin, nickel NTA (Qiagen) or glutathione Sepharose (GE Health) that had been rinsed in base buffer. The supernatant–bead slurries were agitated using a rotisserie for 30 min. Resin was washed with 1 l base buffer and eluted in 10 bed volumes of elution buffer. Eluted proteins were concentrated using the Ultra Centrifugal Filter Units (Amicon), supplemented with 1 mM DTT and 25% glycerol, and snap-frozen in liquid nitrogen and stored at −80 °C.

Standard lysis buffer was 50 mM Tris pH 8.0, 100 mM NaCl, 2 mM MgCl_2_, 2% glycerol, HALT EDTA-free phosphatase and protease inhibitor cocktail (Life technologies), 5 mM β-mercaptoethanol and 1–3 grams of lysozyme (Sigma-Aldrich). Standard base buffer was 50 mM Tris pH 8.0, 100 mM NaCl, 50 mM imidazole, 2 mM MgCl_2_ and 2% glycerol. Standard wash buffer was 50 mM Tris pH 8.0, 500 mM NaCl, 50 mM imidazole, 2 mM MgCl_2_ and 2% glycerol. Polyhistidine-tag elution buffer was 50 mM Tris pH 8.0, 100 mM NaCl, 2 mM MgCl_2_, 2% glycerol and 350 mM imidazole.

PDHK1, PDHK3 and PDHK4 were co-expressed with Gro-EL/Gro-ES protein chaperones^61,62^ and purified with the following buffers: lysis buffer (100 mM potassium phosphate pH 7.5, 10 mM l-arginine (stock pH-adjusted to 7.5), 500 mM KCl, 0.1 mM EDTA, 0.1 mM EGTA, 0.2% Triton X-100, lysozyme), wash buffer (50 mM potassium phosphate pH 7.5, 10 mM arginine, 500 mM NaCl, 0.1% Triton X-100, 2 mM MgCl_2_), and elution buffer (25 mM Tris pH 7.5, 120 mM KCl, 0.02% Tween-20, 50 mM arginine, 350 mM imidazole).

PKMYT1 was co-expressed with untagged HSP90–CDC37 complex^63^.

### Protein expression in insect cells

*Spodoptera frugiperda* (Sf9) cells (Thermo Fisher Scientific) were cultured in Grace’s Insect Cell Culture Medium containing 10% fetal bovine serum (Thermo Fisher Scientific) and shaken at 120 rpm at 27 °C in a humidified incubator. According to protocols provided in the Bac-to-Bac Baculovirus Expression System manual (Thermo Fisher Scientific), Sf9 cells underwent infection with the recombinant baculoviruses derived from the pFastbac constructs described above. At 3 days after transfection, the cells were centrifuged at 500*g* for 5 min, snap-frozen in liquid nitrogen and stored at −80 °C.

### Protein expression in mammalian cells

Expi293 cells (Thermo Fisher Scientific) were cultured in 500 ml Expi293 Expression Medium (Thermo Fisher Scientific) in 2 l spinner flasks on a magnetic stirring platform at 100 rcf at 36.8 °C under 8% CO_2_. For transfection, 500 μg of expression constructs were diluted in Opti-MEM I Reduced Serum Medium (Thermo Fisher Scientific). ExpiFectamine 293 Reagent (Thermo Fisher Scientific) was diluted with Opti-MEM separately and then combined with diluted plasmid DNA for 10 min at room temperature. The mixture was then transferred to the cells (3 × 10^6^ cells per ml) and stirred. Then, 20 h after transfection, ExpiFectamine 293 Transfection Enhancer 1 and Enhancer 2 (Thermo Fisher Scientific) were added to the cells. Then, 2 days later, the cells were centrifuged at 300*g* for 5 min, snap-frozen in liquid nitrogen and stored at −80 °C (3 days after transfection).

### Purification from insect and mammalian cells

All steps of protein purification were performed at 4 °C. Cell pellets were solubilized in lysis buffer, using a spatula to disperse, and lysed by Dounce homogenization (20 strokes). The lysates were centrifuged at 100,000*g* for 1 h and the supernatants were combined with affinity purification resin, nickel NTA (Qiagen), glutathione Sepharose (GE Health) or Anti-Flag M2 affinity gel (Sigma-Aldrich), and agitated on a rotisserie for 30 min (nickel and glutathione beads) for 1 h (anti-Flag beads). The resin was washed with 1 l base buffer and eluted in 10 bed volumes of elution buffer. For elution of Flag-tagged proteins, beads were immersed in elution buffer (0.15 μg ml^−1^ 3× Flag peptide (Sigma-Aldrich)) and agitated on rotisserie for 1 h before elution. Th eluted proteins were concentrated using Ultra Centrifugal Filter Units (Amicon), supplemented with 1 mM DTT and 25% glycerol, and snap-frozen in liquid nitrogen and stored at −80 °C. Standard lysis buffer was 50 mM Tris pH 8.0, 150 mM NaCl, 2 mM MgCl_2_, 5% glycerol, 1% Triton X-100, 5 mM β-mercaptoethanol and HALT protease inhibitors. Standard base buffer was 50 mM Tris pH 8.0, 100 mM NaCl, 2 mM MgCl_2_ and 2% glycerol. Standard wash buffer was 50 mM Tris pH 8.0, 500 mM NaCl, 2 mM MgCl_2_ and 2% glycerol. Elution buffer was 50 mM Tris pH 8.0, 100 mM NaCl, 2 mM MgCl_2_ and 2% glycerol. Glutathione (10 mM) pH 8.0 was included for GST affinity purifications. Imidazole (250 mM) was included for polyhistidine affinity purifications. 3× Flag peptide (0.15 μg ml^−1^) was included for Flag affinity purifications.

Recombinant active SRMS was a gift from D. Gurbani and K. Westover^64^.

### PSPA experiments

Each recombinant kinase was distributed across a 384-well plate, mixed with a customized Tyr peptide substrate library (Anaspec) in solution phase and 50 μM ATP (50 μCi ml^−1^ γ-^32^P-ATP, Perkin-Elmer), and incubated for 90 min. Assay conditions^63^ for each kinase are described in Supplementary Table 1. Each well contains a mixture of peptides with a centralized Tyr phospho-acceptor and one fixed amino acid in an otherwise randomized background mixture of all natural amino acids except Tyr and Cys. All 20 natural amino acids, plus two PTM residues (pThr and pTyr), were substituted into positions −5 to +5 to generate 220 unique peptide mixtures (22 amino acids × 10 fixed positions). All peptides were amidated at their C termini. N- and C-terminal flanking sequences of all peptides were G-A-[phosphorylation site sequence]-A-G-K-K(biotin)-NH_2_, where K(biotin) represents a lysine sidechain modified with an aminohexanoic acid spacer attached to biotin. After the phosphorylation reactions, peptides were spotted onto Streptavidin-conjugated membranes (Promega, V2861), where they associated through their C-terminal biotinylations. The membranes were rinsed to remove free ATP and kinase and imaged using the Typhoon FLA 7000 phosphorimager (GE). Raw data (GEL file) was quantified using ImageQuant (GE). Images of the raw data are presented in Supplementary Fig. 1. For 24 kinases, the +5 position peptides were profiled in separate experiments, and their results are shown as separate images in Supplementary Fig. 1. Dual-specificity kinases (NEK10, PINK1, BMPR2, LIMK1, LIMK2, TESK1, MYT1, MKK4, MKK6, MKK7, PDHK1, PDHK3 and PDHK4) and a subset of the canonical kinases (IRR, JAK3, MST1R (RON), TXK and VEGFR1) were profiled using a second customized Tyr peptide substrate library lacking Ser, Thr, Tyr and Cys, at randomized positions.

Together, substrate motifs were obtained from a total of 109 distinct kinases, comprising 92 human kinases, 12 *Caenorhabditis elegans* kinase orthologues, 1 arthropod *Tribolium castaneum* kinase orthologue (PINK1) and 4 phosphopriming selection mutant kinases (Extended Data Fig. 5 and 6).

### Kinetic analysis

Peptide phosphorylation assays to determine the kinetic parameters of JAK1 and ZAP70 were performed at room temperature in 20 μl containing the corresponding kinase reaction buffer (Supplementary Table 1). Each reaction contained 100 ng of kinases and 500 μM, 250 μM, 50 μM or 25 μM of biotinylated substrate peptide (Anaspec). Then, 2 μl of each reaction was transferred to 18 μl quenching buffer (500 mM EDTA pH 8.0) at 0, 3, 6, 9, 12, and 15 min. A total of 1.5 μl of quenched reaction mixtures was spotted onto Streptavidin-conjugated membranes (Promega, V2861). The membranes were rinsed to remove free ATP and kinase and imaged alongside ATP standards using the Typhoon FLA 7000 phosphorimager (GE) and quantified using ImageQuant (GE). From these kinase assays, the *K*_M_ and *V*_max_ values were determined by curve fitting using the Michaelis–Menten equation (GraphPad Prism v.10.1).

### Matrix processing

The raw spot-intensity matrices of the canonical kinases and the non-canonical kinases TNNI3K and WEE1 were column-normalized (at each position) by the sum of the 18 randomized amino acids (excluding Tyr and Cys) to yield PSSMs. The raw spot-intensity matrices of all other non-canonical kinases and the canonical kinases IRR, JAK3, MST1R (RON), TXK and VEGFR1 were normalized by the sum of the 16 randomized amino acids (excluding Ser, Thr, Tyr and Cys), corresponding to the uniquely customized peptide library that was used to profile these kinases. The cysteine row was scaled to fix its median as 1/18 for the 18 amino acid library or 1/16 for the 16 amino acid library, depending on the library used as described above. The Tyr values in each position were set to be identical to the phenylalanine value at that position. For kinases displaying dual specificity (PDHK1, PDHK4, BMPR2, LIMK2, MKK7 and PINK1), the serine and threonine values in each position were set to be the median of that position.

### Substrate scoring

For scoring substrates, the PSSM values of the corresponding amino acids in the corresponding positions were scaled by 18 or 16, depending on the library used, to calculate the selectivity of that amino acid relative to the mean randomized amino acid, which has a value of 1. These values are rounded to the nearest 10,000th and multiplied to generate a raw score for each kinase–substrate pair^20,34,35^ (Supplementary Note 1). To calculate the percentile score of a substrate for a given kinase, we first computed the a priori reference score distribution of that kinase PSSM by scoring a reference Tyr phosphoproteome comprising 5,431 identified sites with localization probability above 0.75 (ref. ^3^), using the method discussed above (Fig. 2a). The percentile score of a kinase–substrate pair is defined as the percentile ranking of the substrate within the reference score distribution for the kinase.

For scores displayed at the Kinase Library websites, we log_2_-transform and sum PSSM values such that a substrate preferred over random has a positive value and a substrate selected against has a negative value.

### Matrix clustering

The dendrograms in Figs. 1 and 5 were generated using the normalized matrices with all the unmodified amino acids excluding Tyr (which was fixed as identical to phenylalanine), as well as phosphothreonine and phosphotyrosine. Linkage matrices were computed using the SciPy package in Python (v.3.7.6), using the ‘ward’ method. The results were converted to the Newick tree format and plotted using FigTree (v.1.4.4).

### Comprehensive analysis of substrate sequence selectivity

In Extended Data Figs. 3 and 4d,e, for each of the 78 canonical human Tyr kinases, the selectivities at each position for each of the 20 natural amino acids, relative to a mixed pool of natural amino acids, were calculated as described above. These values were log-transformed and plotted in v.4.2.3 of R^65^ using v.3.4.2 of the package ggplot2^66^. As a proxy for the variability among kinases in degree of selectivity, the s.d. of log-transformed selectivity values was calculated and plotted for each amino acid at each position using the same software.

### Comparison to literature PSSMs

The log_2_-enrichment of each amino acid at each position among phosphorylated peptides versus unphosphorylated library, using the subset of the library containing only one Tyr residue, was calculated previously^7^ for each of the five kinases screened against a degenerate library. The Pearson correlation coefficient *t* of these quantifications was calculated against the log_2_ selectivity for each amino acid at each position in all 78 canonical human Tyr kinases screened here. Shown in Extended Data Fig. 1 are the correlation coefficients sorted from lowest to highest with each of the five kinases screened^7^, with the five best-matching kinase selectivities in our study explicitly labelled in each plot.

### Kinase enrichment analysis

The single phosphorylation sites (not including multiply-phosphorylated peptides) in the analysed phosphoproteomics studies were scored for each of the characterized canonical kinases (78 Tyr kinases), and their ranks in the reference phosphoproteome score distributions were determined as described above. For every non-duplicated, singly phosphorylated site, kinases that ranked within the top eight kinases for the Tyr kinases were considered to be biochemically favoured kinases for that phosphorylation site. For assessing kinase motif enrichment in phosphoproteomics datasets, we compared the percentage of phosphorylation sites for which each kinase was predicted among the upregulated/downregulated (increased/decreased, respectively) phosphorylation sites (sites with |log_2_[fold change]| equal to or greater than our log[fold change] threshold of 1), versus the percentage of biochemically favoured phosphorylation sites for that kinase within the set of unregulated (unchanged) sites in this study (sites with |log_2_[fold change]| less than our log_2_[fold change] threshold of 1). Contingency tables were corrected using Haldane correction (adding 0.5 to the cases with zero in one of the counts). Statistical significance was determined using a one-sided Fisher’s exact test. Kinases that were significant (*P* ≤ 0.05) for both upregulated and downregulated analysis were excluded from the downstream analysis. Then, for each kinase, the direction of most significant enrichment (upregulated or downregulated) was selected based on the *P* values and presented in the volcano plots.

### Sequence logos

Sequence logos were generated using the Logomaker package in Python^67^. For individual kinases, the normalized matrix was used, where the height of every letter is the ratio of its value to the median value for that position. The Tyr height in the central position (position zero) was set to the maximal height in the peripheral positions. For clustered groups of kinases, the average matrix was calculated and presented as a sequence logo as described above.

### Comparative analyses between amino acids in the kinase domains and their substrate specificities

For Extended Data Fig. 6, kinases were sorted by the +1 pTyr signal in their PSSM. For the sequence logo, kinase domains of the 78 canonical Tyr kinases were obtained from previously aligned kinase sequences^68^. The alignments to residue Ala920 in EGFR (Protein Data Bank (PDB): 5CZH) were obtained for each kinase, and the frequencies of amino acids were calculated and plotted.

### Known kinase–substrate pairs

Experimentally validated kinase–substrate relationships were obtained from PhosphoSitePlus (April 2022)^2^. The number of reports for each pair was determined by the sum of the in vivo and in vitro reports.

### Performance analysis

Experimentally validated kinase–substrate relationships were obtained from PhosphoSitePlus^2^. We selected Tyr sites on human proteins and filtered out sites with an additional phosphorylated residue within 5 amino acids or sites with reported upstream kinase not characterized in this study. The number of reports for each pair was determined by the sum of the in vivo and in vitro reports.

### SH2-binding specificity matrix processing

The raw binding matrices of 76 SH2 domains were obtained from previously published work^53^. Values of zero were replaced with the minimal value at that position. Matrices were then position-normalized by the sum of the 19 randomized amino acids (excluding cysteine), to yield PSSMs^34^. The cysteine specificity was then added and set to 1/19 to represent neutral specificity as it was not included in the original data. The PSSM for PIK3R2_C was also used to represent PIK3R3_C.

### SH2 enrichment for different kinase motifs

First, we scored the Tyr phosphoproteome^3^ with each kinase motif and, for each, divided the data into favoured sites (top 20%), neutral sites (middle 60%) and disfavoured sites (bottom 20%). SH2 enrichment was then calculated similarly to the kinase enrichment process described above. SH2-binding PSSMs^53^ (Supplementary Table 5) that ranked within the top eight SH2s were considered to be biochemically favoured SH2s for binding that phosphorylation site. For assessing SH2 motif enrichment in the Tyr phosphoproteome distribution for a given kinase, we compared the percentage of phosphorylation sites for which each SH2 PSSM was predicted among the favoured/disfavoured phosphorylation sites (top 20% and bottom 20%, respectively) versus the percentage of biochemically favoured phosphorylation sites for that SH2 within the set of neutral phosphorylation sites in this study (middle 60%). Contingency tables were corrected using Haldane correction (adding 0.5 to the cases with zero in one of the counts). Statistical significance was determined using one-sided Fisher’s exact test, and the corresponding *P* values were adjusted using the Benjamini–Hochberg procedure. Finally, for every SH2 domain, the most significant direction of enrichment (favoured or disfavoured) was selected based on the adjusted *P* value and presented in the volcano plots.

### Illustrations

Experimental schema and illustrative models were generated using BioRender (https://biorender.com/). Kinome tree images were generated and modified using Coral (http://phanstiel-lab.med.unc.edu/CORAL/)^69^. Structural illustrations were generated with ChimeraX^70^ or PYMOL^71^. Generic kinase domains in Figs. 1 and 4 and Extended Data Fig. 7: INSR (PDB: 1IRK)^72^. Kinase and substrate structures in Fig. 2: INSR (structural chimera of PDB 1IRK (ref. ^72^) and AlphaFold AF-P06213-F1 (https://alphafold.ebi.ac.uk/entry/P06213) (ref. ^73^)), IRS1 (AlphaFold: AF-P35568-F1) (https://alphafold.ebi.ac.uk/entry/P35568)^73^, JAK1 (PDB: 7T6F)^74^, STAT1 (PDB: 1BF5)^75^ and CSK–SRC complex (PDB: 3D7T)^49^. RTK in Fig. 3: EGFR transmembrane domain (PDB: 2M20)^76^ and ECD (PDB: 3NJP)^77^. Kinase–drug complex in Fig. 3: ABL–imatinib (PDB: 1IEP)^78^. Generic SH2 domain structures in Fig. 4: SRC (PDB: 1SHB)^79^. Kinase domain of DDR2 in Extended Data Fig. 2 (AlphaFold: AF-Q16832-K1A, based on https://alphafold.ebi.ac.uk/entry/Q16832)^80^.

### Reporting summary

Further information on research design is available in the Nature Portfolio Reporting Summary linked to this article.

## Online content

Any methods, additional references, Nature Portfolio reporting summaries, source data, extended data, supplementary information, acknowledgements, peer review information; details of author contributions and competing interests; and statements of data and code availability are available at 10.1038/s41586-024-07407-y.

### Supplementary information

Supplementary Information Supplementary Note 1 and Supplementary Figs. 1–4.

Reporting Summary

Supplementary Table 1 Profiling Ser/Thr kinase substrate specificity. Experimental details for obtaining and profiling the 109 recombinant Tyr kinase preparations used in this study and their corresponding assay conditions.

Supplementary Table 2 PSPA data and PSSMs. Raw densitometry values obtained from the PSPA experiments in this study and their normalized values

Supplementary Table 3 Annotation of the human Tyr phosphoproteome. A total of 7,315 experimentally identified Tyr phosphorylation sites scored by the 78 canonical Tyr kinase PSSMs (page 2, corresponding to Fig. 4b) or 93 canonical plus noncanonical Tyr kinase PSSMs (page 3). The table allows one to sort substrates by percentile scores or ranks for given kinases or by promiscuity indices (number of kinases scoring above the 90th percentile) or median percentile scores.

Supplementary Table 4 Motif enrichment analysis of cell stimulated with RTK ligands. Tyr phosphorylation sites in Fig. 3b–d that were upregulated in cells after ligand stimulation and that scored favourably, ranking within the top 8 out of 78 canonical kinases, for the PSSMs of their effector RTKs

Supplementary Table 5 SH2 binding PSPA data and PSSMs. SH2 PSSM dataset: raw densitometry values and their normalized values

Supplementary Table 6 Nematode Tyr kinase PSPA data and PSSMs. *C. elegans* Tyr kinase PSSM dataset: raw densitometry values obtained from the PSPA experiments in this study and their normalized values

### Source data

Source Data Fig. 3 and Source Data Extended Data Fig. 10

## Data Availability

The data generated (raw files in Supplementary Tables 2 and 6) and analysed in this study are provided in this paper. All plasmids generated in this study are available on request. Source data are provided with this paper.

## Code availability

We have developed two slightly different approaches to determine the most likely protein kinase to phosphorylate a given site. We encourage the reader to explore both websites (https://kinase-library.phosphosite.org and https://kinase-library.mit.edu).
