# Supplementary material for: The intrinsic substrate specificity of the human tyrosine kinome
Source: Nature. 2024 May 8;629(8014):1174–81. doi: 10.1038/s41586-024-07407-y (PMC11136658; doi:10.1038/s41586-024-07407-y)
Supplement: Supplementary file 1 — Supplementary Note 1 and Supplementary Figs. 1–4. [file 41586_2024_7407_MOESM1_ESM.pdf]

nature portfolio

https://doi.org/10.1038/s41586-024-07407-y

Supplementary information

The intrinsic substrate specificity of the human tyrosine kinome

In the format provided by the authors and unedited

Supplementary Information Guide

Supplementary information Fig. 1: Compendium of peptide phosphorylation motifs for the human Tyr kinome.
Experimental results, corresponding heatmaps, and phosphorylation site motif logos for the human and nematode tyrosine kinases profiled in this study. Heatmaps represent PSSM values normalized such that the sum of the randomized amino acids equal 1. Pages 1-2 list the kinases alphabetically by their gene name. Clicking on the name of the kinase takes you to its results page.

Supplementary information Fig. 2: Kinetics of peptide phosphorylation by JAK1 and ZAP70
Experimental data corresponding to the kinetic analyses in Extended Data Figure 9.

Supplementary information Fig. 3: Global motif analysis reveals how kinase perturbations and pathway rewiring reshape the phosphoproteome.
Fully annotated volcano plots from Figs. 3c,3e,3g and 3h.

Supplementary information Fig. 4: Phosphorylation motifs for the human Tyr kinome reveal functional correspondence with the SH2-ome.
Fully annotated volcano plots from Extended Data Figs 11c-e.

Supplementary information Note 1: Scoring substrates.
Mathematical formula for applying PSSM data to score substrate sequences.

Supplementary information Table 1: Profiling Ser/Thr kinase substrate specificity.
Experimental details for obtaining and profiling the 109 recombinant Tyr kinase preparations used in this study and their corresponding assay conditions.

Supplementary information Table 2: PSPA data and PSSMs.
Raw densitometry values obtained from the PSPA experiments in this study and their normalized values.

Supplementary information Table 3: Annotation of the human Tyr phosphoproteome.
7,315 experimentally identified Tyr phosphorylation sites scored by the 78 canonical Tyr kinase PSSMs (page 2, corresponding to Fig. 4b) or 93 canonical plus noncanonical Tyr kinase PSSMs (page 3). The table allows one to sort substrates by percentile scores or ranks for given kinases or by promiscuity indices (number of kinases scoring above the 90th percentile) or median percentile scores.

Supplementary information Table 4: Motif enrichment analysis of cell stimulated with RTK ligands.
Tyr phosphorylation sites in Figs. 3b-d that were upregulated in cells after ligand stimulation and that scored favorably, ranking within the top 8/78 canonical kinases, for the PSSMs of their effector RTKs.

Supplementary information Table 5: SH2 binding PSPA data and PSSMs.
SH2 PSSM dataset: raw densitometry values and their normalized values.

Supplementary information Table 6: Nematode Tyr kinase PSPA data and PSSMs.
C. elegans Tyr kinase PSSM dataset: raw densitometry values obtained from the PSPA experiments in this study and their normalized values.

Supplementary Figure 1:
Compendium of experimental peptide phosphorylation motifs for the human tyrosine kinome

Click name of kinase to go to results page

GENE	PROTEIN	UNIPROT ID
ABL1	ABL	P00519
ABL2	ARG	P42684
ALK	ALK	Q9UM73
AXL	AXL	P30530
BLK	BLK	P51451
BMPR2	BMPR2	Q13873
BMX	ETK	P51813
BTK	BTK	Q06187
CSF1R	CSFR	P07333
CSK	CSK	P41240
DDR1	DDR1	Q08345
DDR2	DDR2	Q16832
EGFR	EGFR	P00533
EPHA1	EPHA1	P21709
EPHA2	EPHA2	P29317
EPHA3	EPHA3	P29320
EPHA4	EPHA4	P54764
EPHA5	EPHA5	P54756
EPHA6	EPHA6	Q9UF33
EPHA7	EPHA7	Q15375
EPHA8	EPHA8	P29322
EPHB1	EPHB1	P54762
EPHB2	EPHB2	P29323
EPHB3	EPHB3	P54753
EPHB4	EPHB4	P54760

GENE	PROTEIN	UNIPROT ID
ERBB2	HER2	P04626
ERBB4	HER4	Q15303
FER	FER	P16591
FES	FES	P07332
FGFR1	FGFR1	P11362
FGFR2	FGFR2	P21802
FGFR3	FGFR3	P22607
FGFR4	FGFR4	P22455
FGR	FGR	P09769
FLT1	VEGFR1	P17948
FLT3	FLT3	P36888
FLT4	VEGFR3	P35916
FRK	FRK	P42685
FYN	FYN	P06241
HCK	HCK	P08631
IGF1R	IGF1R	P08069
INSR	INSR	P06213
IRR	IRR	P14616
ITK	ITK	Q08881
JAK1	JAK1	P23458
JAK2	JAK2	O60674
JAK3	JAK3	P52333
KDR	VEGFR2	P35968
KIT	KIT	P10721
LCK	LCK	P06239

GENE	PROTEIN	UNIPROT ID
LIMK1	LIMK1	P53667
LIMK2	LIMK2	P53671
LTK	LTK	P29376
LYN	LYN	P07948
MATK	CTK	P42679
MERTK	MER	Q12866
MET	MET	P08581
MAP2K4	MKK4	P45985
MAP2K6	MKK6	P52564
MAP2K7	MKK7	O14733
MST1R	RON	Q04912
MUSK	MUSK	O15146
NEK10	NEK10	Q6ZWH5
NTRK1	TRKA	P04629
NTRK2	TRKB	Q16620
NTRK3	TRKC	Q16288
PDGFRA	PDGFRA	P16234
PDGFRB	PDGFRB	P09619
PDK1	PDHK1	Q15118
PDK3	PDHK3	Q15120
PDK4	PDHK4	Q16654
PINK1	PINK1	D6WMX4
PKMYT1	MYT1	Q99640
PTK2	FAK	Q05397
PTK2B	PYK2	Q14289
PTK6	BRK	Q13882
RET	RET	P07949
ROS1	ROS	P08922

	GENE	PROTEIN	UNIPROT ID
	SRC	SRC	P12931
	SRMS	SRMS	Q9H3Y6
	SYK	SYK	P43405
	TEC	TEC	P42680
	TEK	TIE2	Q02763
	TESK1	TESK1	Q15569
	TNK1	TNK1	Q13470
	TNK2	ACK	Q07912
	TNNI3K	TNNI3K	Q59H18
	TXK	TXK	P42681
	TYK2	TYK2	P29597
	TYRO3	TYRO3	Q06418
	WEE1	WEE1	P30291
	YES1	YES	P07947
	ZAP70	ZAP70	P43403
C. elegans			
	abl-1	ABL1	P03949
	cam-1	CAM1	G5EGK5
	csk-1	CSK1	G5ECJ6
	daf-2	INSR	Q968Y9
	ddr-2	DDRB	Q95ZV7
	egl-15	EGL15	Q10656
	let-23	LET23	P24348
	scd-2	SCD2	O76411
	sid-3	SID3	Q10925
	src-1	SRC1	G5EE56
	vab-1	VAB1	O61460
	ver-3	VER3	Q21038

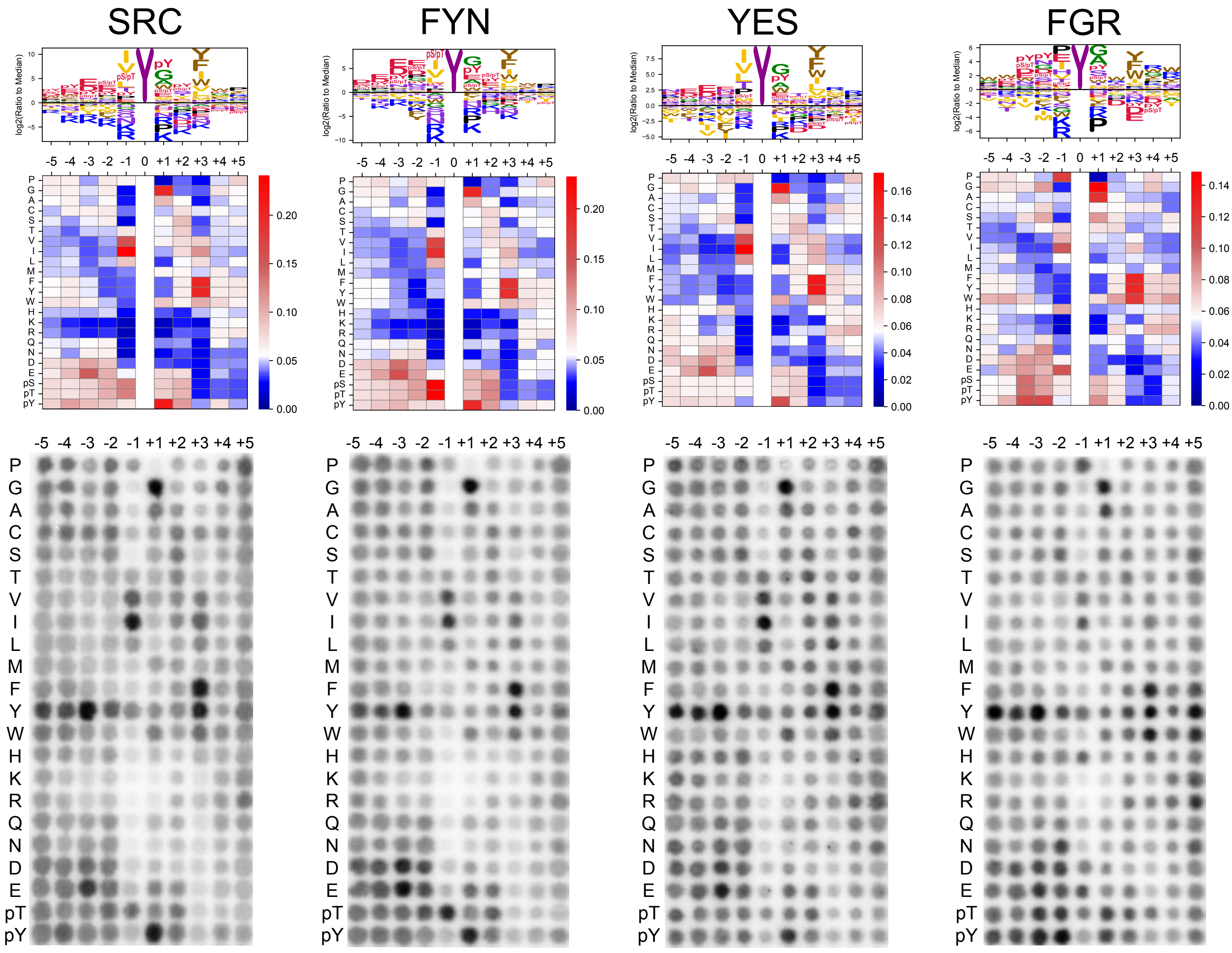
SRC
FYN
YES
FGR
log2(Ratio to Median)
-5 -4 -3 -2 -1 0 +1 +2 +3 +4 +5
P G A C S T V I L M F Y W H K R Q N D E pS pT pY
-5 -4 -3 -2 -1 +1 +2 +3 +4 +5
P G A C S T V I L M F Y W H K R Q N D E pT pY

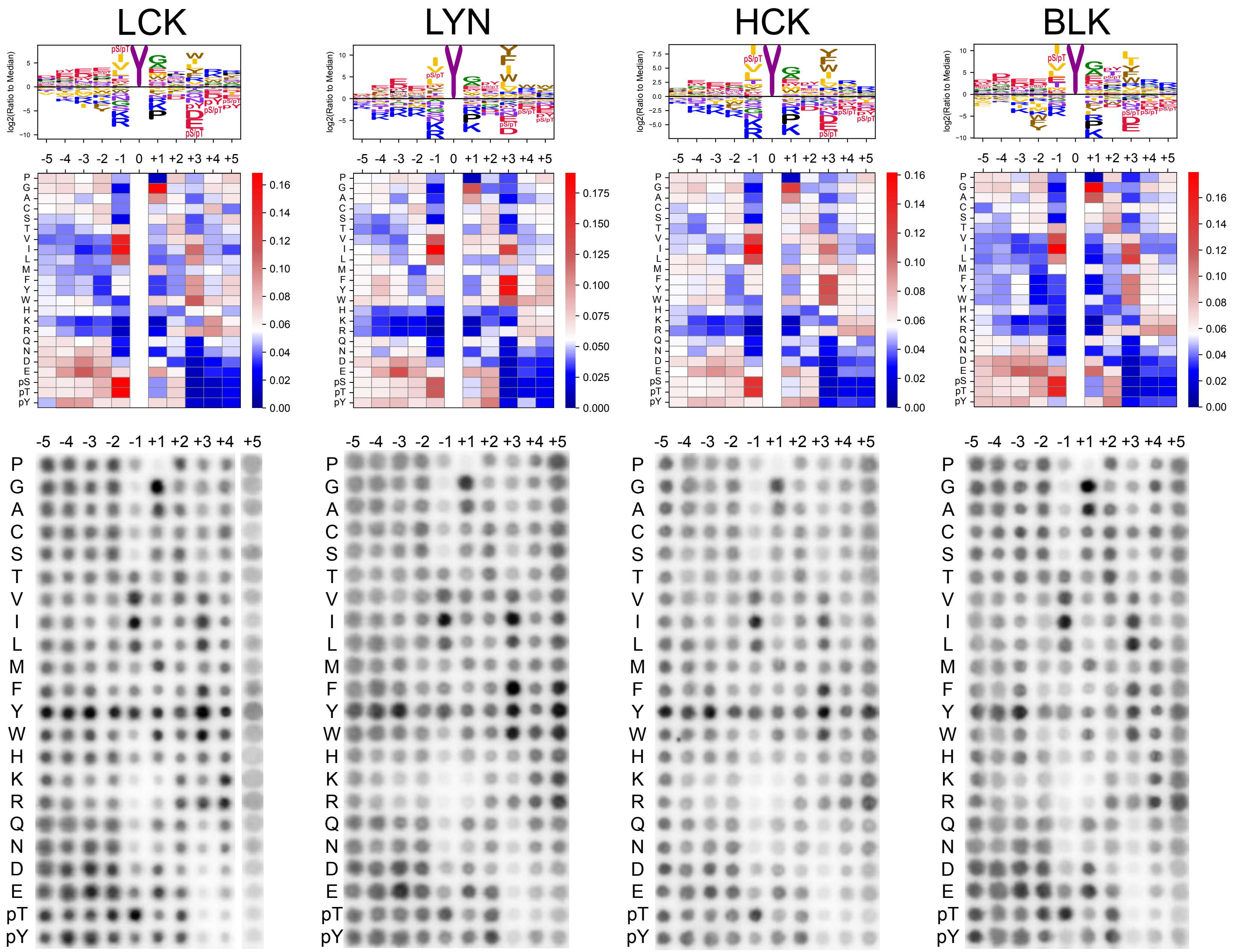
LCK
LYN
HCK
BLK
log2(Ratio to Median)
-5 -4 -3 -2 -1 0 +1 +2 +3 +4 +5
P G A C S T V I L M F Y W H K R Q N D E pS pT pY
0.16 0.14 0.12 0.10 0.08 0.06 0.04 0.02 0.00
0.175 0.150 0.125 0.100 0.075 0.050 0.025 0.000
0.16 0.14 0.12 0.10 0.08 0.06 0.04 0.02 0.00
0.16 0.14 0.12 0.10 0.08 0.06 0.04 0.02 0.00
-5 -4 -3 -2 -1 +1 +2 +3 +4 +5
P G A C S T V I L M F Y W H K R Q N D E pT pY

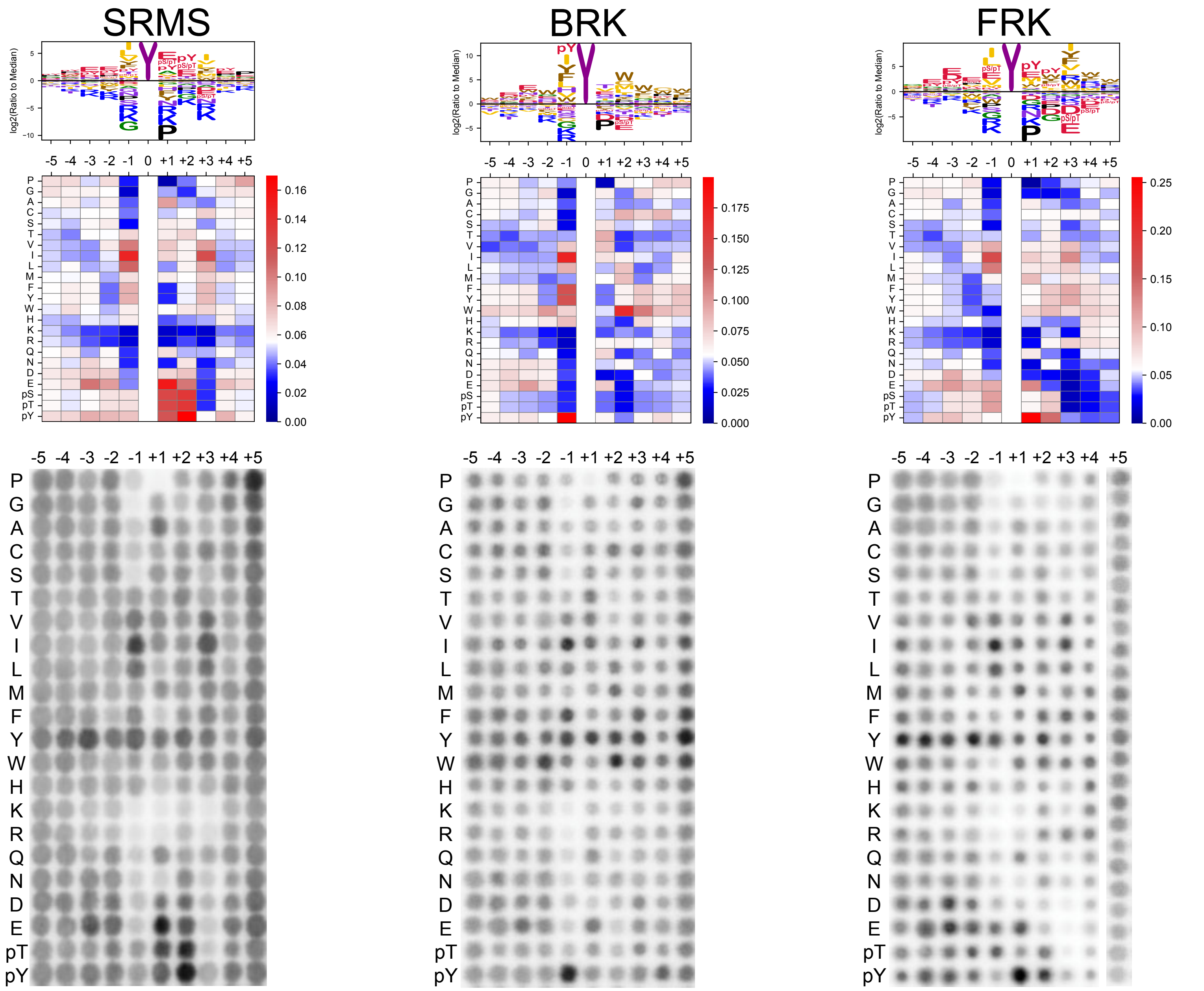

SRMS
BRK
FRK
log2(Ratio to Median)
-5 -4 -3 -2 -1 0 +1 +2 +3 +4 +5
P
G
A
C
S
T
V
I
L
M
F
Y
W
H
K
R
Q
N
D
E
pS
pT
pY
-5 -4 -3 -2 -1 +1 +2 +3 +4 +5

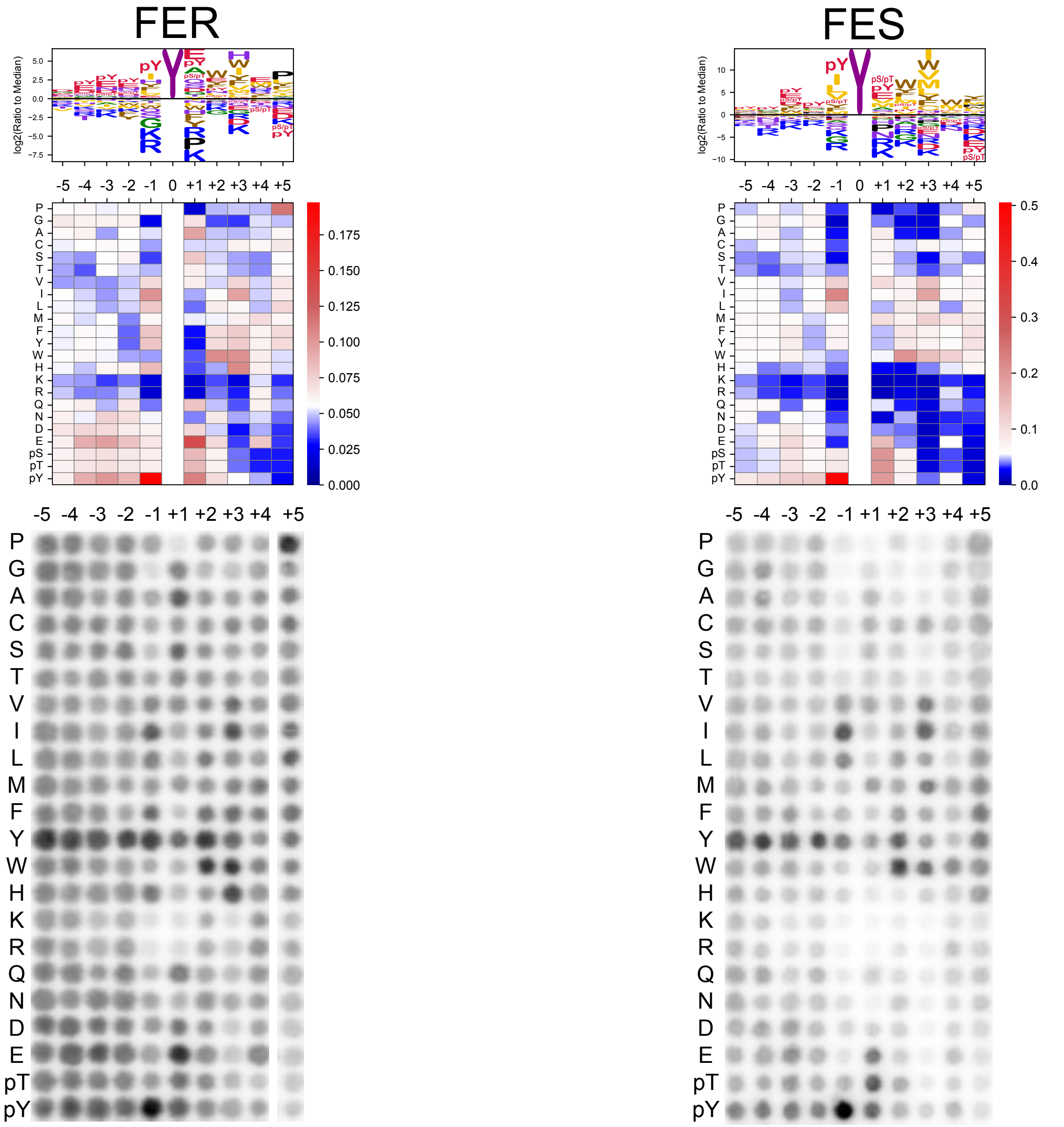
FER
FES
log2(Ratio to Median)
-5 -4 -3 -2 -1 0 +1 +2 +3 +4 +5
P G A C S T V I L M F Y W H K R Q N D E pS pT pY
0.175 0.150 0.125 0.100 0.075 0.050 0.025 0.000
0.5 0.4 0.3 0.2 0.1 0.0
-5 -4 -3 -2 -1 +1 +2 +3 +4 +5
P G A C S T V I L M F Y W H K R Q N D E pT pY

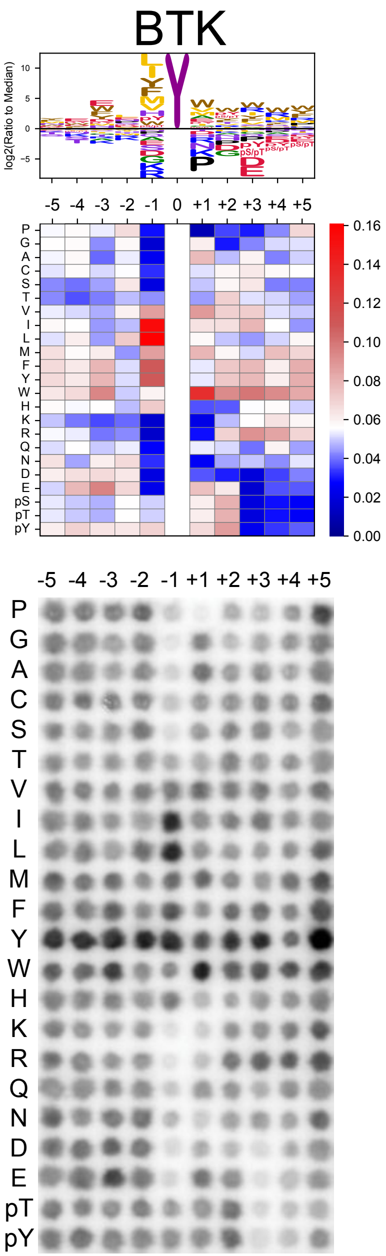
BTK
log2(Ratio to Median)
-5 -4 -3 -2 -1 0 +1 +2 +3 +4 +5
P G A C S T V I L M F Y W H K R Q N D E pS pT pY
-5 -4 -3 -2 -1 +1 +2 +3 +4 +5
P G A C S T V I L M F Y W H K R Q N D E pT pY

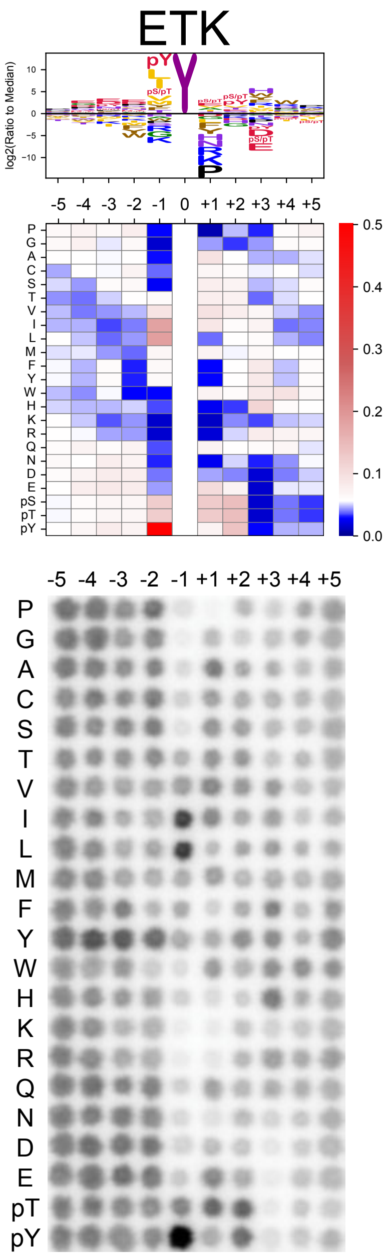
ETK
log2(Ratio to Median)
-5 -4 -3 -2 -1 0 +1 +2 +3 +4 +5
P G A C S T V I L M F Y W H K R Q N D E pS pT pY
-5 -4 -3 -2 -1 +1 +2 +3 +4 +5
P G A C S T V I L M F Y W H K R Q N D E pT pY

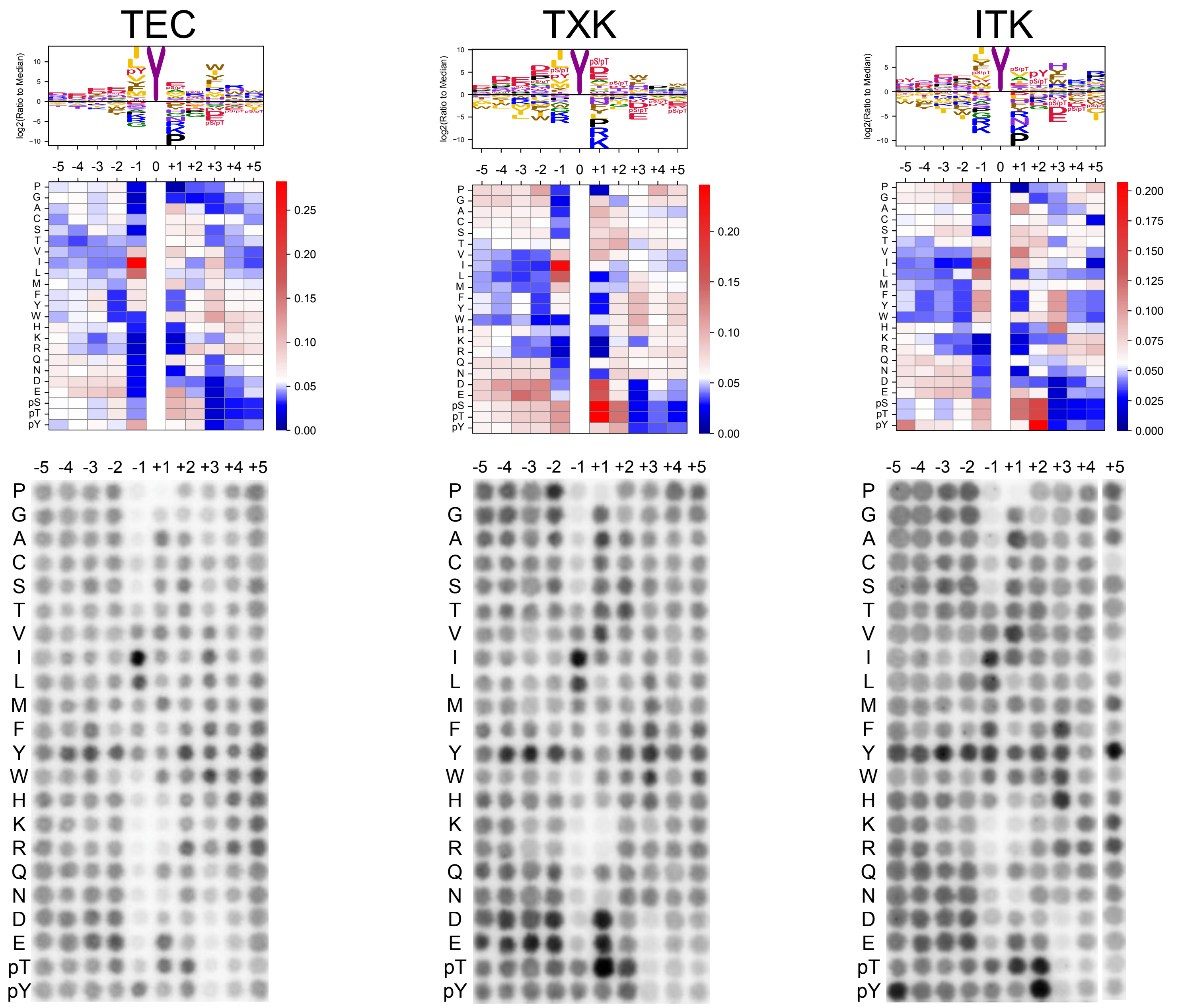
TEC
TXK
ITK
log2(Ratio to Median)
-5 -4 -3 -2 -1 0 +1 +2 +3 +4 +5
P G A C S T V I L M F Y W H K R Q N D E pS pT pY
P G A C S T V I L M F Y W H K R Q N D E pT pY

CSK

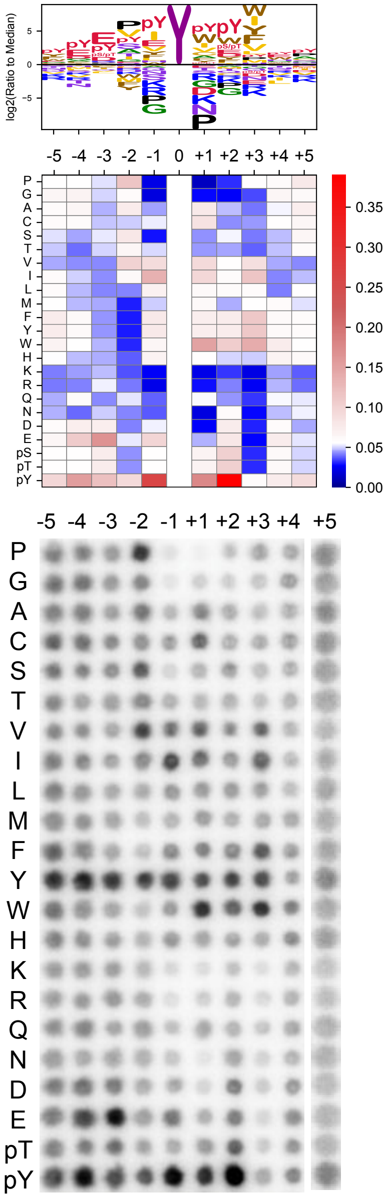
log2(Ratio to Median)
-5 -4 -3 -2 -1 0 +1 +2 +3 +4 +5
P G A C S T V I L M F Y W H K R Q N D E pS pT pY
0.35 0.30 0.25 0.20 0.15 0.10 0.05 0.00
-5 -4 -3 -2 -1 +1 +2 +3 +4 +5
P G A C S T V I L M F Y W H K R Q N D E pT pY

CTK

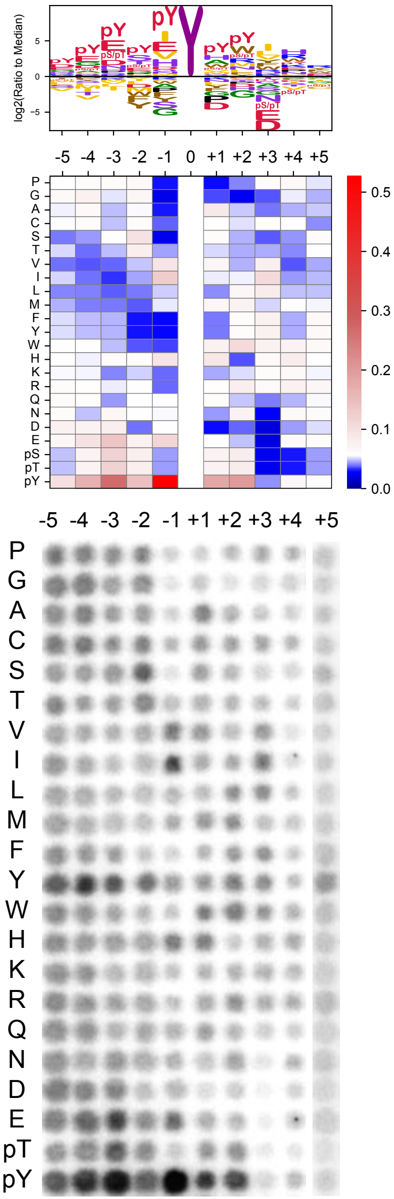
log2(Ratio to Median)
-5 -4 -3 -2 -1 0 +1 +2 +3 +4 +5
P G A C S T V I L M F Y W H K R Q N D E pS pT pY
0.5 0.4 0.3 0.2 0.1 0.0
-5 -4 -3 -2 -1 +1 +2 +3 +4 +5
P G A C S T V I L M F Y W H K R Q N D E pT pY

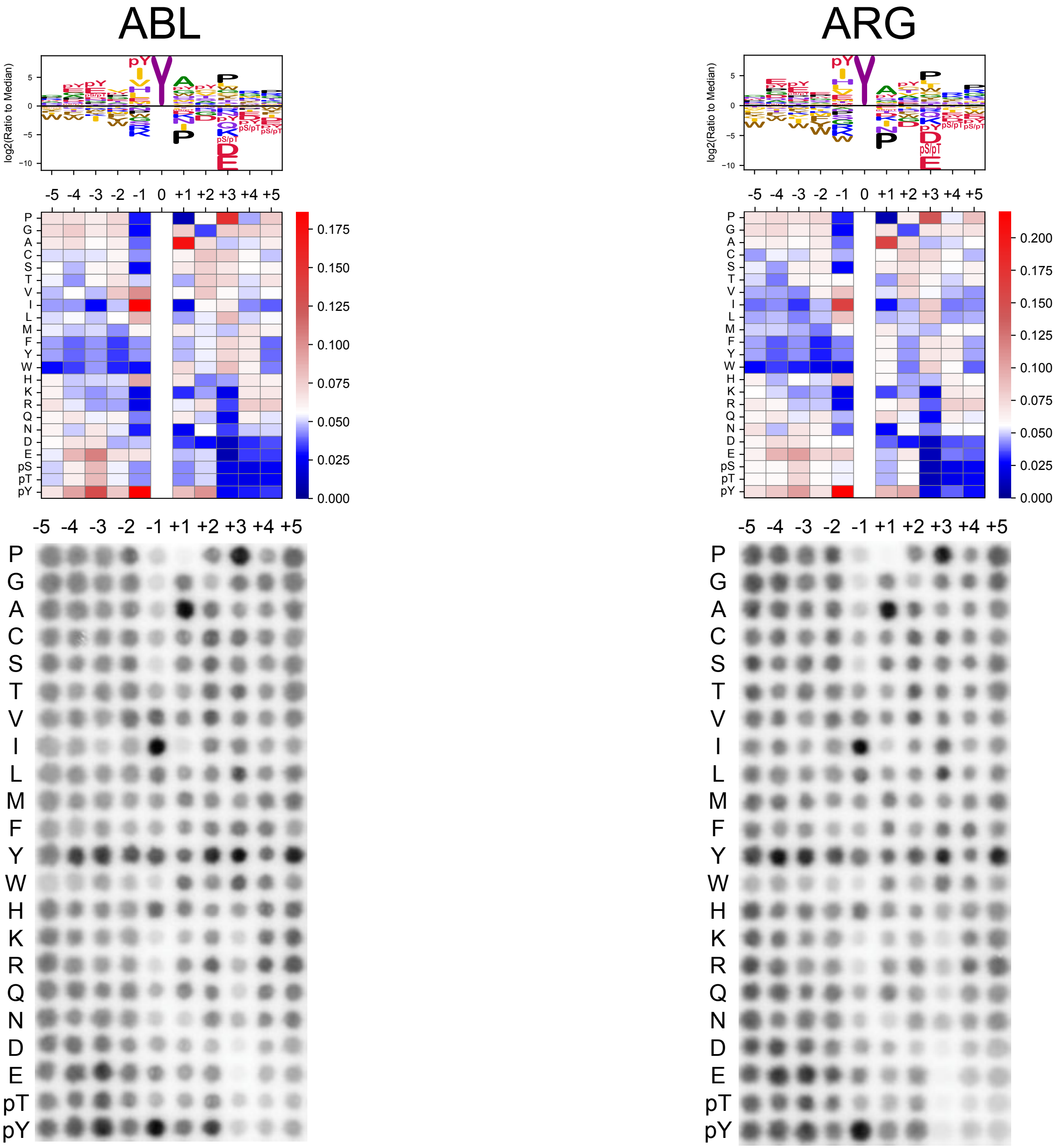
ABL
ARG
log2(Ratio to Median)
-5 -4 -3 -2 -1 0 +1 +2 +3 +4 +5
P G A C S T V I L M F Y W H K R Q N D E pS pT pY
0.175 0.150 0.125 0.100 0.075 0.050 0.025 0.000
0.200 0.175 0.150 0.125 0.100 0.075 0.050 0.025 0.000
-5 -4 -3 -2 -1 +1 +2 +3 +4 +5
P G A C S T V I L M F Y W H K R Q N D E pT pY

ACK

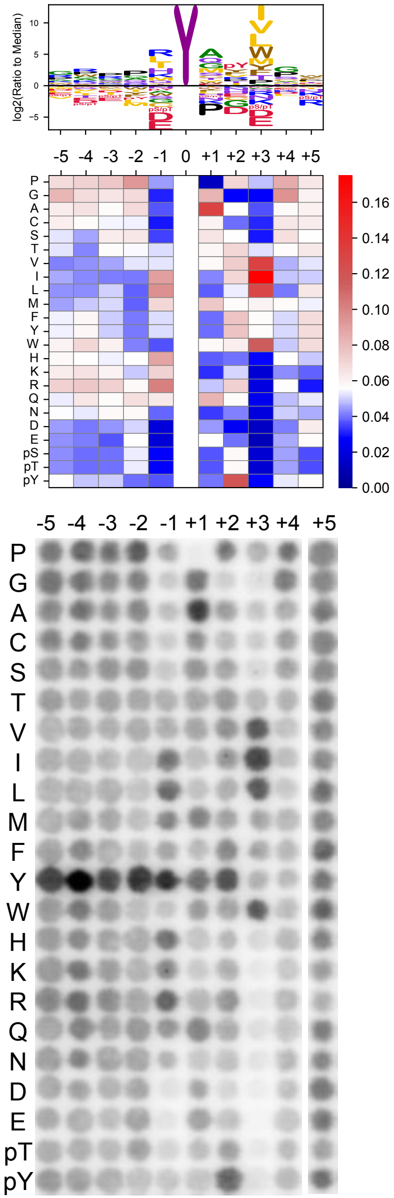

log2(Ratio to Median)
-5 -4 -3 -2 -1 0 +1 +2 +3 +4 +5
P G A C S T V I L M F Y W H K R Q N D E pS pT pY
0.16 0.14 0.12 0.10 0.08 0.06 0.04 0.02 0.00
-5 -4 -3 -2 -1 +1 +2 +3 +4 +5
P G A C S T V I L M F Y W H K R Q N D E pT pY

TNK1

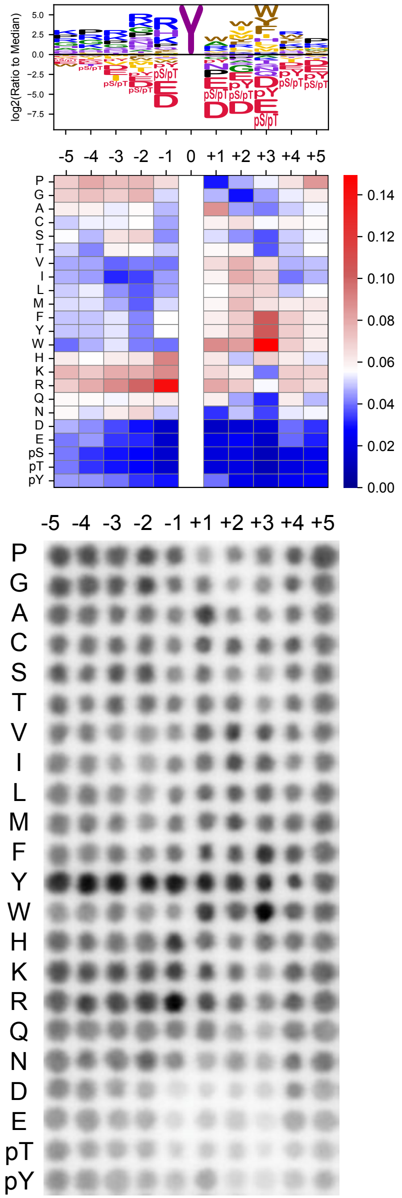

log2(Ratio to Median)
-5 -4 -3 -2 -1 0 +1 +2 +3 +4 +5
P G A C S T V I L M F Y W H K R Q N D E pS pT pY
0.14 0.12 0.10 0.08 0.06 0.04 0.02 0.00
-5 -4 -3 -2 -1 +1 +2 +3 +4 +5
P G A C S T V I L M F Y W H K R Q N D E pT pY

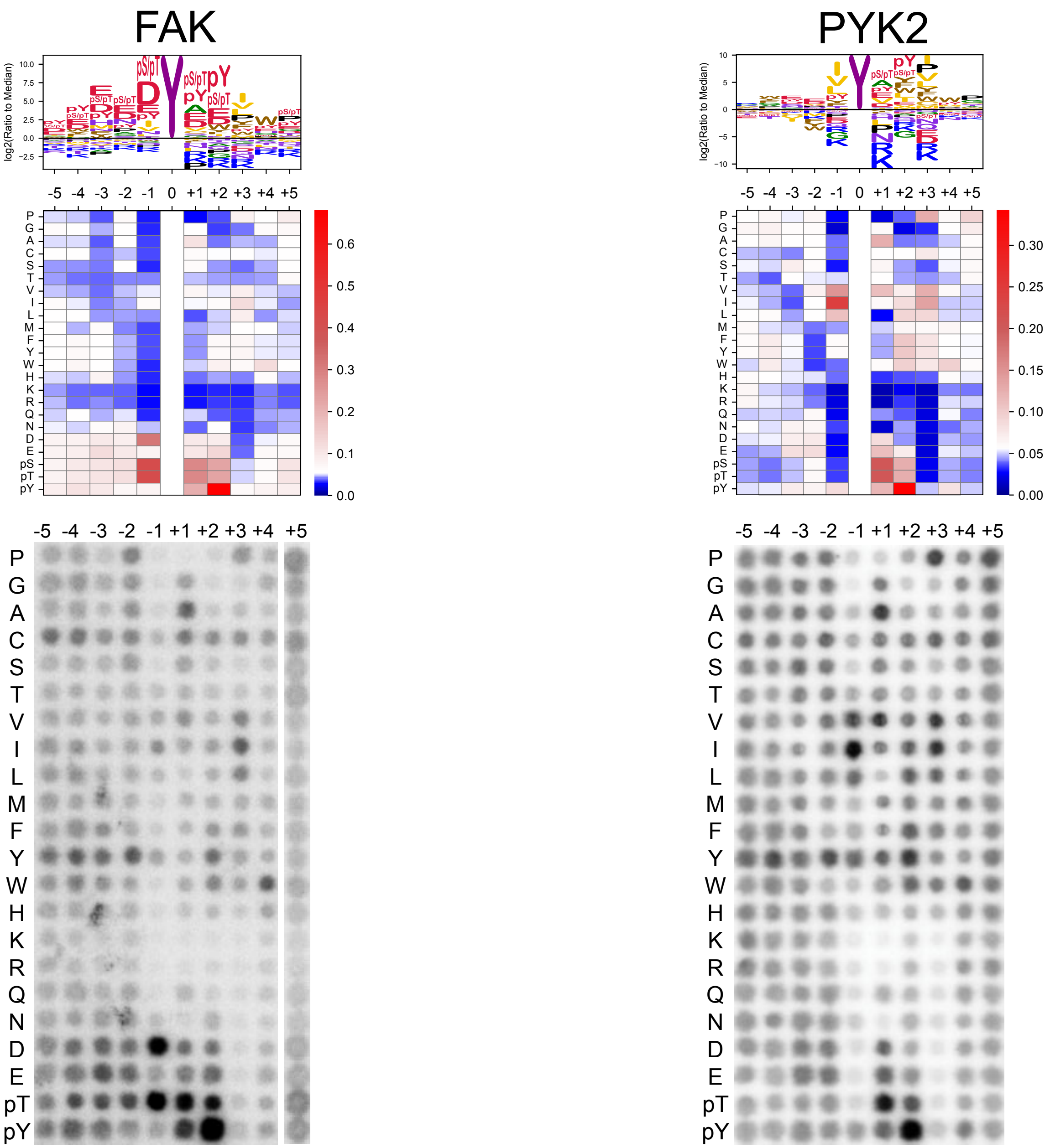
FAK
PYK2
log2(Ratio to Median)
-5 -4 -3 -2 -1 0 +1 +2 +3 +4 +5
P G A C S T V I L M F Y W H K R Q N D E pS pT pY
-5 -4 -3 -2 -1 +1 +2 +3 +4 +5
P G A C S T V I L M F Y W H K R Q N D E pT pY

SYK

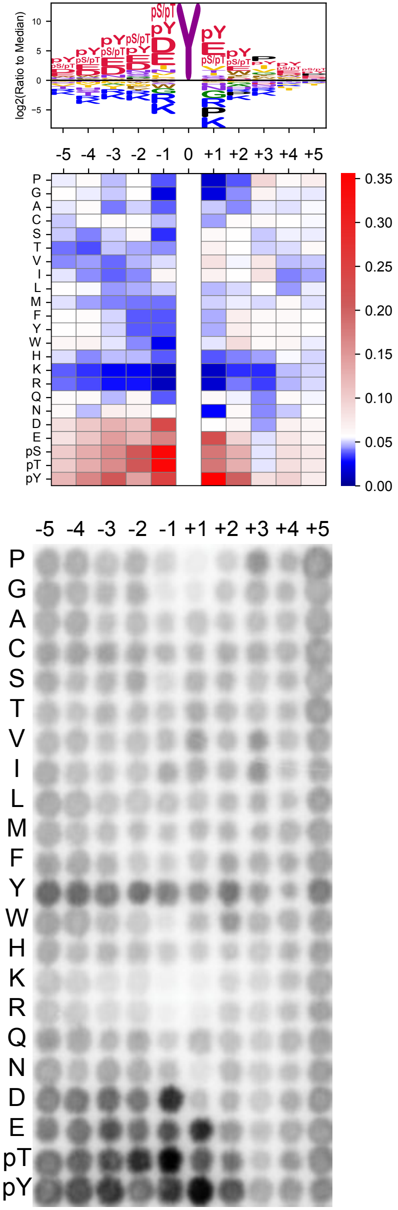
log2(Ratio to Median)
10
5
0
-5
-5 -4 -3 -2 -1 0 +1 +2 +3 +4 +5
P G A C S T V I L M F Y W H K R Q N D E pS pT pY
0.35
0.30
0.25
0.20
0.15
0.10
0.05
0.00
-5 -4 -3 -2 -1 +1 +2 +3 +4 +5
P G A C S T V I L M F Y W H K R Q N D E pT pY

ZAP70

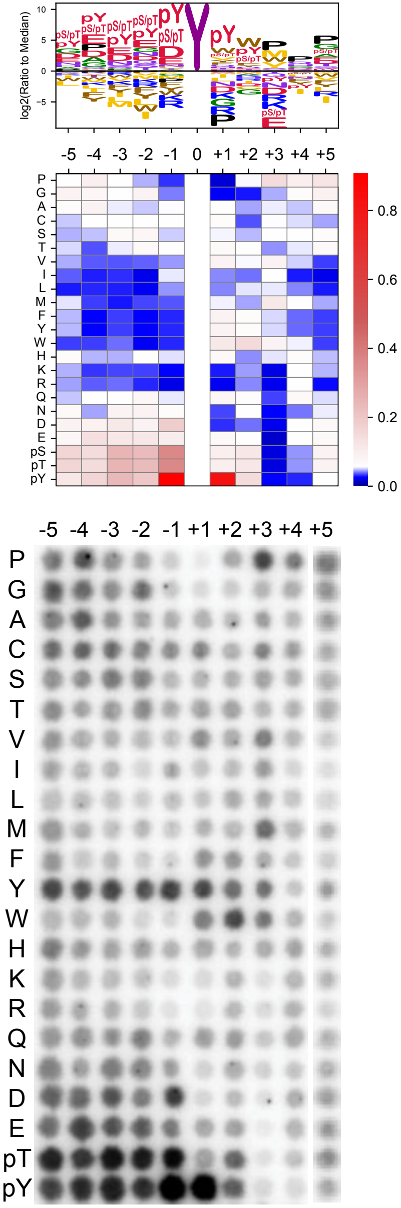
log2(Ratio to Median)
10
5
0
-5
-5 -4 -3 -2 -1 0 +1 +2 +3 +4 +5
P G A C S T V I L M F Y W H K R Q N D E pS pT pY
0.8
0.6
0.4
0.2
0.0
-5 -4 -3 -2 -1 +1 +2 +3 +4 +5
P G A C S T V I L M F Y W H K R Q N D E pT pY

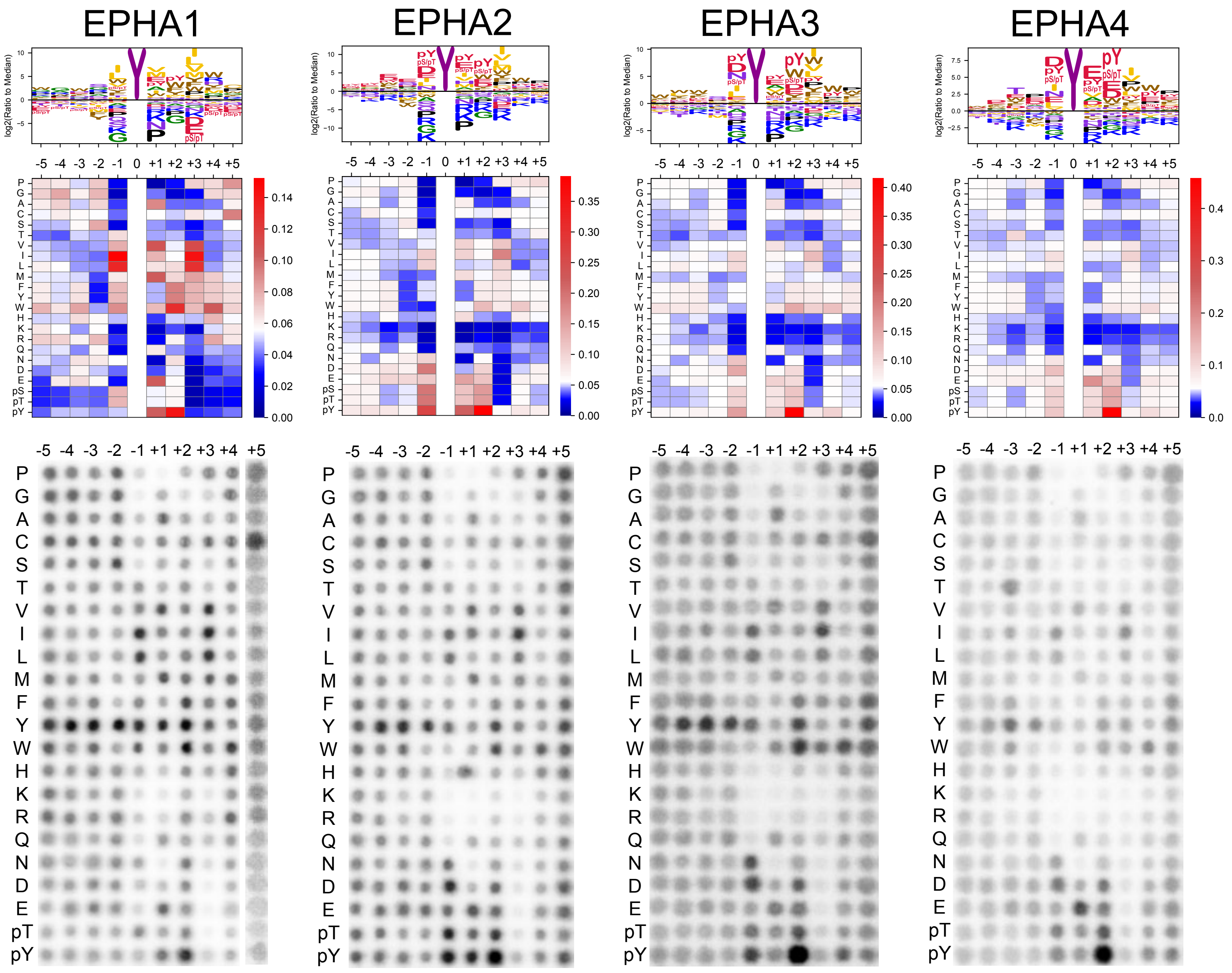
EPHA1
EPHA2
EPHA3
EPHA4
log2(Ratio to Median)
-5 -4 -3 -2 -1 0 +1 +2 +3 +4 +5
P G A C S T V I L M F Y W H K R Q N D E pS pT pY
-5 -4 -3 -2 -1 +1 +2 +3 +4 +5
P G A C S T V I L M F Y W H K R Q N D E pT pY

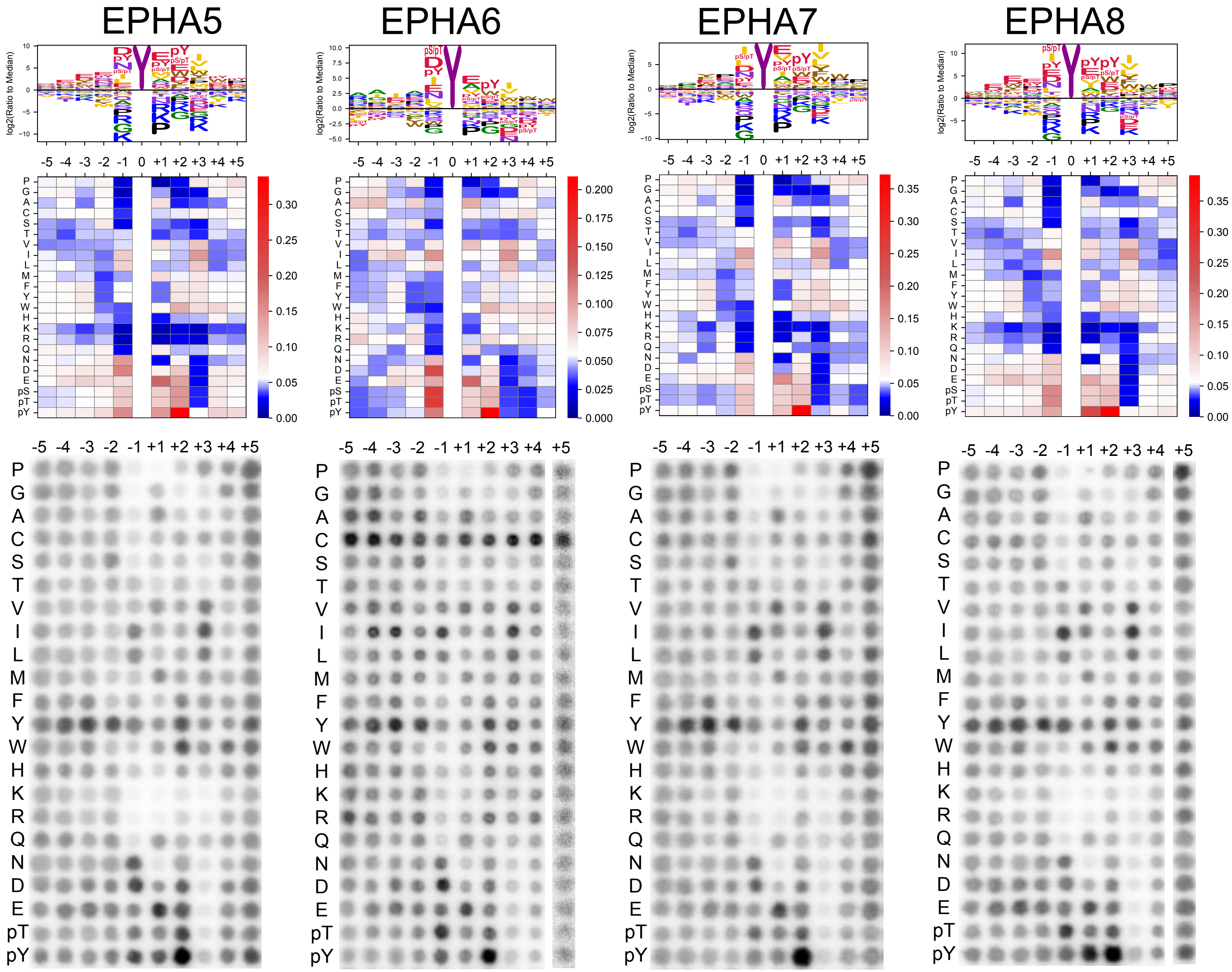

EPHA5
EPHA6
EPHA7
EPHA8
log2(Ratio to Median)
-5 -4 -3 -2 -1 0 +1 +2 +3 +4 +5
P G A C S T V I L M F Y W H K R Q N D E pS pT pY
-5 -4 -3 -2 -1 +1 +2 +3 +4 +5
P G A C S T V I L M F Y W H K R Q N D E pT pY

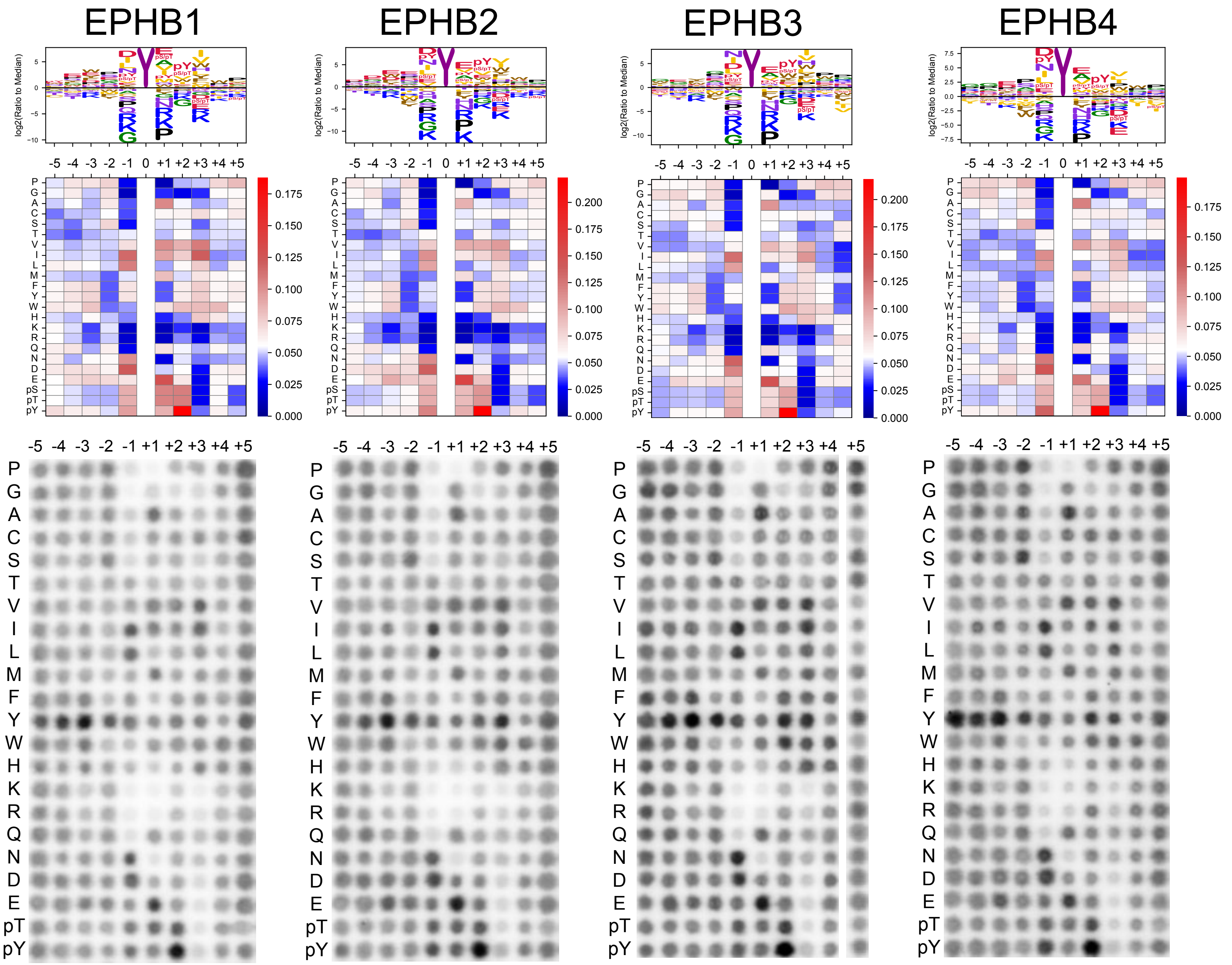

EPHB1
EPHB2
EPHB3
EPHB4
log2(Ratio to Median)
-5 -4 -3 -2 -1 0 +1 +2 +3 +4 +5
P G A C S T V I L M F Y W H K R Q N D E pS pT pY
-5 -4 -3 -2 -1 +1 +2 +3 +4 +5
P G A C S T V I L M F Y W H K R Q N D E pT pY

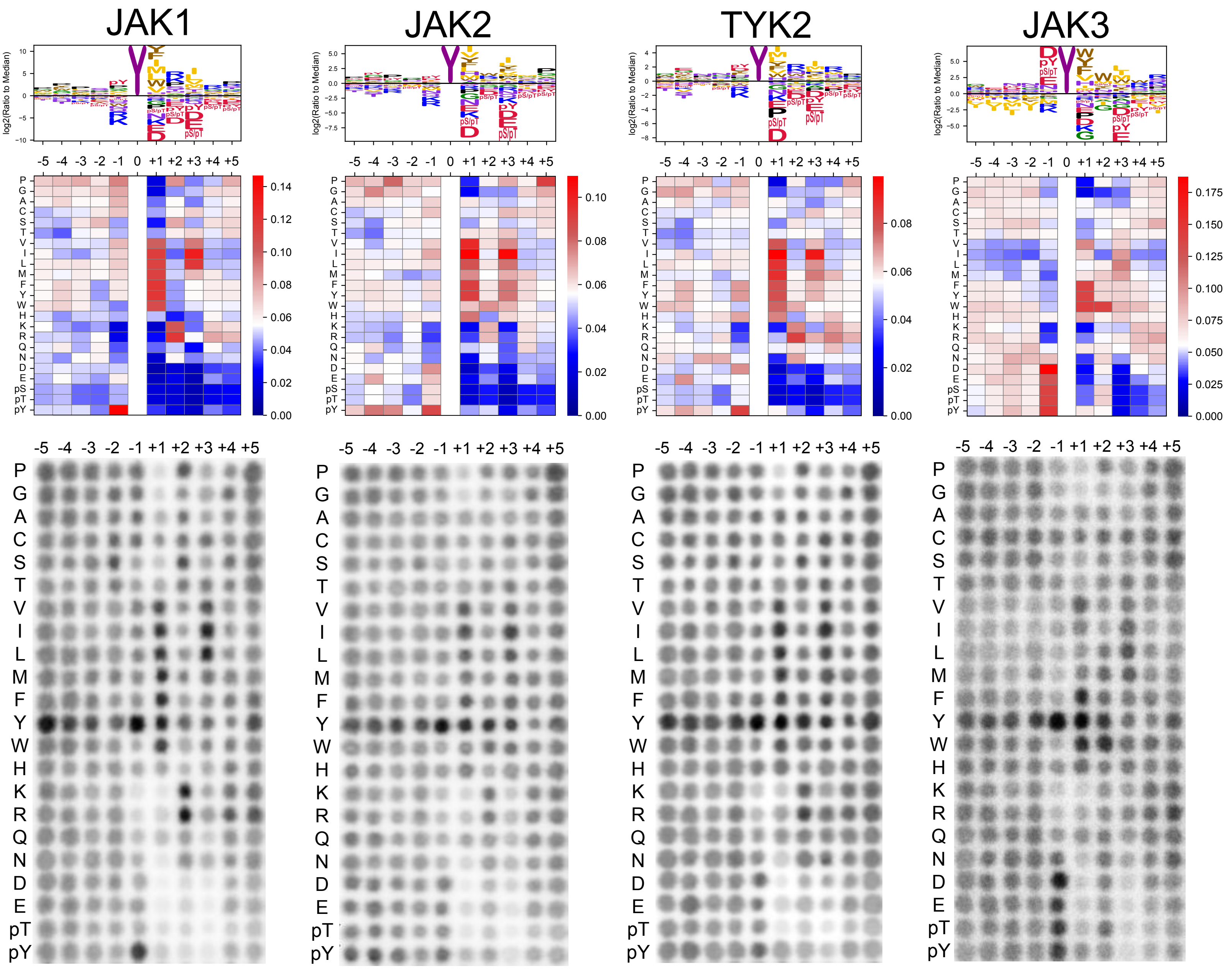
JAK1
JAK2
TYK2
JAK3
log2(Ratio to Median)
-5 -4 -3 -2 -1 0 +1 +2 +3 +4 +5
P G A C S T V I L M F Y W H K R Q N D E pS pT pY
-5 -4 -3 -2 -1 +1 +2 +3 +4 +5
P G A C S T V I L M F Y W H K R Q N D E pT pY

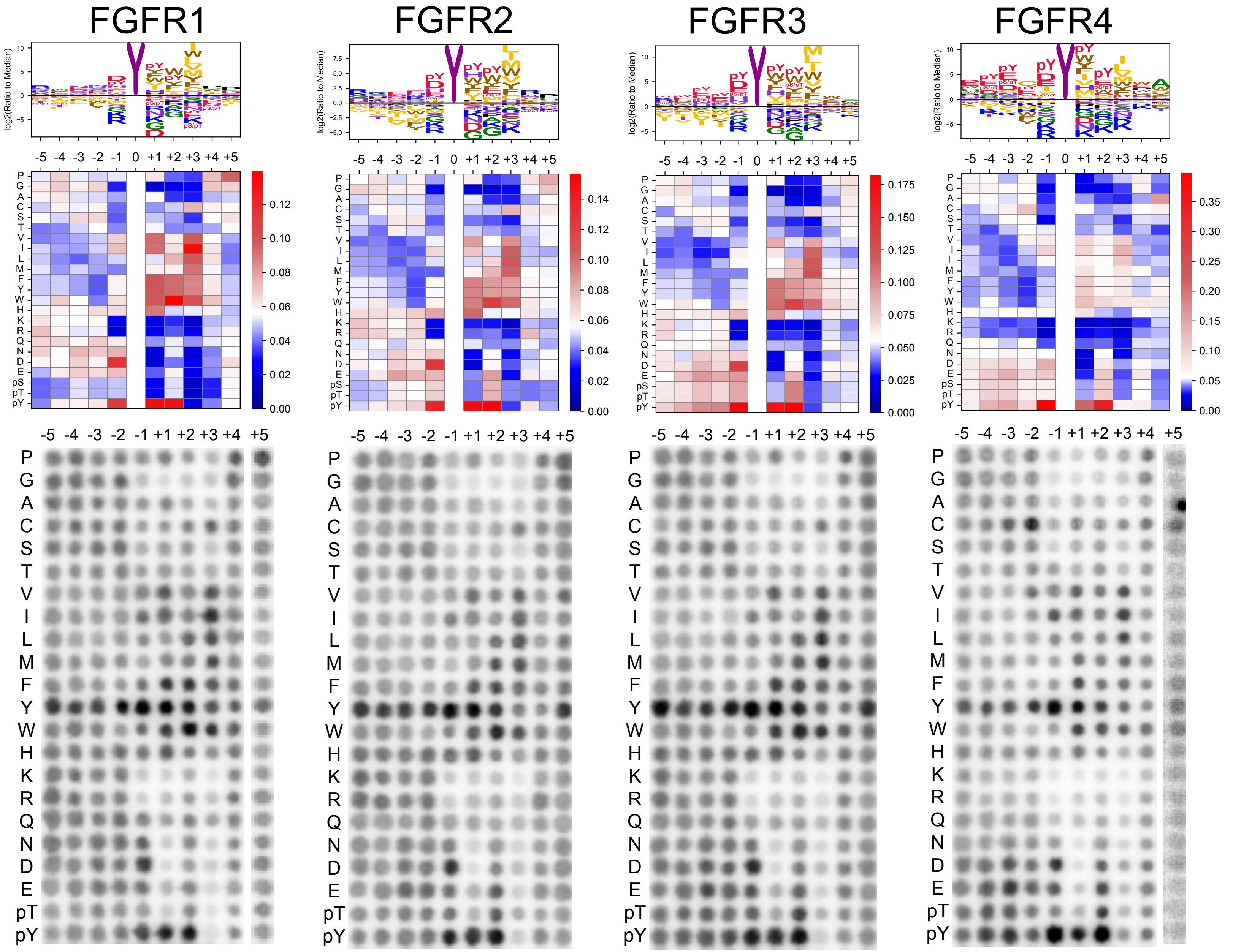

FGFR1
FGFR2
FGFR3
FGFR4
log2(Ratio to Median)
-5 -4 -3 -2 -1 0 +1 +2 +3 +4 +5
P G A C S T V I L M F Y W H K R Q N D E pS pT pY
P G A C S T V I L M F Y W H K R Q N D E pT pY

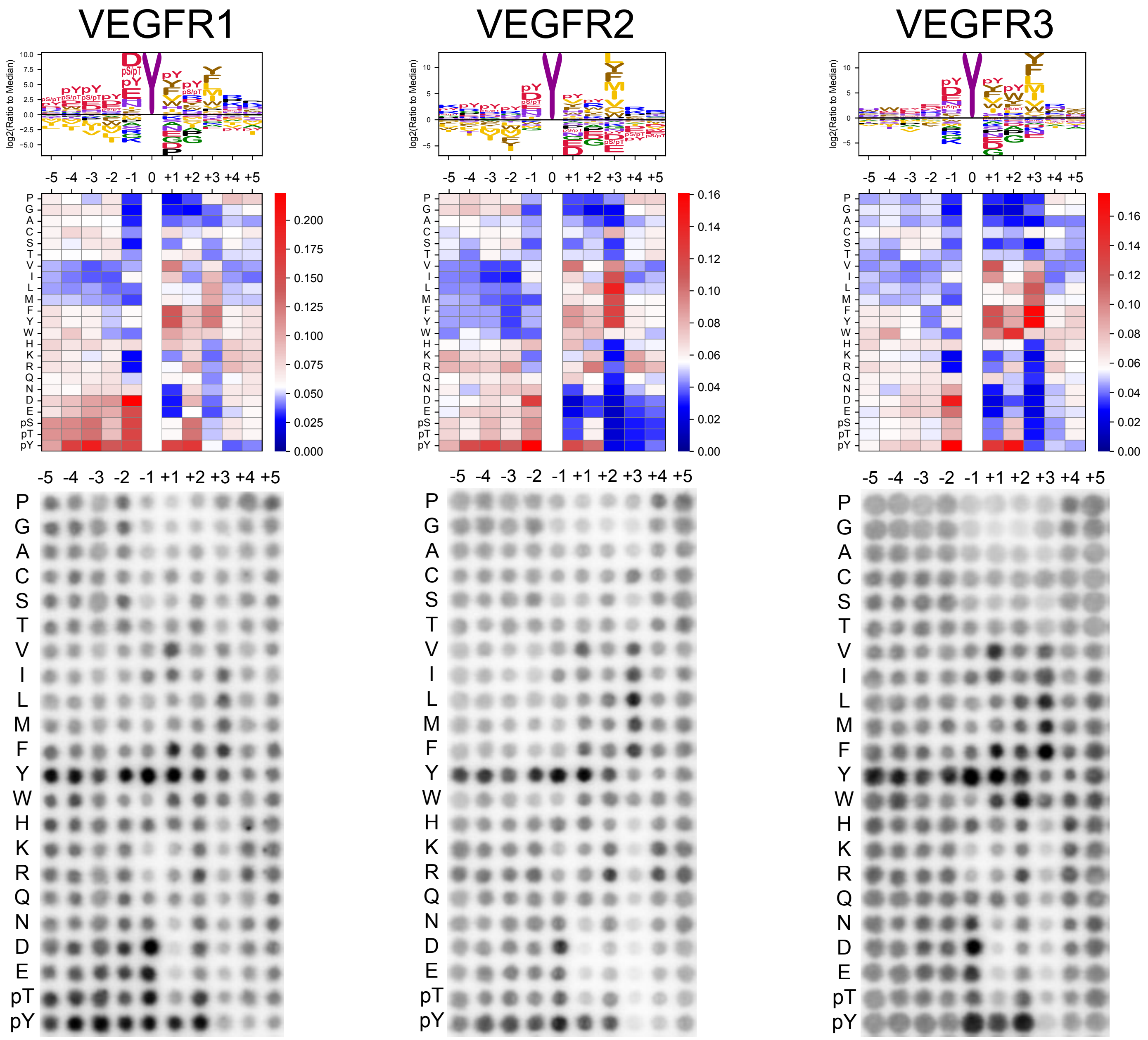

VEGFR1
VEGFR2
VEGFR3
log2(Ratio to Median)
-5 -4 -3 -2 -1 0 +1 +2 +3 +4 +5
P G A C S T V I L M F Y W H K R Q N D E pS pT pY
-5 -4 -3 -2 -1 +1 +2 +3 +4 +5
P G A C S T V I L M F Y W H K R Q N D E pT pY

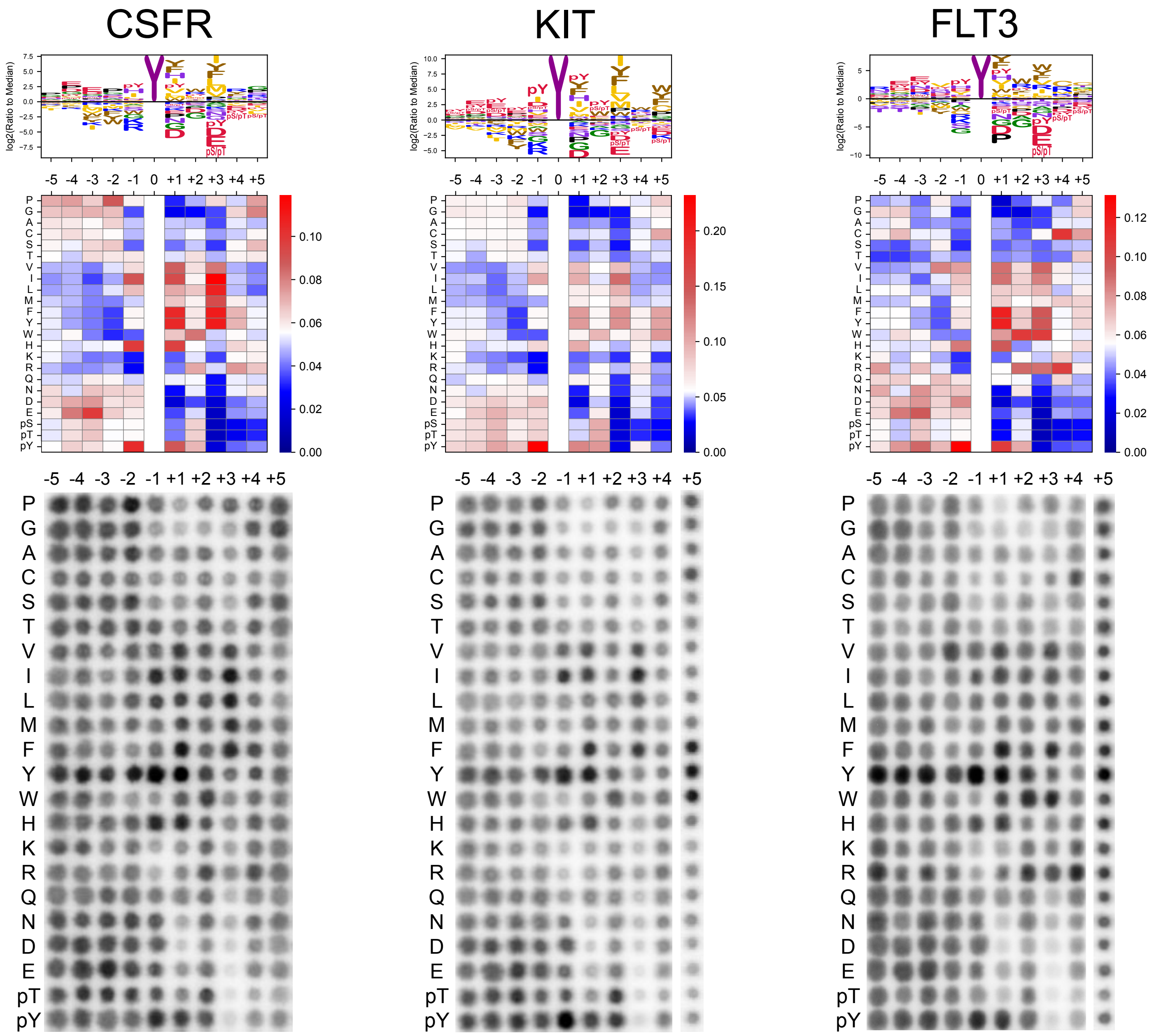

CSFR
KIT
FLT3
log2(Ratio to Median)
-5 -4 -3 -2 -1 0 +1 +2 +3 +4 +5
P G A C S T V I L M F Y W H K R Q N D E pS pT pY
P G A C S T V I L M F Y W H K R Q N D E pT pY

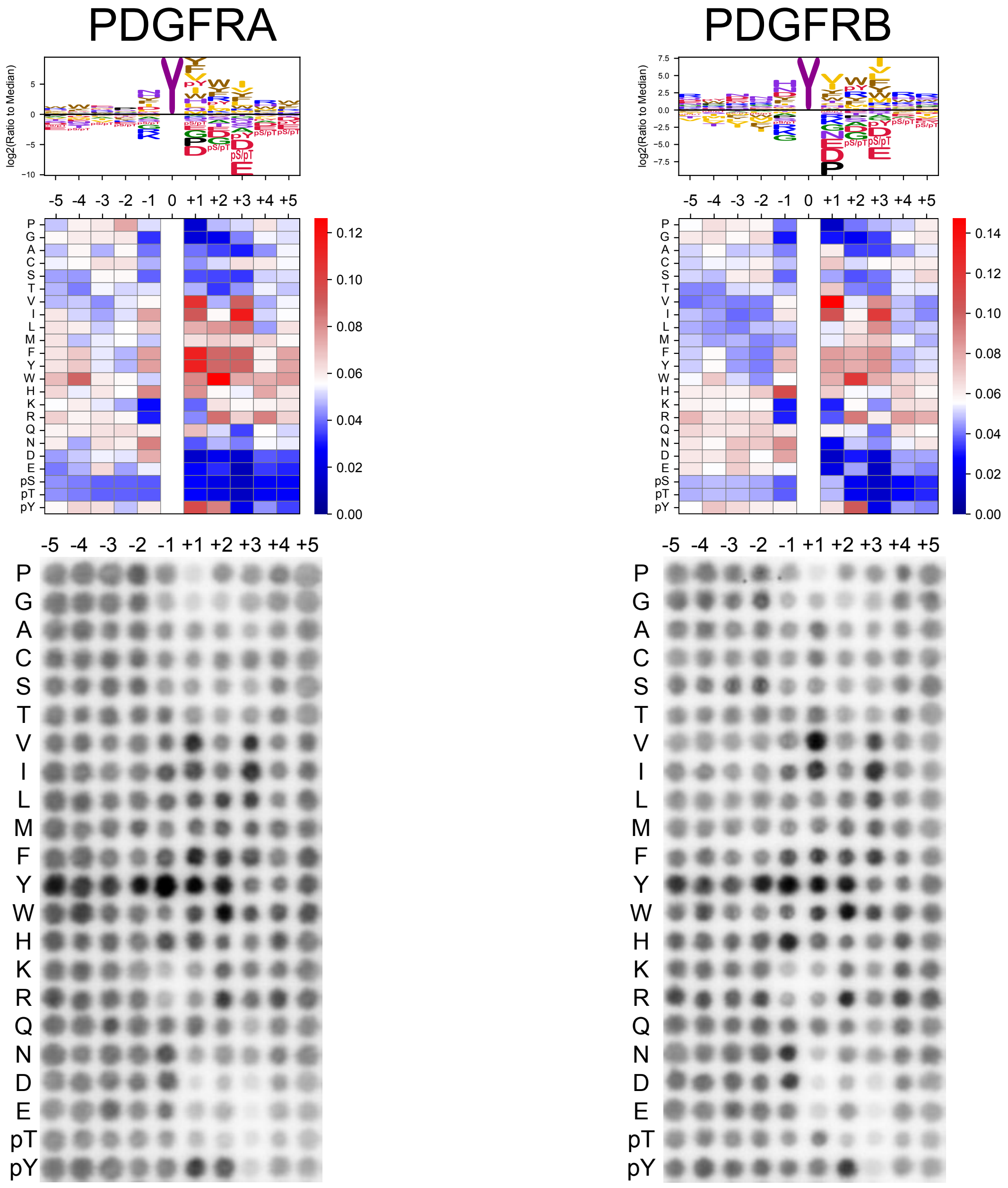
PDGFRA
PDGFRB
log2(Ratio to Median)
-5 -4 -3 -2 -1 0 +1 +2 +3 +4 +5
P G A C S T V I L M F Y W H K R Q N D E pS pT pY
P G A C S T V I L M F Y W H K R Q N D E pT pY

TIE2

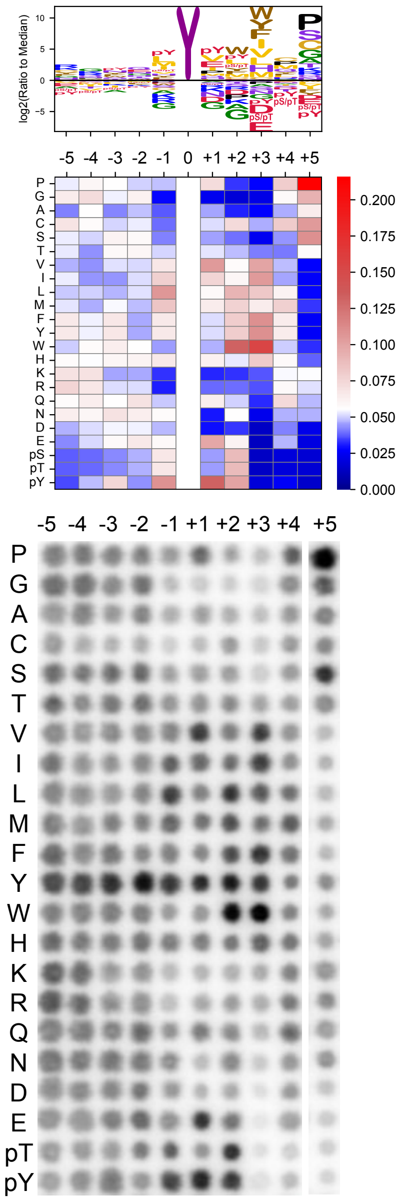
log2(Ratio to Median)
10
5
0
-5
-5 -4 -3 -2 -1 0 +1 +2 +3 +4 +5
P G A C S T V I L M F Y W H K R Q N D E pS pT pY
0.200
0.175
0.150
0.125
0.100
0.075
0.050
0.025
0.000
-5 -4 -3 -2 -1 +1 +2 +3 +4 +5
P G A C S T V I L M F Y W H K R Q N D E pT pY

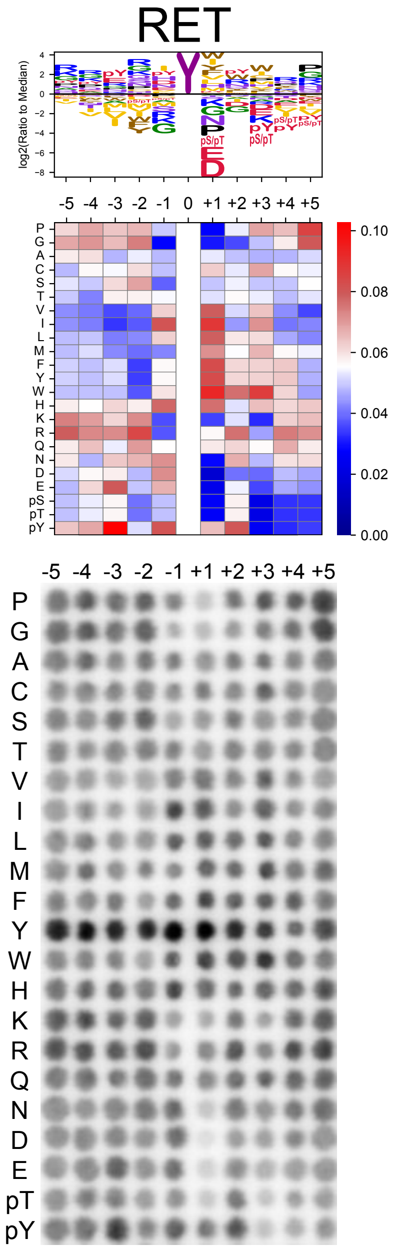
RET
log2(Ratio to Median)
-5 -4 -3 -2 -1 0 +1 +2 +3 +4 +5
P G A C S T V I L M F Y W H K R Q N D E pS pT pY
0.10 0.08 0.06 0.04 0.02 0.00
-5 -4 -3 -2 -1 +1 +2 +3 +4 +5
P G A C S T V I L M F Y W H K R Q N D E pT pY

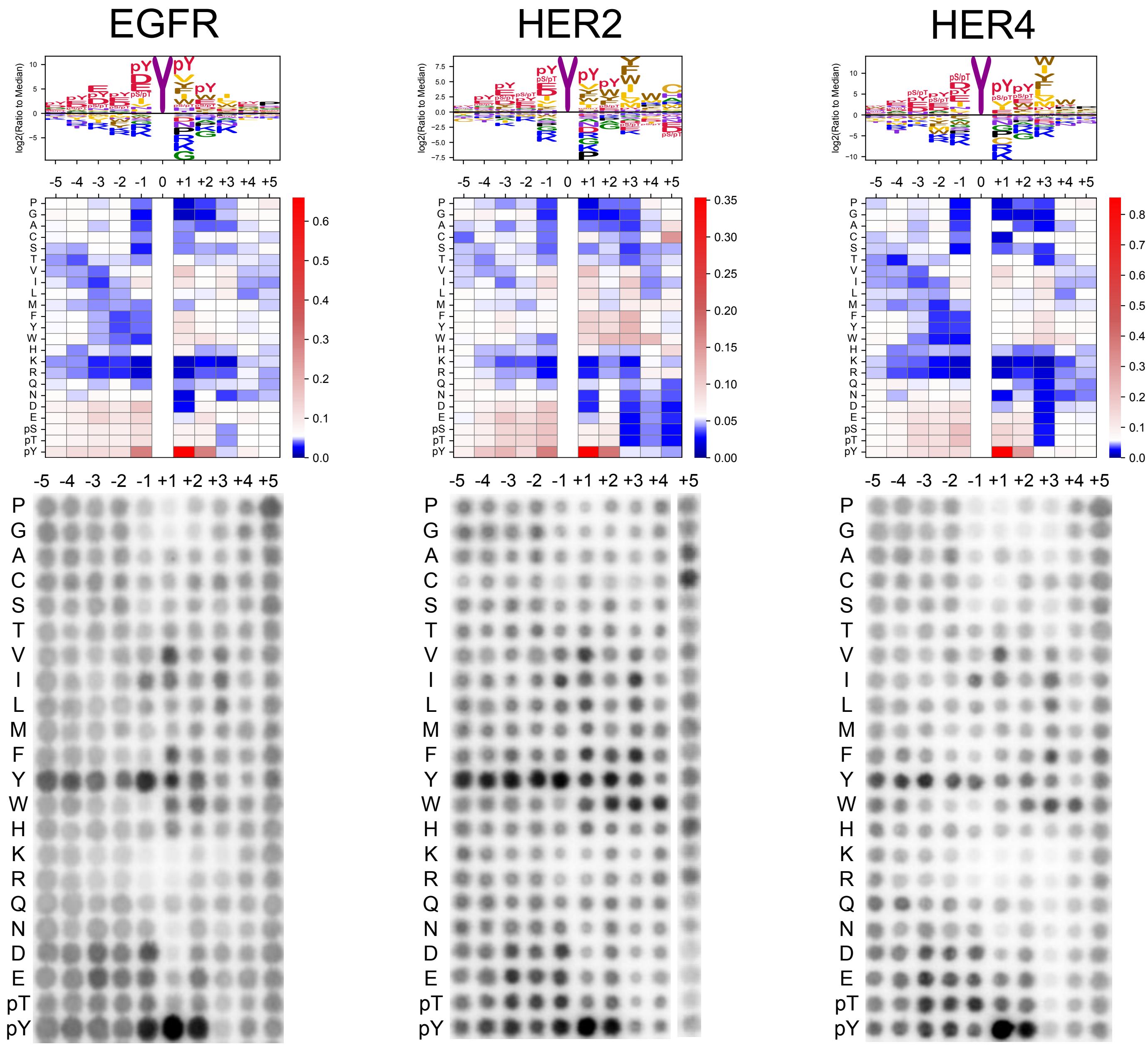
EGFR
HER2
HER4
log2(Ratio to Median)
-5 -4 -3 -2 -1 0 +1 +2 +3 +4 +5
P G A C S T V I L M F Y W H K R Q N D E pS pT pY
P G A C S T V I L M F Y W H K R Q N D E pT pY

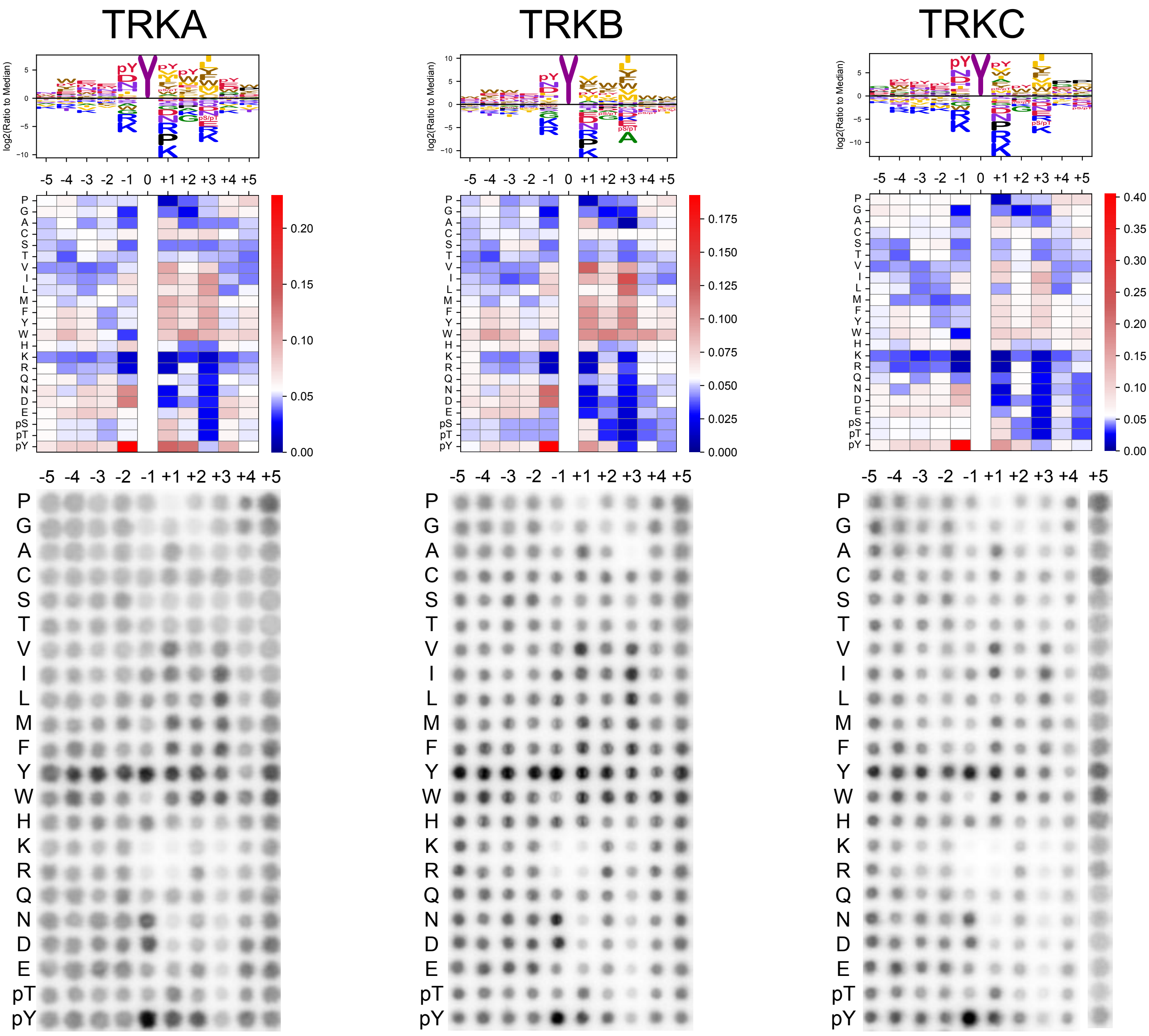
TRKA
TRKB
TRKC
log2(Ratio to Median)
-5 -4 -3 -2 -1 0 +1 +2 +3 +4 +5
P G A C S T V I L M F Y W H K R Q N D E pS pT pY
P G A C S T V I L M F Y W H K R Q N D E pT pY

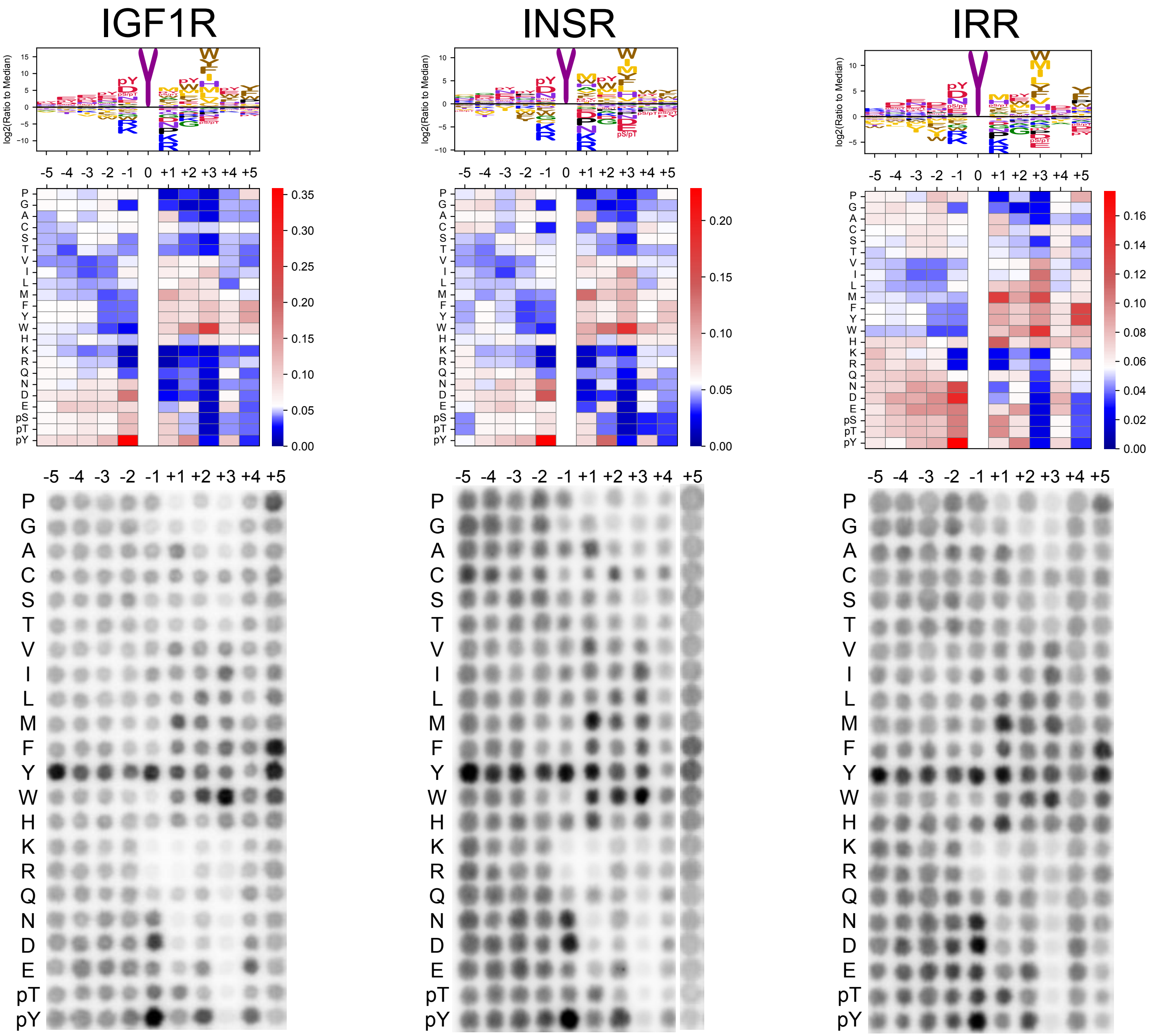
IGF1R
INSR
IRR
log2(Ratio to Median)
-5 -4 -3 -2 -1 0 +1 +2 +3 +4 +5
P G A C S T V I L M F Y W H K R Q N D E pS pT pY
P G A C S T V I L M F Y W H K R Q N D E pT pY

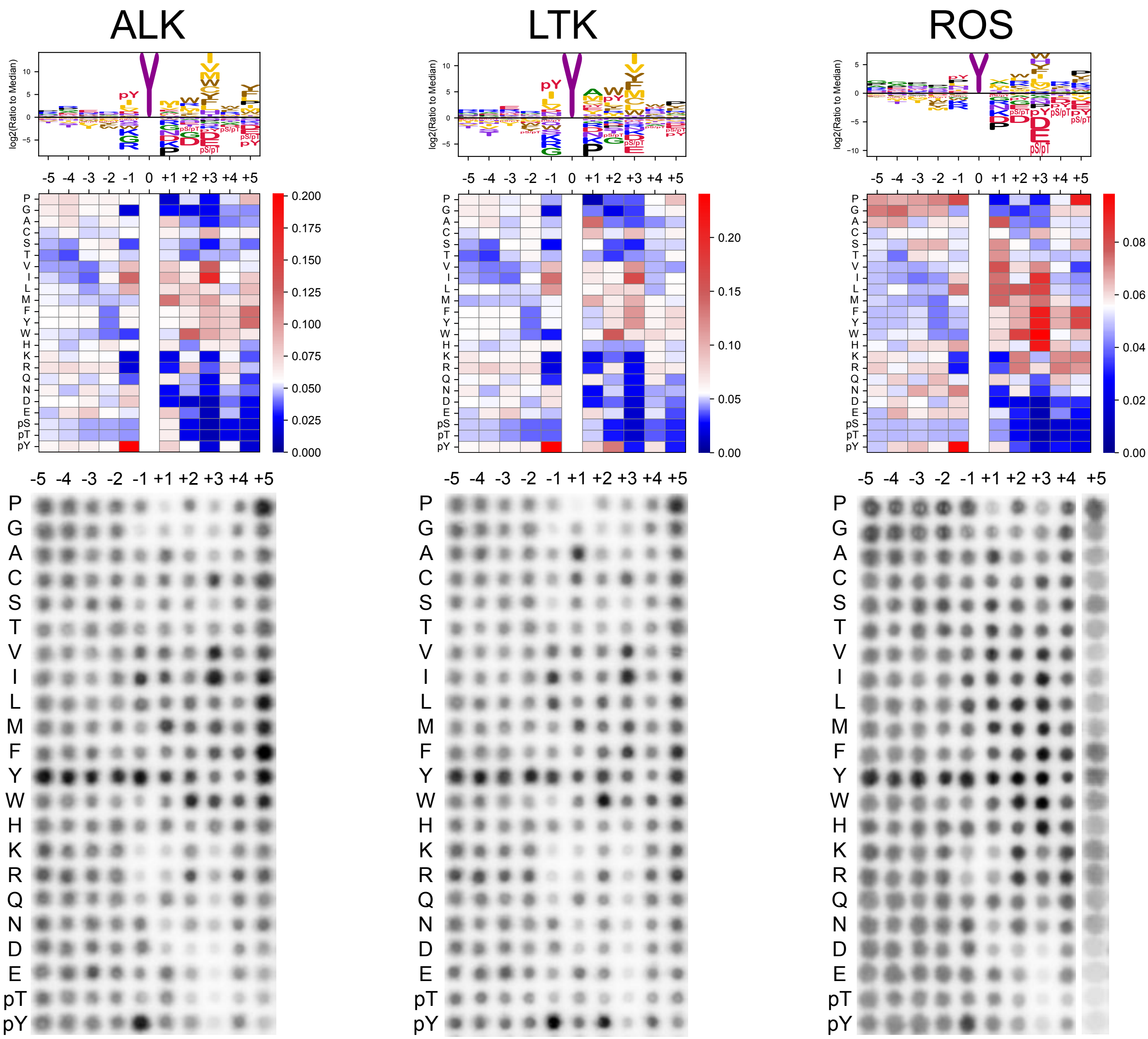

ALK
LTK
ROS
log2(Ratio to Median)
-5 -4 -3 -2 -1 0 +1 +2 +3 +4 +5
P G A C S T V I L M F Y W H K R Q N D E pS pT pY
0.200 0.175 0.150 0.125 0.100 0.075 0.050 0.025 0.000
0.20 0.15 0.10 0.05 0.00
0.08 0.06 0.04 0.02 0.00
-5 -4 -3 -2 -1 +1 +2 +3 +4 +5
P G A C S T V I L M F Y W H K R Q N D E pT pY

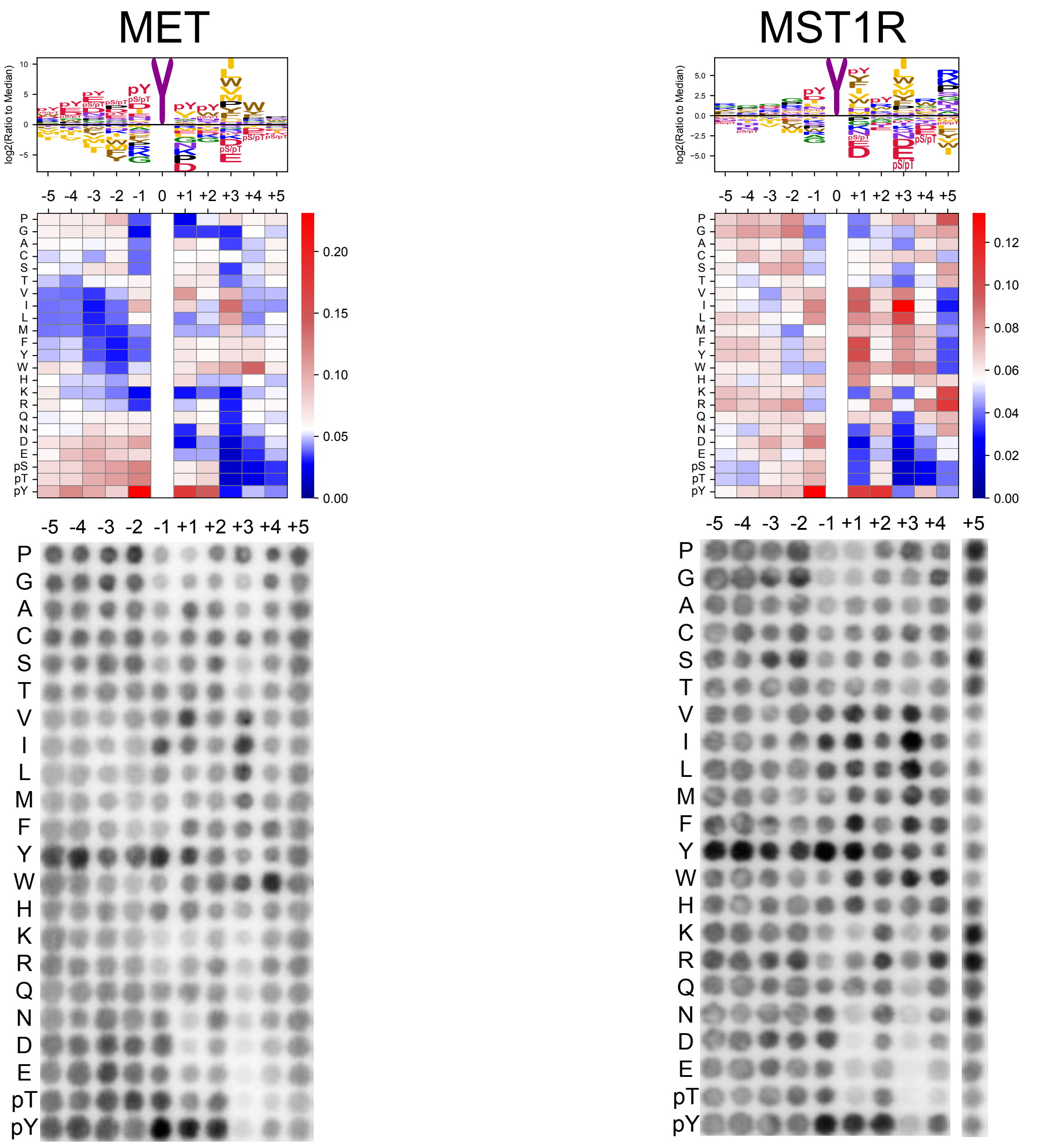

MET
MST1R
log2(Ratio to Median)
-5 -4 -3 -2 -1 0 +1 +2 +3 +4 +5
P G A C S T V I L M F Y W H K R Q N D E pS pT pY
0.00 0.05 0.10 0.15 0.20
0.00 0.02 0.04 0.06 0.08 0.10 0.12
-5 -4 -3 -2 -1 +1 +2 +3 +4 +5
P G A C S T V I L M F Y W H K R Q N D E pT pY

MUSK

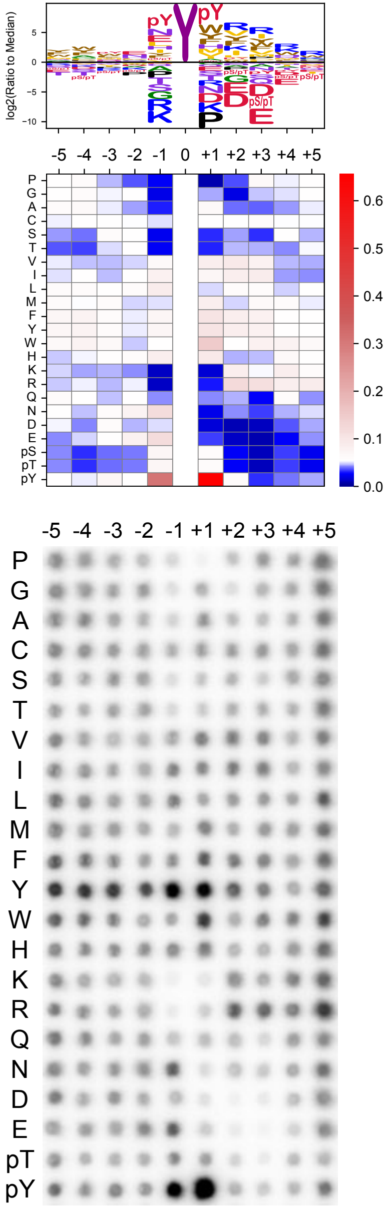

log2(Ratio to Median)
-5 -4 -3 -2 -1 0 +1 +2 +3 +4 +5
P G A C S T V I L M F Y W H K R Q N D E pS pT pY
0.6 0.5 0.4 0.3 0.2 0.1 0.0
-5 -4 -3 -2 -1 +1 +2 +3 +4 +5
P G A C S T V I L M F Y W H K R Q N D E pT pY

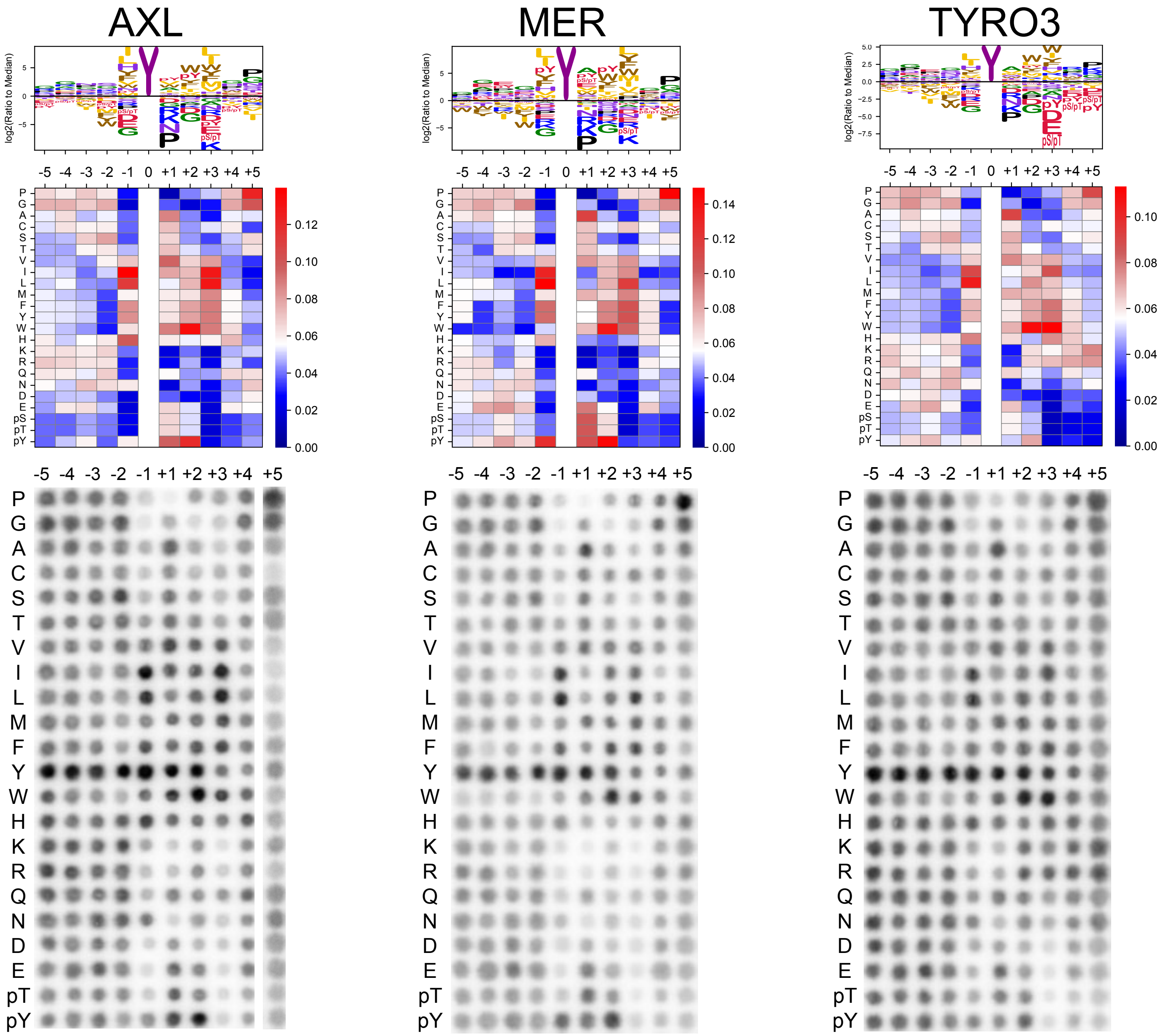

AXL
MER
TYRO3
log2(Ratio to Median)
-5 -4 -3 -2 -1 0 +1 +2 +3 +4 +5
P G A C S T V I L M F Y W H K R Q N D E pS pT pY
P G A C S T V I L M F Y W H K R Q N D E pT pY

DDR1

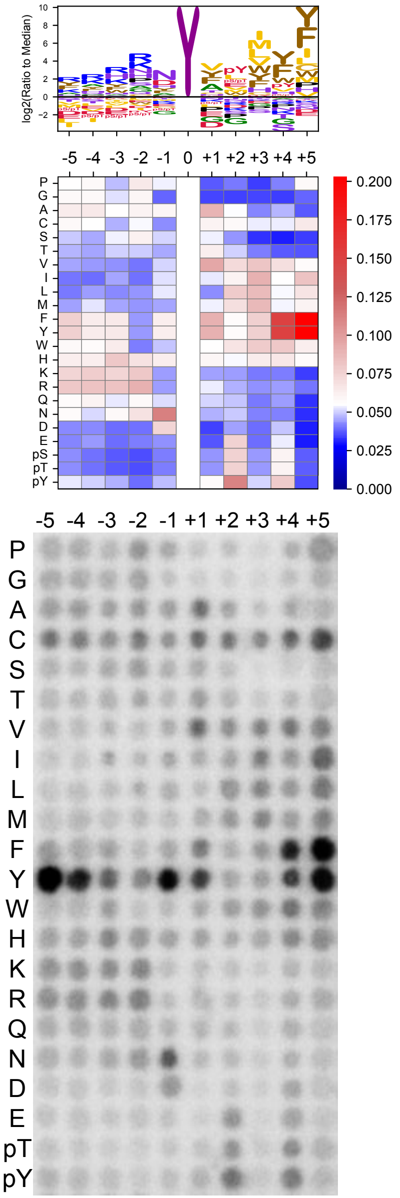
log2(Ratio to Median)
10 8 6 4 2 0 -2
-5 -4 -3 -2 -1 0 +1 +2 +3 +4 +5
P G A C S T V I L M F Y W H K R Q N D E pS pT pY
0.200 0.175 0.150 0.125 0.100 0.075 0.050 0.025 0.000
-5 -4 -3 -2 -1 +1 +2 +3 +4 +5
P G A C S T V I L M F Y W H K R Q N D E pT pY

DDR2

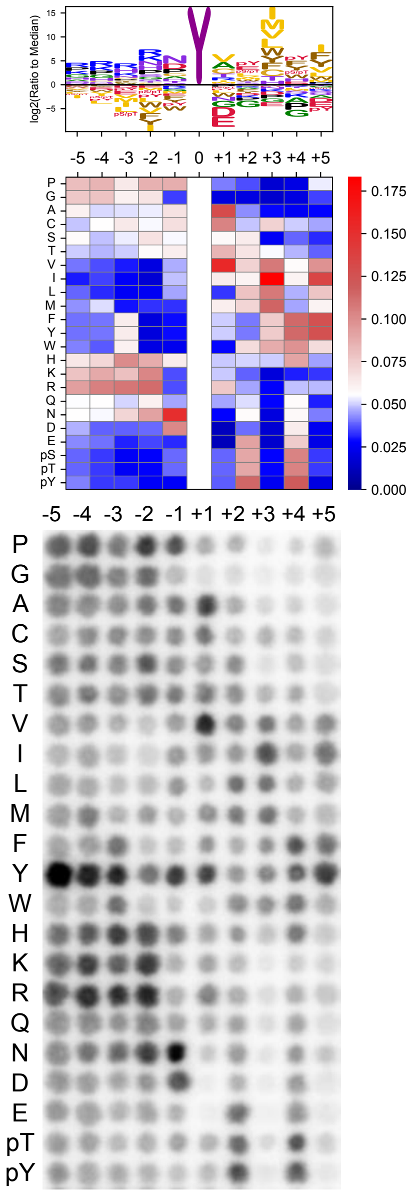
log2(Ratio to Median)
15 10 5 0 -5
-5 -4 -3 -2 -1 0 +1 +2 +3 +4 +5
P G A C S T V I L M F Y W H K R Q N D E pS pT pY
0.175 0.150 0.125 0.100 0.075 0.050 0.025 0.000
-5 -4 -3 -2 -1 +1 +2 +3 +4 +5
P G A C S T V I L M F Y W H K R Q N D E pT pY

TNNI3K

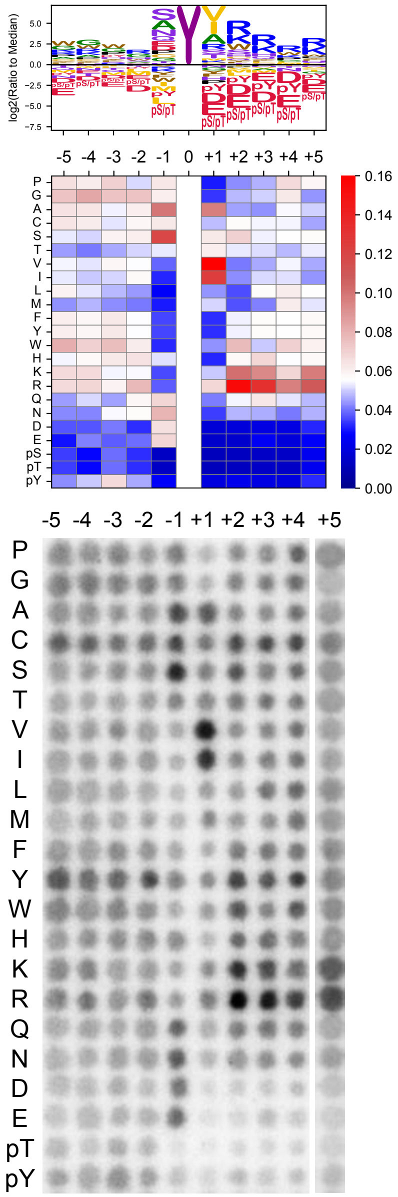
log2(Ratio to Median)
5.0
2.5
0.0
−2.5
−5.0
−7.5
-5 -4 -3 -2 -1 0 +1 +2 +3 +4 +5
P
G
A
C
S
T
V
I
L
M
F
Y
W
H
K
R
Q
N
D
E
pS
pT
pY
0.16
0.14
0.12
0.10
0.08
0.06
0.04
0.02
0.00
-5 -4 -3 -2 -1 +1 +2 +3 +4 +5
P
G
A
C
S
T
V
I
L
M
F
Y
W
H
K
R
Q
N
D
E
pT
pY

NEK10

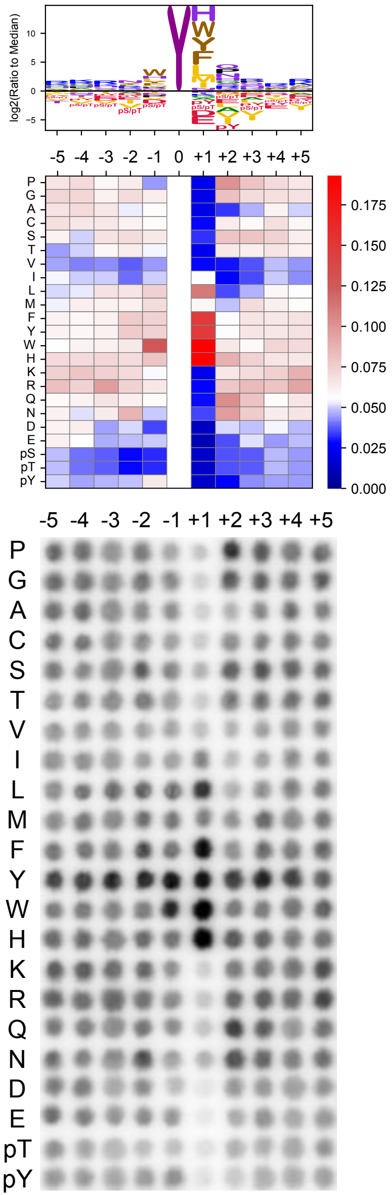
log2(Ratio to Median)
-5 -4 -3 -2 -1 0 +1 +2 +3 +4 +5
P G A C S T V I L M F Y W H K R Q N D E pS pT pY
0.175 0.150 0.125 0.100 0.075 0.050 0.025 0.000
P G A C S T V I L M F Y W H K R Q N D E pT pY

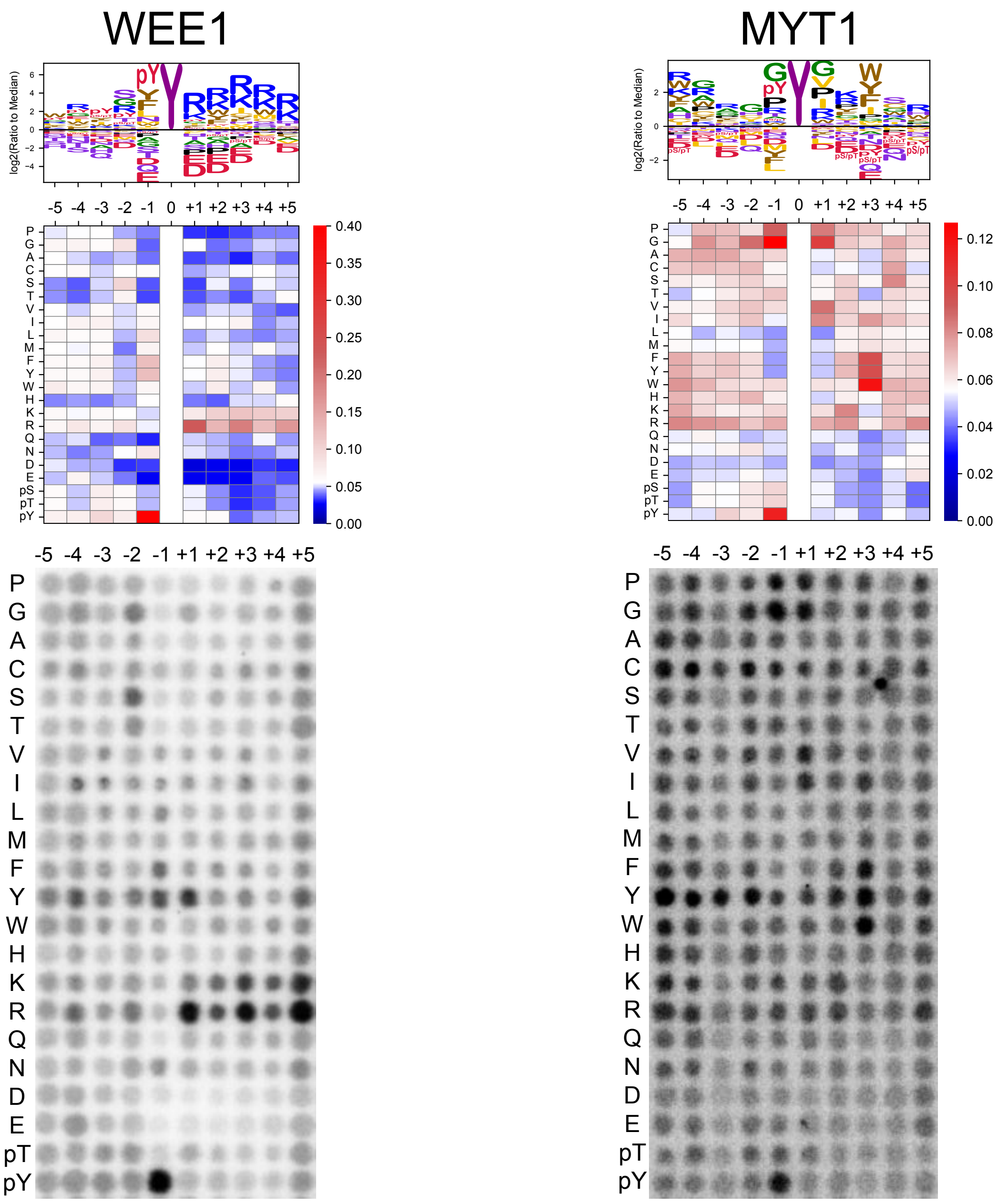

WEE1
MYT1
log2(Ratio to Median)

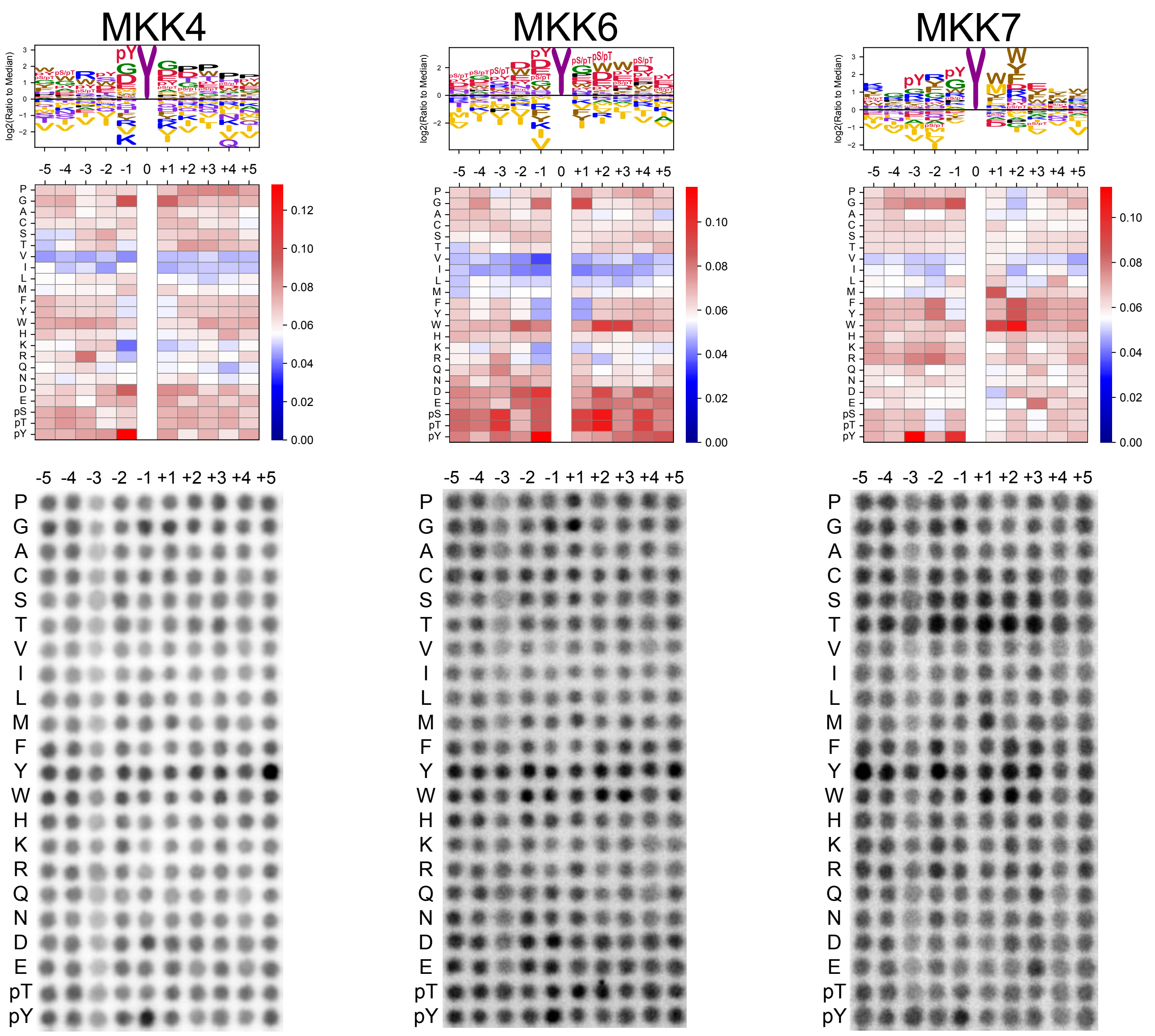
MKK4
MKK6
MKK7
log2(Ratio to Median)
-5 -4 -3 -2 -1 0 +1 +2 +3 +4 +5
P G A C S T V I L M F Y W H K R Q N D E pS pT pY
0.12 0.10 0.08 0.06 0.04 0.02 0.00
-5 -4 -3 -2 -1 +1 +2 +3 +4 +5
P G A C S T V I L M F Y W H K R Q N D E pT pY

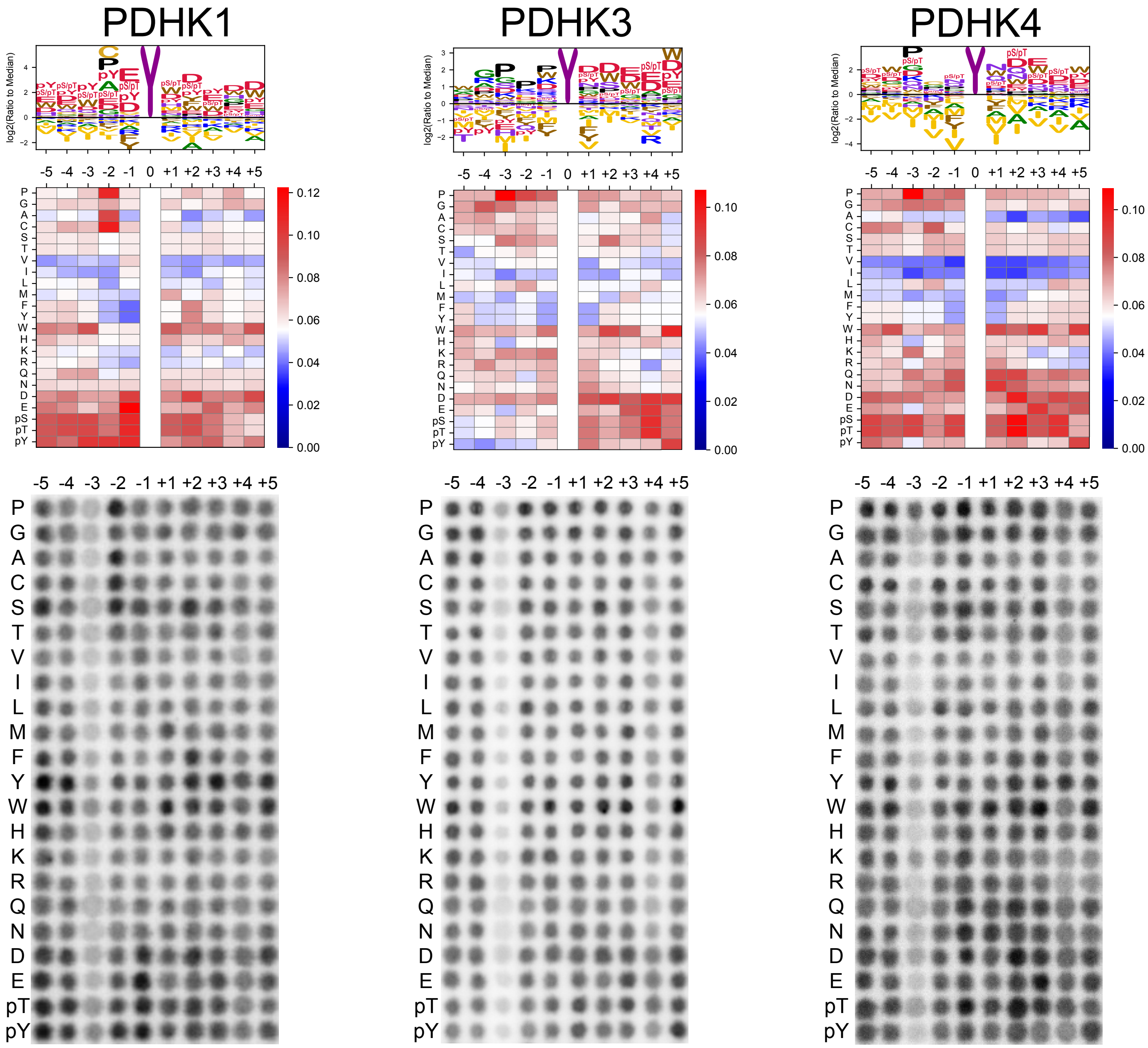

PDHK1
PDHK3
PDHK4
log2(Ratio to Median)
-5 -4 -3 -2 -1 0 +1 +2 +3 +4 +5
P G A C S T V I L M F Y W H K R Q N D E pS pT pY
-5 -4 -3 -2 -1 +1 +2 +3 +4 +5
P G A C S T V I L M F Y W H K R Q N D E pT pY

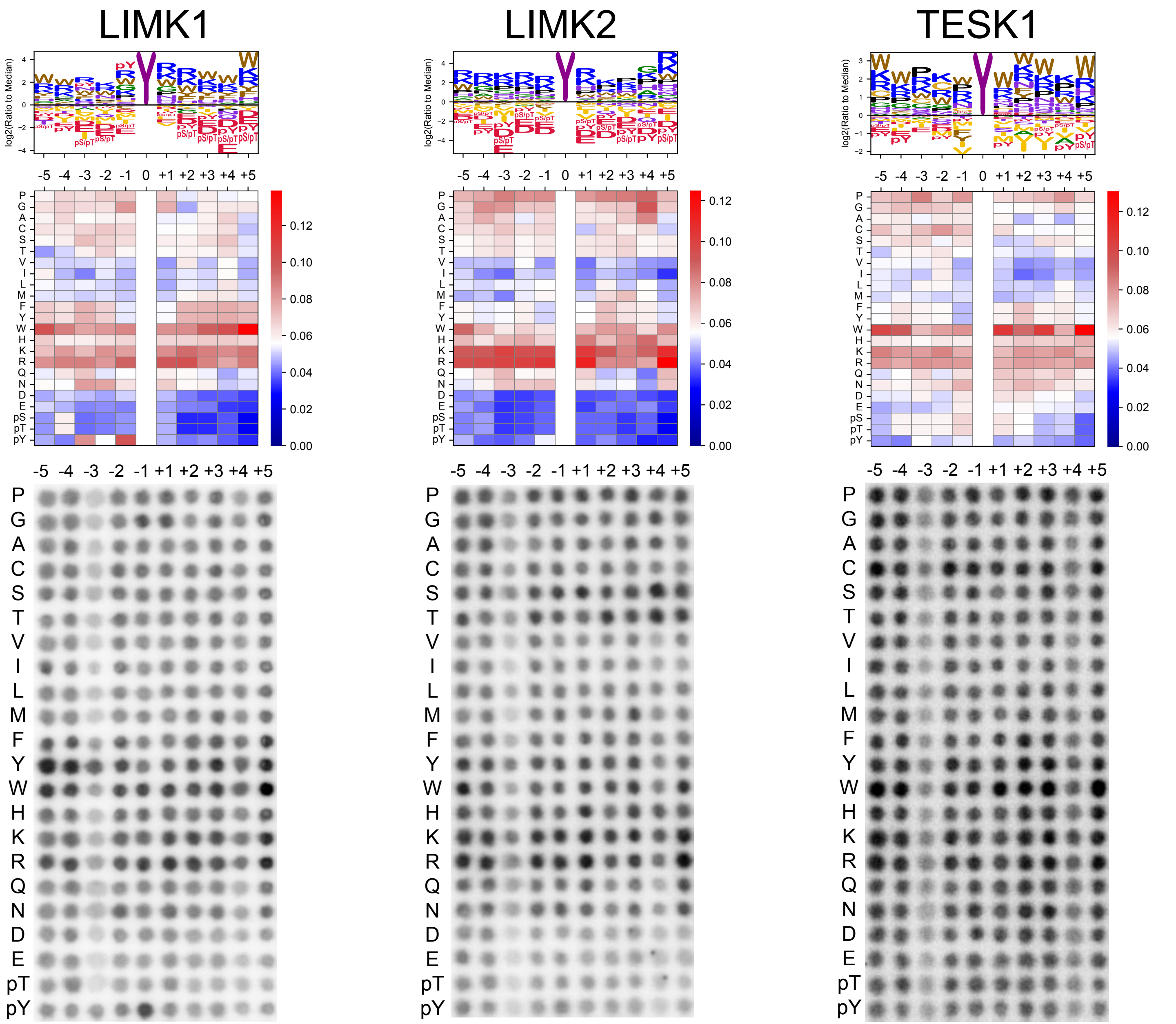

LIMK1
LIMK2
TESK1
log2(Ratio to Median)
-5 -4 -3 -2 -1 0 +1 +2 +3 +4 +5
P G A C S T V I L M F Y W H K R Q N D E pS pT pY
-5 -4 -3 -2 -1 +1 +2 +3 +4 +5
P G A C S T V I L M F Y W H K R Q N D E pT pY

BMPR2

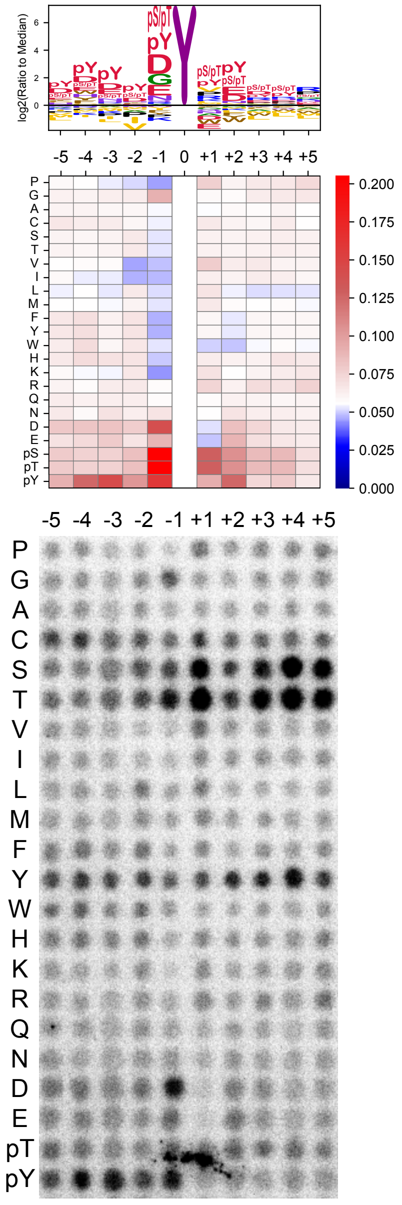

log2(Ratio to Median)
-5 -4 -3 -2 -1 0 +1 +2 +3 +4 +5
P G A C S T V I L M F Y W H K R Q N D E pS pT pY
0.200 0.175 0.150 0.125 0.100 0.075 0.050 0.025 0.000
-5 -4 -3 -2 -1 +1 +2 +3 +4 +5
P G A C S T V I L M F Y W H K R Q N D E pT pY

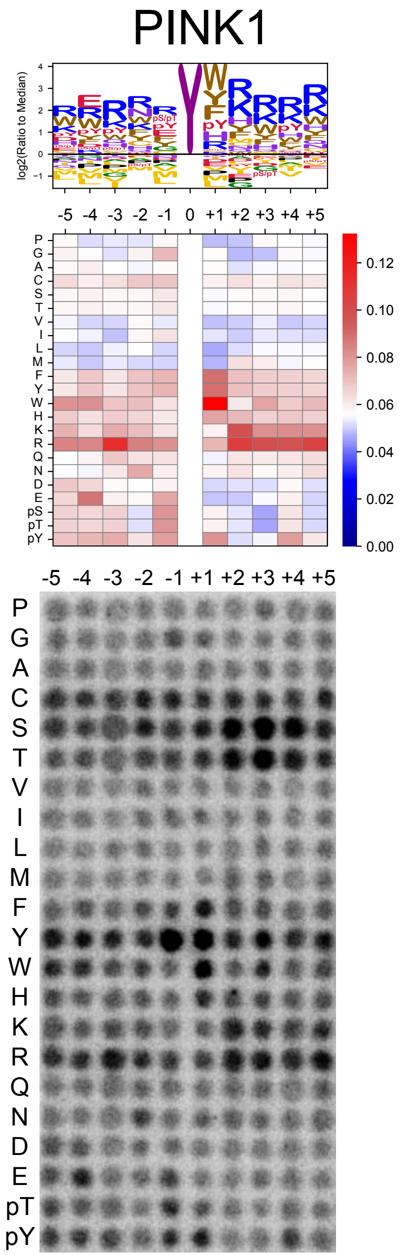

PINK1
log2(Ratio to Median)
-5 -4 -3 -2 -1 0 +1 +2 +3 +4 +5
P G A C S T V I L M F Y W H K R Q N D E pS pT pY
0.12 0.10 0.08 0.06 0.04 0.02 0.00
-5 -4 -3 -2 -1 +1 +2 +3 +4 +5
P G A C S T V I L M F Y W H K R Q N D E pT pY

C. elegans kinases

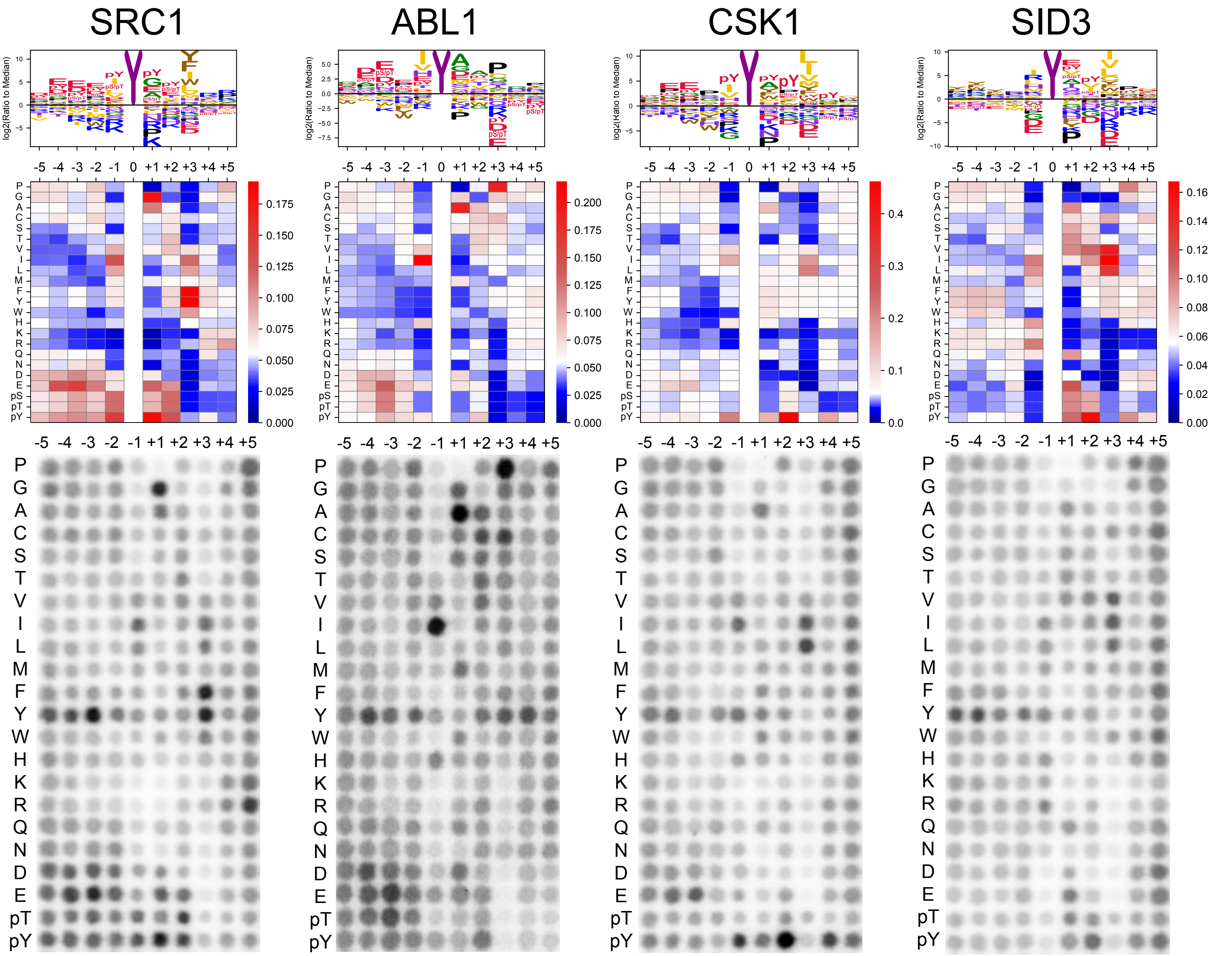
SRC1
ABL1
CSK1
SID3
log2(Ratio to Median)
-5 -4 -3 -2 -1 0 +1 +2 +3 +4 +5
P G A C S T V I L M F Y W H K R Q N D E pS pT pY
-5 -4 -3 -2 -1 +1 +2 +3 +4 +5
P G A C S T V I L M F Y W H K R Q N D E pT pY

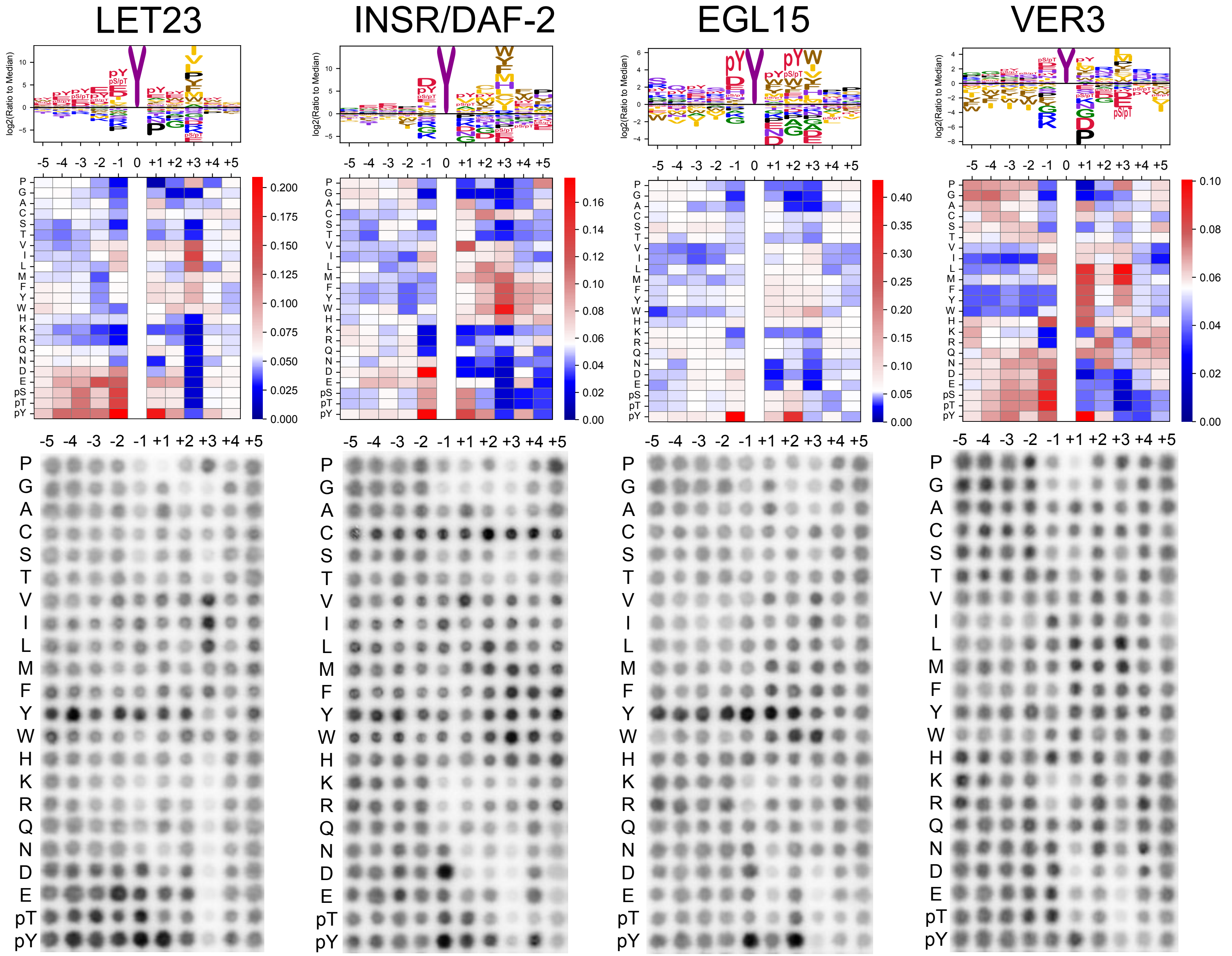
LET23
INSR/DAF-2
EGL15
VER3
log2(Ratio to Median)
-5 -4 -3 -2 -1 0 +1 +2 +3 +4 +5
P G A C S T V I L M F Y W H K R Q N D E pS pT pY
pY
pS/pT
P G A C S T V I L M F Y W H K R Q N D E pT pY

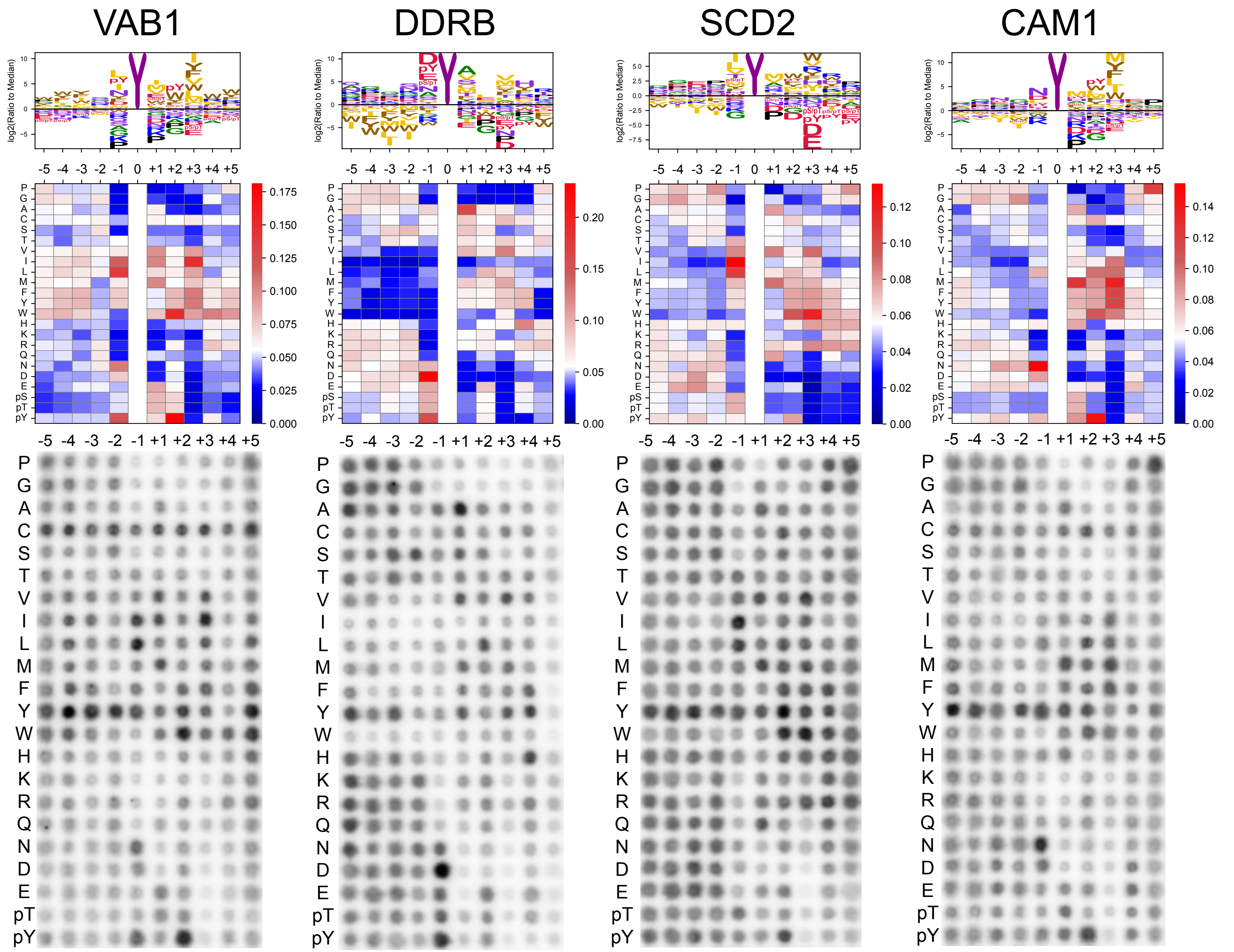

VAB1
DDRB
SCD2
CAM1
log2(Ratio to Median)
-5 -4 -3 -2 -1 0 +1 +2 +3 +4 +5
P G A C S T V I L M F Y W H K R Q N D E pS pT pY
P G A C S T V I L M F Y W H K R Q N D E pT pY

Supplementary Figure 2. Experimental data corresponding to the kinetic analyses in Extended Data Figure 9. **a-d**, Time course for JAK1 phosphorylation of indicated peptides at concentrations of 25 µM (**a**), 50 µM (**b**), 250 µM (**c**) and 500 µM (**d**). **e-h**, Time course for ZAP70 phosphorylation of indicated peptides at concentrations of 25 µM (**e**), 50 µM (**f**), 250 µM (**g**) and 500 µM (**h**).

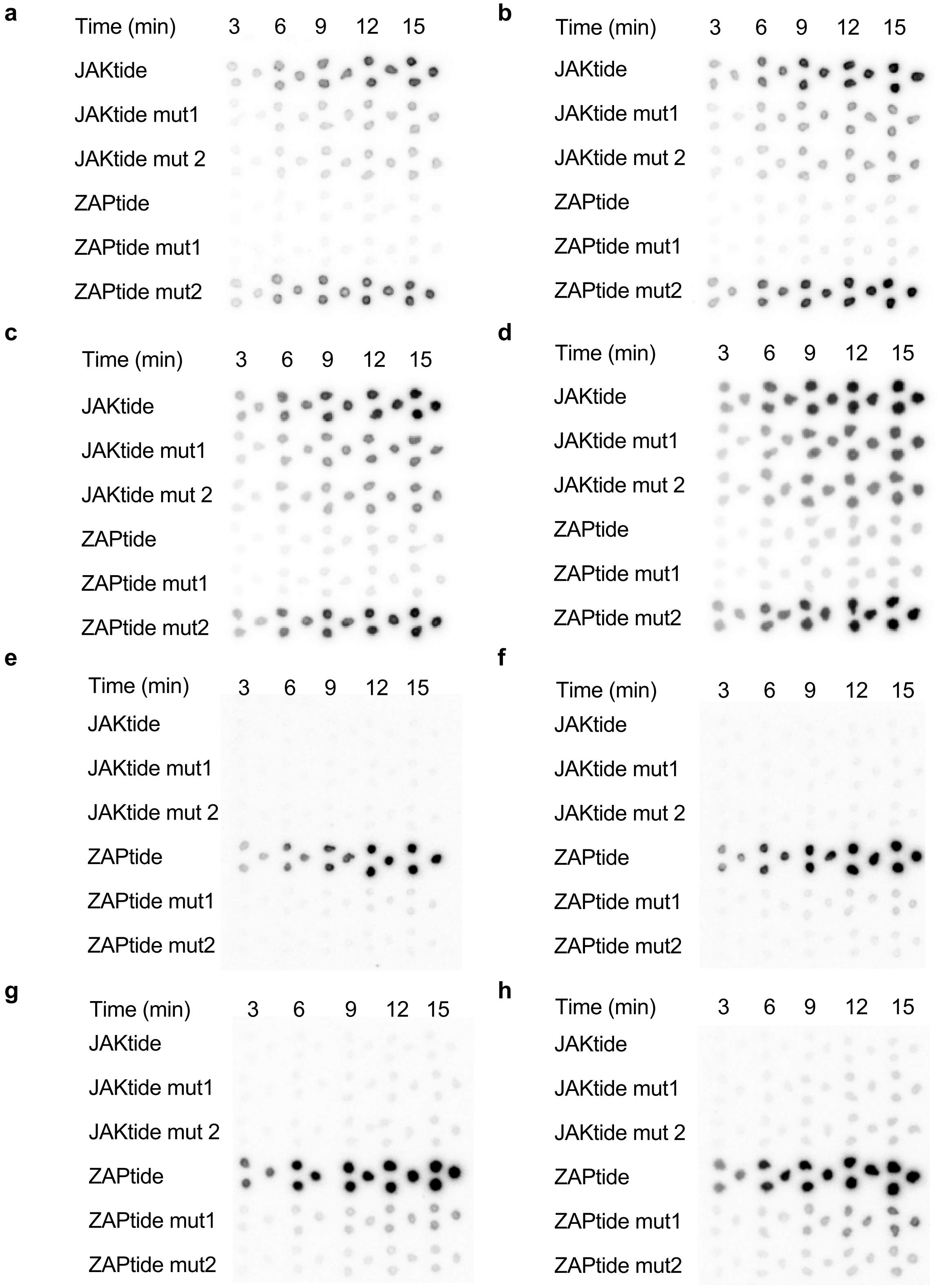

Supplementary Figure 3: Fully annotated volcano plots presented in Fig. 3 and Ext. Data Fig 10.

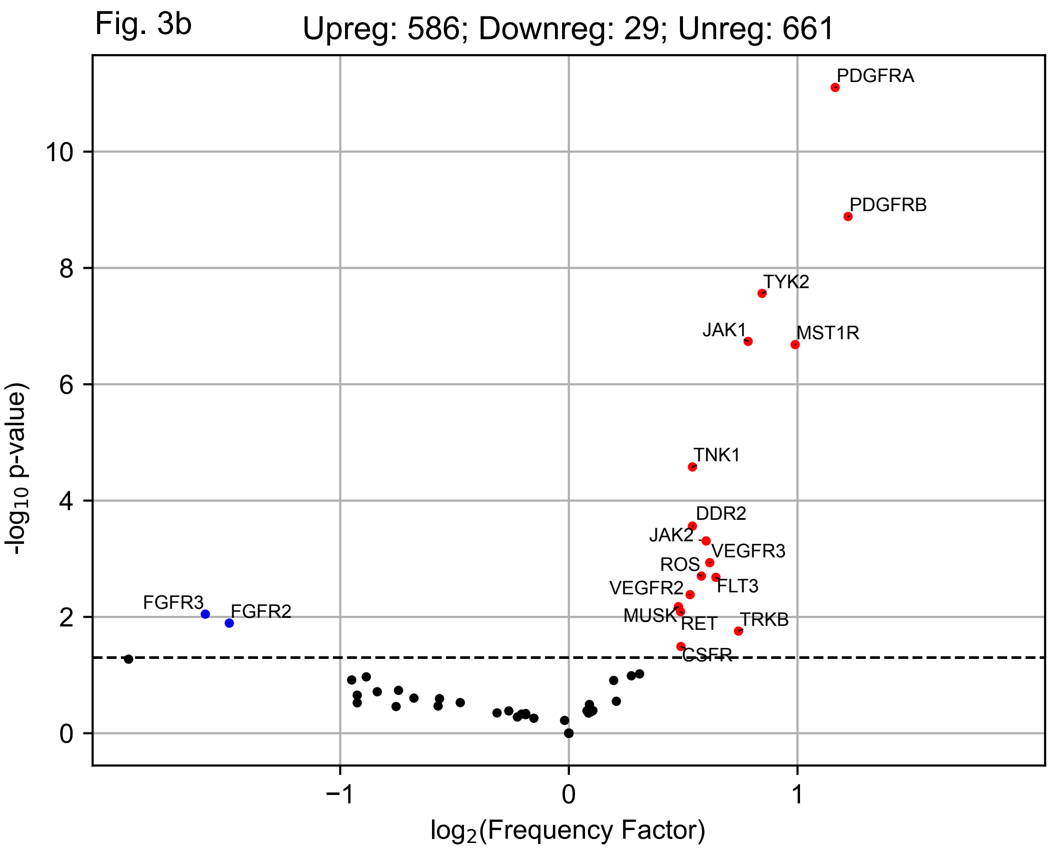

Extended Data Fig. 10a   Upreg: 346; Downreg: 44; Unreg: 359

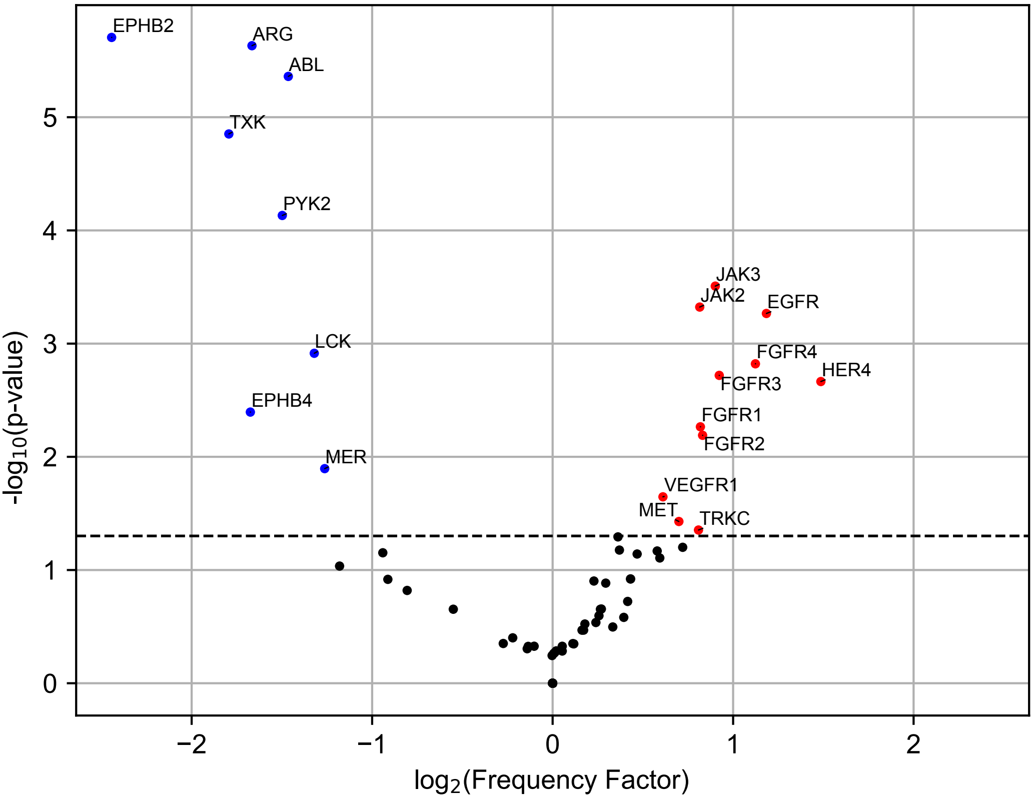

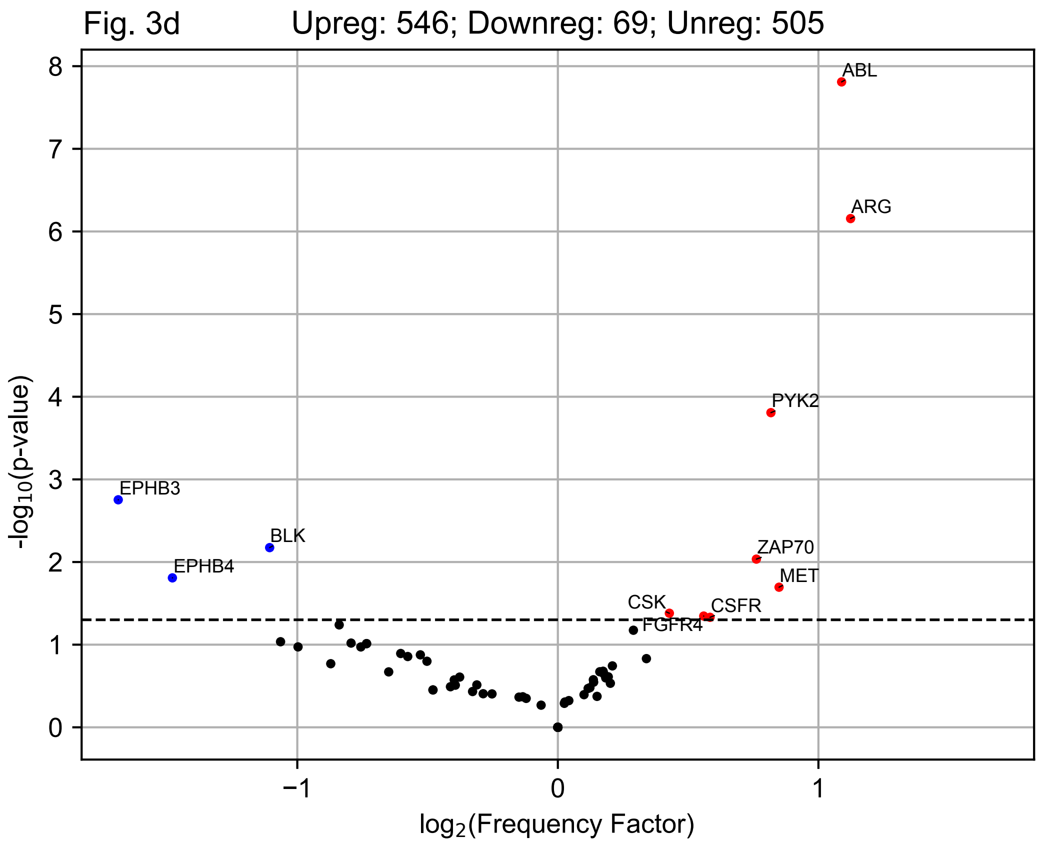
Fig. 3d
Upreg: 546; Downreg: 69; Unreg: 505
ABL
ARG
PYK2
EPHB3
BLK
EPHB4
ZAP70
MET
CSK
CSFR
FGFR4
8
7
6
5
4
3
2
1
0
−1
0
1
-log10(p-value)
log2(Frequency Factor)

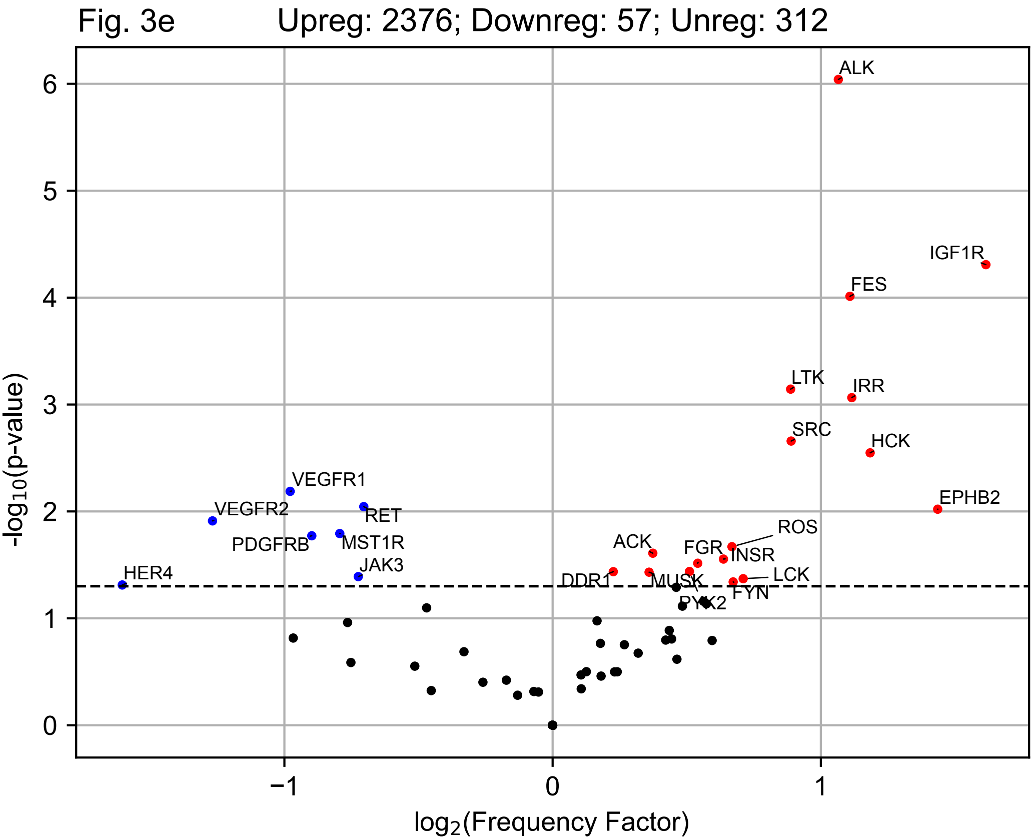

Fig. 3e
Upreg: 2376; Downreg: 57; Unreg: 312
-log10(p-value)
log2(Frequency Factor)
0
1
2
3
4
5
6
−1
0
1
ALK
IGF1R
FES
LTK
IRR
SRC
HCK
EPHB2
ROS
ACK
FGR
INSR
LCK
FYN
DDR1
MUSK
PYK2
VEGFR1
VEGFR2
RET
PDGFRB
MST1R
JAK3
HER4

Supplementary Figure 4: Fully annotated volcano plots presented in Fig. 4.

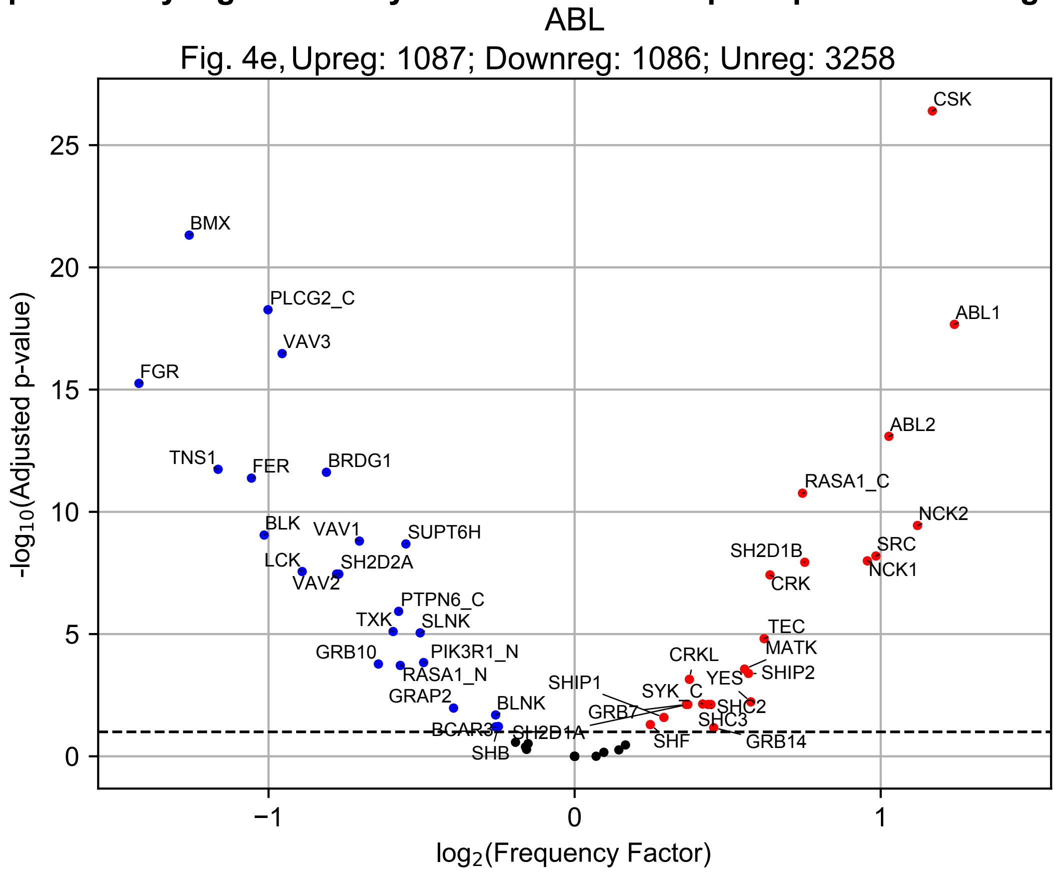

Fig. 4f

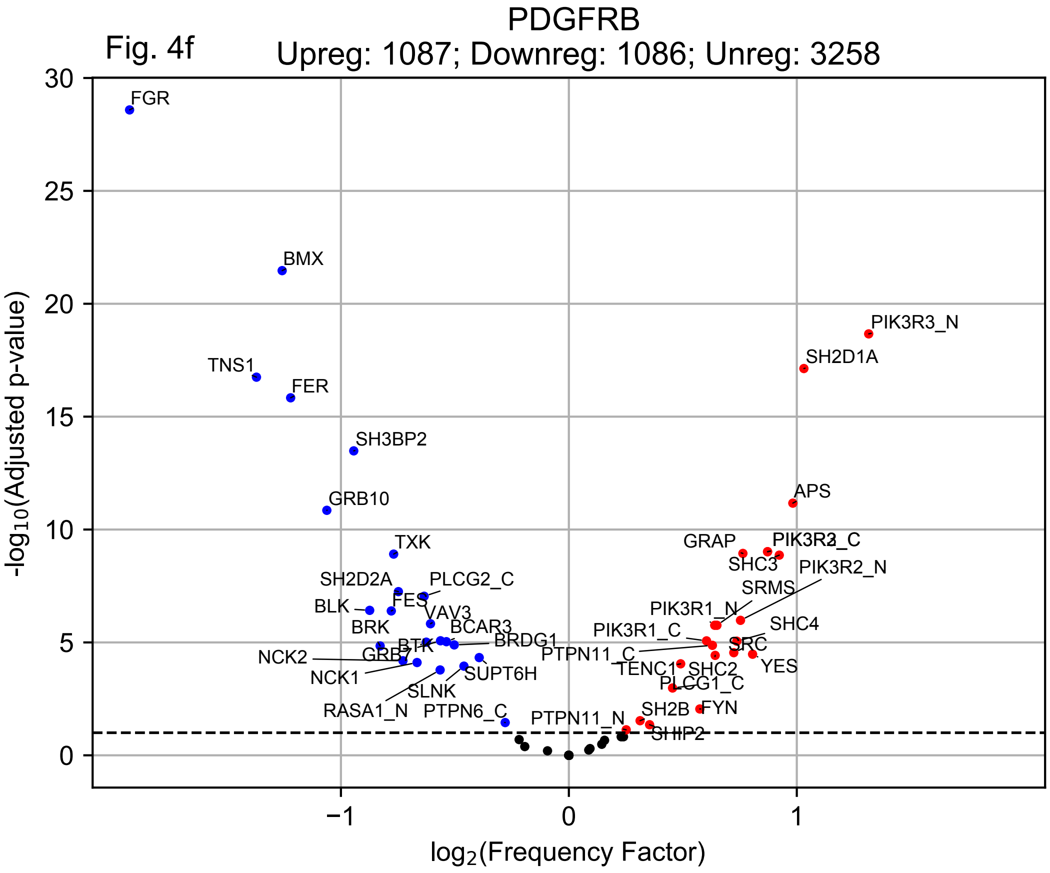
PDGFRB
Upreg: 1087; Downreg: 1086; Unreg: 3258
-log10(Adjusted p-value)
log2(Frequency Factor)
30
25
20
15
10
5
0
−1
0
1
FGR
BMX
TNS1
FER
SH3BP2
GRB10
TXK
SH2D2A
PLCG2_C
FES
BLK
VAV3
BRK
BCAR3
BRDG1
BTK
GRB7
NCK2
NCK1
SUPT6H
SLNK
RASA1_N
PTPN6_C
PTPN11_N
PIK3R3_N
SH2D1A
APS
GRAP
SHC3
PIK3R2_N
SRMS
PIK3R1_N
SHC4
PIK3R1_C
PTPN11_C
SRC
YES
TENC1
SHC2
PLCG1_C
SH2B
FYN
SHIP2

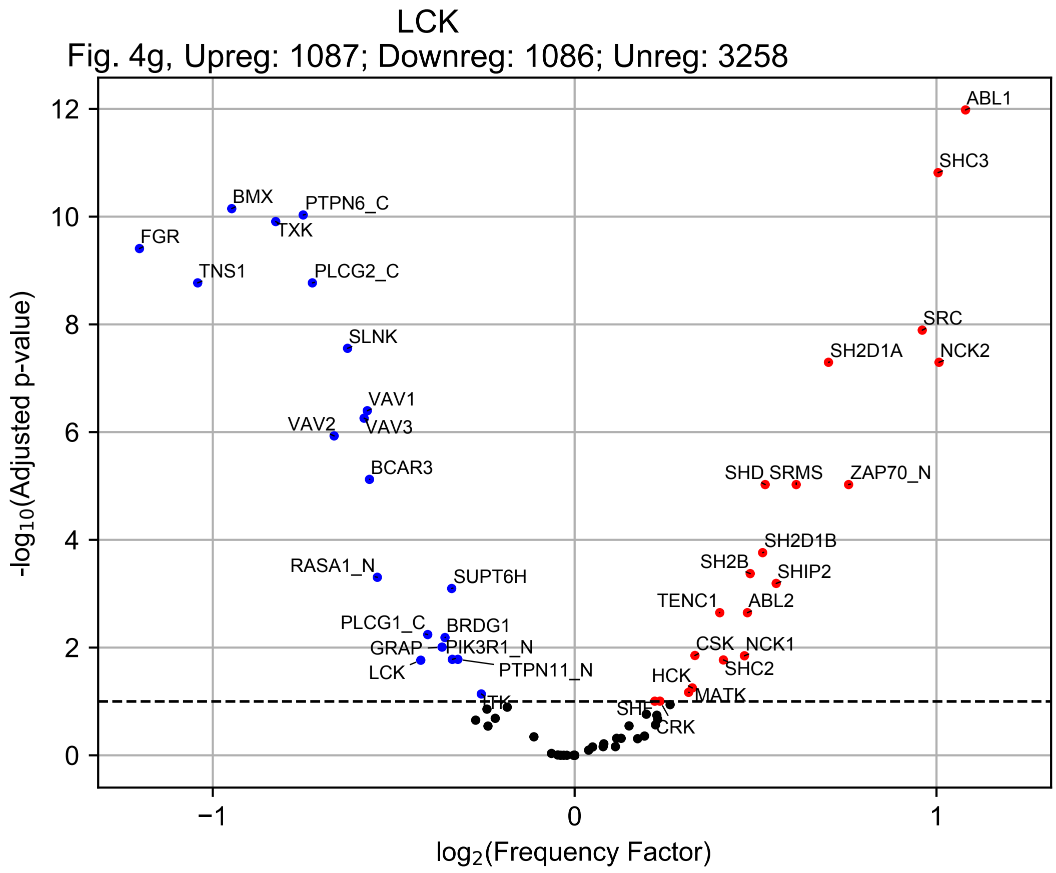
LCK
Fig. 4g, Upreg: 1087; Downreg: 1086; Unreg: 3258
-log₁₀(Adjusted p-value)
log₂(Frequency Factor)
ABL1
SHC3
BMX
PTPN6_C
TXK
FGR
TNS1
PLCG2_C
SRC
SH2D1A
NCK2
SLNK
VAV1
VAV2
VAV3
BCAR3
SHD
SRMS
ZAP70_N
SH2D1B
SH2B
SHIP2
RASA1_N
SUPT6H
TENC1
ABL2
PLCG1_C
BRDG1
GRAP
PIK3R1_N
LCK
PTPN11_N
CSK
NCK1
SHC2
HCK
MATK
ITK
SHF
CRK
12
10
8
6
4
2
0
−1
0
1

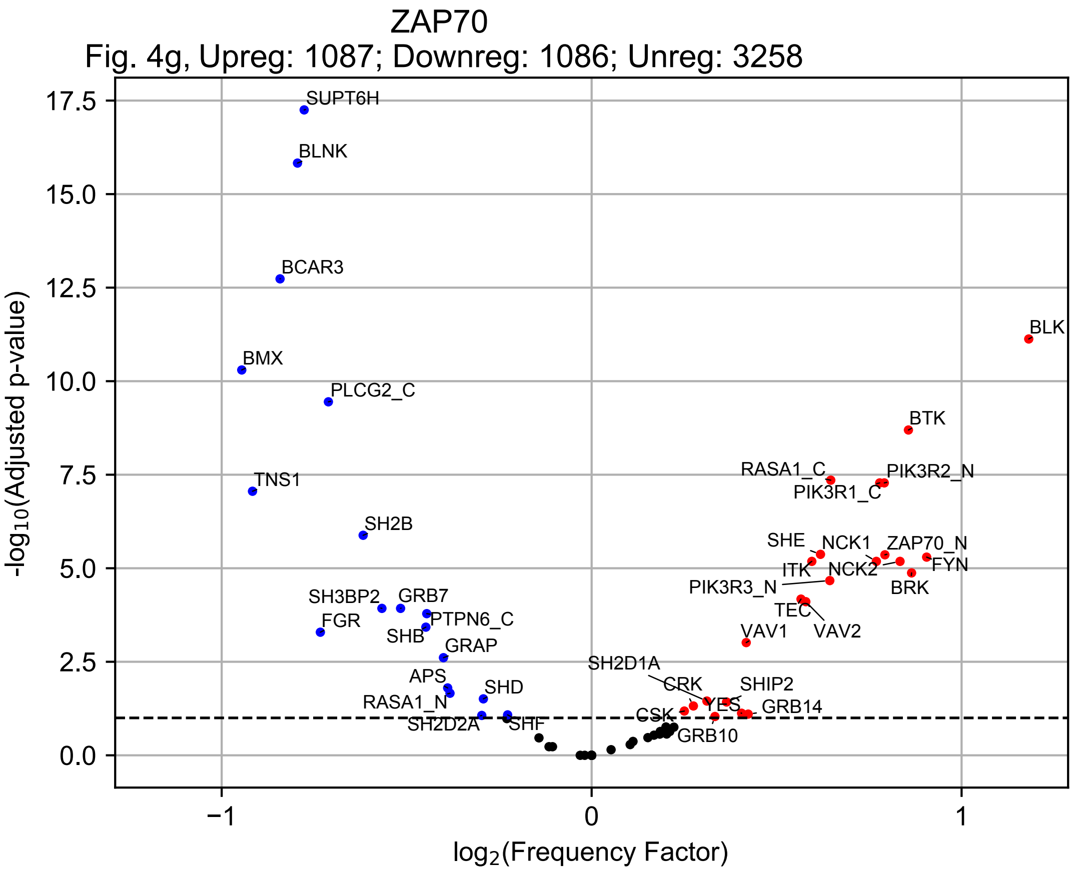
ZAP70
Fig. 4g, Upreg: 1087; Downreg: 1086; Unreg: 3258
-log10(Adjusted p-value)
log2(Frequency Factor)
17.5
15.0
12.5
10.0
7.5
5.0
2.5
0.0
−1
0
1
SUPT6H
BLNK
BCAR3
BMX
PLCG2_C
TNS1
SH2B
SH3BP2
GRB7
FGR
PTPN6_C
SHB
GRAP
APS
SHD
RASA1_N
SH2D2A
SHF
SH2D1A
CRK
CSK
YES
GRB10
SHIP2
GRB14
BLK
BTK
RASA1_C
PIK3R2_N
PIK3R1_C
SHE
NCK1
ZAP70_N
ITK
NCK2
FYN
PIK3R3_N
BRK
TEC
VAV2
VAV1

Supplementary Note 1

$$Raw\ Score_{Kin\ X} = \frac{\prod_{Pos} P_{Kin\ X}(AA, Position)}{\left(\frac{1}{\#Random\ AA}\right)^{length(positions)}}$$
